# Overcoming Therapy Resistance in Colorectal Cancer: Targeting the Rac1 Signaling Pathway as a Potential Therapeutic Approach

**DOI:** 10.3390/cells13211776

**Published:** 2024-10-26

**Authors:** Luciano E. Anselmino, Florencia Malizia, Aylén Avila, Nahuel Cesatti Laluce, Macarena Mamberto, Lucía C. Zanotti, Cecilia Farré, Vincent Sauzeau, Mauricio Menacho Márquez

**Affiliations:** 1 Instituto de Inmunología Clínica y Experimental de Rosario (IDICER, CONICET-UNR), Facultad de Ciencias Médicas (UNR), Rosario 2000, Argentina; anselmino@idicer-conicet.gob.ar (L.E.A.); flormalizia@gmail.com (F.M.); cesattilaluce@idicer-conicet.gob.ar (N.C.L.); mamberto@idicer-conicet.gob.ar (M.M.); zanotti@idicer-conicet.gob.ar (L.C.Z.); cfarre.cipreb@gmail.com (C.F.); 2 Instituto de Inmunología Clínica y Experimental, CONICET, Rosario 2000, Argentina; 3 Centro de Investigación y Producción de Reactivos Biológicos (CIPReB), Facultad de Ciencias Médicas (UNR), Suipacha 660, Rosario 2000, Argentina; aylen.avila@gmail.com; 4 Centro de Investigación del Cáncer de Rosario (CIC-R), Red de Investigación del Cáncer de Rosario (RICaR), Rosario 2000, Argentina; 5 Institut du Thorax, Inserm, CNRS, Université de Nantes, 44000 Nantes, France; vincent.sauzeau@inserm.fr

**Keywords:** colorectal cancer, resistance, small GTPases, Rac1, repositioning

## Abstract

Colorectal cancer (CRC) is the third most commonly diagnosed type of cancer worldwide and is responsible for numerous deaths. 5-fluorouracil (5-FU) is an effective chemotherapy drug commonly used in the treatment of CRC, either as monotherapy or in combination with other drugs. However, half of CRC cases are resistant to 5-FU-based therapies. To contribute to the understanding of the mechanisms underlying CRC resistance or recurrence after 5-FU-based therapies, we performed a comprehensive study integrating in silico, in vitro, and in vivo approaches. We identified differentially expressed genes and enrichment of pathways associated with recurrence after 5-FU-based therapies. Using these bioinformatics data as a starting point, we selected a group of drugs that restored 5-FU sensitivity to 5-FU resistant cells. Interestingly, treatment with the novel Rac1 inhibitor, 1A-116, reversed morphological changes associated with 5-FU resistance.. Moreover, our in vivo studies have shown that 1A-116 affected tumor growth and the development of metastasis. All our data allowed us to postulate that targeting Rac1 represents a promising avenue for the development of new treatments for patients with CRC resistant to 5-FU-based therapies.

## 1. Introduction

Colorectal cancer (CRC) is the third most commonly diagnosed type of cancer worldwide, accounting for ten percent of all malignant diagnoses and responsible for numerous deaths, ranking third in terms of incidence but second in terms of mortality [1], with estimations of increasing incidence for the next few years [2]. Increased risk of CRC is normally associated with age, western lifestyle, diet, personal history, and chronic intestinal diseases [3].

Most CRCs arise from previous adenomatous polyps. Although it remains uncertain the time it takes for an early adenoma to progress to CRC, the detection and removal of precancerous lesions before progressing to malignancy and metastasis provides a prevention strategy [1,4].

CRC treatment depends on factors such as the patient’s health, tumor size and location, and the presence of metastasis, but surgical removal is the most common option [5], followed by administration of adjuvant chemotherapy when there is local-regional, or distant invasion.

Since its discovery in 1957, chemotherapies based on 5-fluorouracil (5-FU, a fluorinated uracil analog) have remained the mainstay of adjuvant and palliative therapies for CRC patients [6]. 5-FU interferes with nucleoside metabolism by inhibiting the action of thymidylate synthase and missincorporating metabolites into DNA and RNA, leading to cytotoxicity and cell death [6,7,8]. Despite the clinical benefits and the extended use of 5-FU, response rates to 5-FU monotherapy are below 20% as most patients do not completely eliminate tumor cells, and tumor recurrence leads to poor outcomes [9]. To counteract this, various strategies have been developed to increase 5-FU effectiveness through the modulation of its intracellular and biochemical metabolism, mainly combining 5-FU with other cytotoxic drugs with different mechanisms of action. Combined chemotherapies FOLFOX (leucovorin + 5-FU + oxaliplatin), FOLFIRI (leucovorin + 5-FU + irinotecan), and FOLFOXIRI (leucovorin + 5-FU + oxaliplatin + irinotecan) are the most used in the clinic and have been used as the standard therapy for advanced CRC, increasing response rates up to 40–50% [10,11]. Unfortunately, despite the increased response, CRC patients’ disease-free survival has not been efficiently extended [12,13], and half of CRCs are resistant to 5-FU-based therapies. Therefore, there is a need for studies focusing on characterizing resistance-mediators biological factors or identification of biomarkers to define which CRC population is most likely to respond to 5-FU-based therapies [14,15,16].

One of the main causes of failure in cancer treatment is the development of drug resistance by cancer cells. This is a very serious phenomenon since it causes the recurrence of the disease or even death [17]. Therapy resistance occurs when diseases become tolerant to pharmaceutical treatments. This concept was first considered when bacteria became resistant to certain antibiotics, but similar mechanisms have since been found in other diseases, including cancer. Some resistance mechanisms are specific to each pathology, while others, such as drug efflux, can be observed at the microbial level and also in tumors, making them evolutionarily conserved [18]. Although many types of tumors are initially susceptible to chemotherapy, over time they may develop resistance through this and other mechanisms, such as DNA mutations and metabolic changes that promote the inhibition and degradation of drugs [19]. Tumor resistance is not limited to conventional chemotherapeutic drugs but also appears associated with the use of target therapies or biotherapies [20,21]. Consequently, numerous efforts are focused on exploring biomarkers that can predict or indicate the success of therapy.

In the case of CRC, 5-FU-based therapies increased response rates up to 40–50%; therefore, strategies to improve clinical outcomes are required. To address this, a deeper understanding of the mechanisms associated with resistance or recurrence after 5-FU-based therapies is imperative.

On the other hand, Rac1 (Ras-related C3 botulinum toxin substrate 1) is a key member of the Rho GTPases family. It is well known that Rac1 is a regulator of actin-based cytoskeletal dynamics, modulating cell adhesion, morphology, and movement. Rac1 is highly expressed in different tumor types and related to poor prognosis [22]. In tumors, it was described that Rac1 modulates cell cycle, apoptosis, proliferation, invasion, migration, and angiogenesis. Rac1 also plays a key role in anti-tumor therapy and participates in immune escape mediated by the tumor microenvironment [22]. Increasingly, studies are reporting the role of Rac1 as a potential target for tumor therapy [23].

To contribute to understanding the mechanisms underlying CRC resistance to therapies, we have conducted a study integrating in silico, in vitro, and in vivo approaches. First, we compared microarray gene expression data from 5-FU-treated CRC patients with and without recurrence after 5-FU monotherapy. Then, we extended the analysis to 5-FU-based therapies. Comparisons uncovered common enrichment pathways associated with chemotherapy resistance that allowed us to select drugs to overcome this phenomenon. Interestingly, inhibition of Rac1 by the 1A-116 compound decreases the growth of 5-FU-resistant CRC, sensitizes cells to 5-FU therapy, and prevents metastasis development, suggesting that therapies based on Rac1 inhibition could be of benefit to overcome therapy resistance.

## 2. Materials and Methods

### 2.1. Gene Expression Data Collection

**Figure 1:**
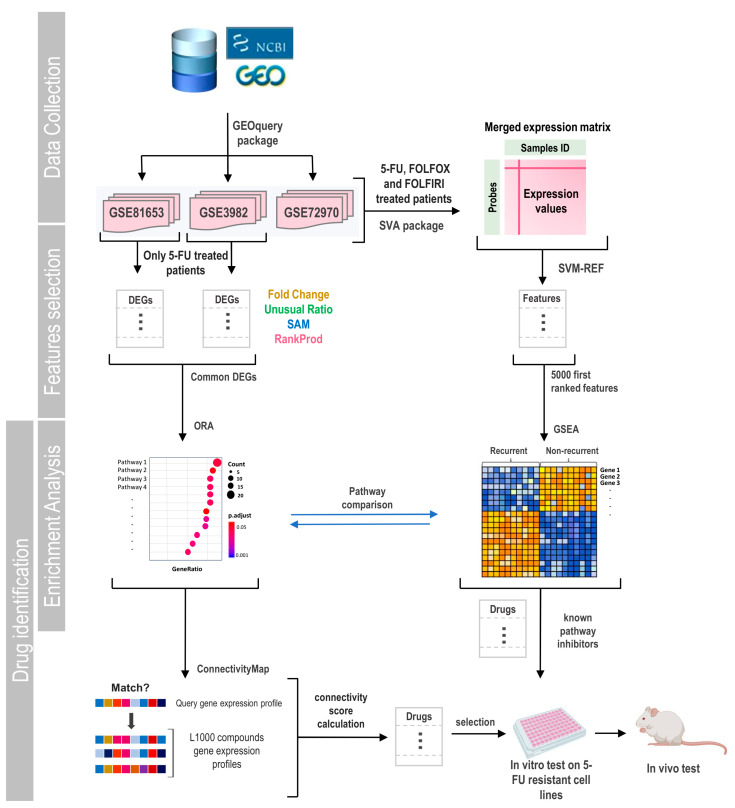
General flowchart of the experimental approach. In this work, three data series were selected from the GEO database. Studies contained gene expression data from tumors of CRC patients treated with different chemotherapies. Patients were followed up to record tumor relapse or death after treatment. First, we selected patients treated only with 5-FU and used different methods to obtain DEGs between groups with and without tumor relapse in each data series. Then, we performed an overrepresentation analysis of pathways (ORA) based on common identified DEGs. These genes were also used for screening of potential drugs able to reverse the expression profile associated with recurrence by the L1000 project of ConnectivityMap. Then we extended our studies by in-corporating patients treated with other 5-FU-based chemotherapies and constructing a gene ex-pression matrix by merging data from different studies. Using SVM-REF algorithm, genes were ordered based on their informative capacity among the phenotypes. With the 5000 most informa-tive genes, a GSEA analysis was carried out. Based on this analysis, we selected a set of drugs to test in vitro and in vivo.

Gene expression profiles of datasets GSE81653, GSE39582, and GSE72970 were downloaded from the NCBI Gene Expression Omnibus (GEO) database (https://www.ncbi.nlm.nih.gov/gds, accessed on 1 August 2024) using the GEOquery package (v2.72.0) [24,25]. These datasets contain gene expression profiling of tumor clinical samples from patients after exposure to 5-FU alone and 5-FU-based combined chemotherapy. Sample inclusion criteria for the analysis were: (1) not proceeding from studies of patients with familial hereditary polyposis; (2) including clinical information detailing the type of chemotherapy provided; and (3) presenting clinical information about tumor recurrence status. The expression values downloaded were normalized using the RMA (Robust Multichip Average) [26] method from the Affy (v1.82.0) or Oligo (v1.66.0) packages, depending on the chip model. Probe annotation was performed using the manufacturer-supplied annotation package: “hugene20sttranscriptcluster.db” [27] for the GSE81653 series and “hgu133plus2.db” [28] for the GSE39582 and GSE72970 series (a general flowchart of this work is presented in Figure 1).

If publicly available, information on clinicopathologic characteristics of patients was retrieved to explore their association with recurrent conditions using Fisher’s exact test; a significant association was considered for a *p*-value < 0.05.

### 2.2. Identification of Differentially Expressed Genes in Patients Treated with 5-FU Monotherapy

Data analysis was carried out using the facilities of the CCT-Rosario Computational Center, a member of the High Performance Computing National System (SNCAD, MincyT, Rosario, Argentina), where data were introduced into the R/Bioconductor environment (v3.18). Differentially expressed genes (DEGs) were identified in the GSE39582 and GSE81653 datasets independently. Each dataset was divided into two groups: (1) patients treated with 5-FU monotherapy with tumoral recurrence after surgical tumor resection, and (2) patients treated in the same way but without tumor recurrence. Detection platforms and sample sizes are shown in Table S1. A more detailed description of the selected samples is described in Table S2.

Four methods were used to obtain DEG groups; two exploratory methods: fold change (FC) and unusual ratio (UR); and two non-parametric algorithms: the Significance Analysis of Microarrays (SAM; using SAMr package v3.0) [29] and Rank Products (RP; using RankProd package v3.30.0 [30]. For DEGs selection, we used the cutoff criterion |log_2_fold change| > 1 for the FC method. For UR, any observation far from the mean by more than two standard deviations was considered atypical. For the non-parametric hypothesis tests, genes with |log2fold change| > 1 and FDR ≤ 0.01 were considered.

### 2.3. Enrichment Analysis

Reactome database (v88) signaling pathway enrichment analysis for DEGs was performed using the R package ReactomePA (v1.46.0) [31]. ReactomePA uses the hypergeometric model to assess whether the number of selected genes associated with a Reactome pathway is larger than expected. Pathways with an FDR < 0.05 were considered significantly enriched and were visualized using the dotplot tool. We generated an enrichment map and pre-clustered network using the enchmap and cnetplot tools to visualize relationships between pathways and highlight genes related to the more significant terms.

### 2.4. Identification of Gene Expression Profile-Reversing Compounds

A computational drug repositioning analysis was performed using query tool from CLUE (Connectivity map Linked User Environment: https://clue.io/query, accessed on 19 October 2024; Broad Institute, Cambridge, MA, USA; data version 1.1.1.2; software version 1.1.1.42), a cloud-based analysis platform that catalogs 473,647 expression signatures of human cell lines treated with 25,200 perturbagens [32]. CLUE computes a CMap score (tau) that measures the similarity of a queried gene set of up to 150 upregulated and 150 downregulated genes to existing drug-matched reference gene sets, from least similar or inverse (−100) to most similar (100). From an initial list of 426 DEGs, we selected 150 upregulated genes by screening for genes detected as DEGs by three or more methods in both data sets, as well as those associated with pathways with the highest enrichment score in the recurrent phenotype. Since the number of down-regulated DEGs was lower than 150, all of them were used. The reversing drugs of interest were selected using a negative threshold of CMap score.

### 2.5. Integrative Meta-Analysis

To explore whether the enriched pathways between patients with and without tumor recurrence after 5-FU treatment were maintained when patients treated with other types of chemotherapy were added to the expression matrix, we performed a meta-analysis. This technique is particularly useful for combining several datasets from the same disease when these are limited in size, therefore improving their statistical power. This time, instead of analyzing the data series independently, a large matrix was constructed by merging gene expression data from the GSE81653, GSE39582, and GSE72970 series. In addition to the patients treated with 5-FU already used in the previous analysis, others treated with FOLFIRI and FOLFOX were included. Data merging was performed using the empirical Bayes methods (ComBat, from the sva package v3.46.0) [33] to reduce confounding factors due to non-biological variations between the studies. Data about platforms and sample sizes are shown in Table S3. A more detailed description of the selected samples added to the analysis is found in Table S4.

### 2.6. Feature Selection

Feature selection is described as a process in which a subset of relevant features is selected from a larger data set. These features can be used for several purposes, such as model construction, differential analysis, enrichment exploration, etc. To select the most explanatory genes among the phenotypes from the combined gene expression matrix, we used the sigFeature package (v1.16.0) [34]. This package employs a combination of Vector Support Machine and Recursive Feature Elimination (SVM-RFE) algorithms to produce a ranked list of genes [35]. The SVM-RFE is a backward feature elimination technique that iteratively removes features based on SVM classifier weights. In each iteration, an SVM model is built based on the current features subset “F”, and the weight of each feature in “F” is calculated. The features are then ranked based on weight, and the bottom-ranked features are removed from F until it is empty. The top-ranked features that are discarded in the last iteration are considered the most informative between the phenotypes. The number of features retained in the analysis depends on their particular future use. In this work, we selected the top 5000 most informative features to improve their potential to predict the biological signature of the data set.

### 2.7. Gene Set Enrichment Analysis (GSEA)

GSEA is a computational method used to determine whether an *a priori* defined set of genes shows concordant and statistically significant differences between two biological states [36]. We employed the java GSEA Desktop Application v4.2.3 to perform the GSEA analysis on 5000 more informative features selected. The gene sets available from The Molecular Signatures Database 3.0 (MSigDB) were employed [37]; genesets composed of less than 15 or more than 500 genes were excluded. The phenotype label was set as “recurrent” vs. “non-recurrent”. The t-statistic mean of the genes was computed in each gene set using a permutation type test with 1000 replications. Up-regulated gene sets were defined by a normalized enrichment score (NES) > 0 and down-regulated by NES < 0. Gene sets with an FDR-*p* value ≤ 0.05 were chosen as significantly enriched.

### 2.8. Detection of Motifs and Transcriptional Factors

For the discovery of transcription factor binding sites (motifs) in the promoters of co-regulated genes, we used the Cytoscape (v 3.10.1) plug-in iRegulon (v1.3) [38,39]. A collection of 9713 position weight matrices (PWMs) was applied to analyze 10 kb centered around the transcription start site. DNA logos corresponding to each motif were extracted, and the main transcriptional factors binding to them and their sets of direct targets (metatargetnoma) were screened. Cut-off criteria used in the analysis were enrichment score threshold = 5, ROC threshold for AUC calculation = 0.03, rank threshold = 5000, minimal identity between orthologous genes = 0.05, FDR > 0.001, and normalized enrichment (NES) > 3.

### 2.9. Predictor Genes Detection

Important predictor genes from DEG list and the “reactome_signaling_by_rho_gtpases” gene set (https://www.gsea-msigdb.org/gsea/msigdb/cards/REACTOME_SIGNALING_BY_RHO_GTPASES, v7.3, accessed on 19 October 2024) were selected via least absolute shrinkage and selection operator (LASSO) logistic regression [40] in individual GEO series and in the merged matrix using the glmnet R package. To carry out the analysis, patients from each dataset were divided into training and validation sets in an 80–20 ratio, respectively. Optimal values for the penalty parameter λ were determined through 10-fold cross-validations. For model construction, we employed the lambda value giving minimal mean cross-validated error (lambda.min). The LASSO coefficient for recurrence predictor genes for each gene list was extracted. Receiver operating characteristic (ROC) curves were plotted to validate the prediction efficiency of the model using the ROCR (v 1.0.11) and pROC (v1.18.2) R packages [41,42]. We employed survival package (v3.5.7) [43] to perform a univariate Cox regression to assess the effect of the change in expression of individual predictor genes on the survival of patients in each dataset; genes with a LogRank < 0.01 were retained.

### 2.10. PPI Network Construction and Hub Gene Identification

The PPI network was constructed using the STRING public online database [44] and exported to Cytoscape (v3.10.1) for viewing and analysis [39]. Cytoscape plug-in CytoHubba (v0.1) [45] was employed to retrieve the top 20 hub genes based on the maximal clique centrality (MCC) algorithm. To expand the network, main interactor genes were retrieved using the network expansion function of the StringApp (v2.1.1) plug-in. The top 20 interactors with a selectivity of 0.5 were included in the network.

### 2.11. Drugs

For each drug, a 10 mM stock solution was prepared and stored at −20 °C. Ivermectin (Parafarm, Argentina) was dissolved in a 50% DMSO solution; the concentration of DMSO in the final dilutions did not exceed 0.03%. Amitriptyline (Parafarm) was dissolved in sterile distilled water. Commercial 20 mg/mL irinotecan solution (Kemex, Argentina) and 50 mg/mL 5-FU solution (Fada Pharma, Argentina) were diluted in sterile distilled water to reach the final stock concentration. 1A-116 [46] was kindly provided by Dr. Georgina Cardama (Laboratorio de Oncología Molecular, Universidad Nacional de Quilmes). 1A-116 stock was prepared by dissolving the drug in acidic water (sterile distilled water adjusted to pH 1–2 using a 100 mM HCl solution). Once dissolved, the pH of the final solution was adjusted to 5.5–6 with 100 mM NaOH and filtered. Unless indicated otherwise, the doses used of drugs were 5 µM 5-FU, 20 µM amitriptyline, 30 µM irinotecan, 15 µM ivermectin, and 20 µM 1A-116.

### 2.12. Cell Culture

5-FU resistant cell lines CT26^5FUR^, HCT116^5FUR^, or HT29^5FUR^ were produced in our laboratory as described before [47] from CT26 (chemically induced BALB/c mice-derived colorectal carcinoma), HCT116 (male human colon adenocarcinoma), and HT29 (female human colon adenocarcinoma) cells, respectively. Cells were cultured in DMEM (HCT116 and HT29) or RPMI (CT26) media, supplemented with 10% fetal bovine serum (FBS; Natocor, Argentina), penicillin (10 μg/mL), streptomycin (100 μg/mL), and L-glutamine (2 mM, DMEM medium). Cells were maintained at 37 °C in a 5% CO_2_ atmosphere and routinely tested for mycoplasma.

### 2.13. Cells Immunostaining Techniques

7 × 10^3^ cells were cultured in coverslips for 24 h, treated for 36 h, fixed in 4% paraformaldehyde, permeabilized with 0.5% PBS-Triton, and blocked with BSA (2%; Sigma, St. Louis, MO, USA). Cells were first incubated with α-Tubulin (1:1000 dilution, Sigma) and β-catenin (sc-59737, 1/50 dilution; Santa Cruz, Dallas, TX, USA) or E-Cadherin (67A4) (sc-21791, 1/50 dilution, Santa Cruz), and then with Alexa Fluor 488-conjugated secondary antibody (1:500 dilution; Invitrogen, Carlsbad, CA, USA), and counterstained with DAPI (1:10,000 dilution, Sigma) and phalloidin-rhodamine conjugate (1:2000 dilution, Invitrogen) to observe cell nucleus and actin skeleton, respectively. Coverslips were fixed to a slide with Mowiol and observed under a Nikon Ti2E fluorescent microscope or a Zeiss LSM880 confocal microscope. Cell and nuclear areas were measured with the ImageJ software (v1.54f).

### 2.14. Viability Assays

For in vitro cell viability assays, 1 × 10^4^ CT26^5FUR^, HCT116^5FUR^, or HT29^5FUR^ cells were seeded on 96-well plates and incubated for 36 h at 37 °C in a 5% CO_2_ atmosphere with increasing doses of the individual drugs and combinations of selected doses with 5 µM 5-FU. Controls were treated in the same way with corresponding vehicles for each drug. After the incubation time, treatments were removed, and an MTT assay (Sigma) was performed as described before [47]. Viability was expressed as the percentage of control, untreated samples. The concentration of drugs that decreased cell proliferation by 50% (IC50) as compared to controls was calculated with GraphPad Prism v8.0 (GraphPad Sofware, La Jolla, CA, USA). When indicated, viable cells were counted using Trypan Blue (0.4%; Sigma), and viability was expressed as the percentage of control untreated samples. Drug doses used to analyze 5-FU resistance reversal were selected from IC50 curves, selecting the smallest dose with minimal effect on cell viability.

### 2.15. Immunoblotting

CRC cells were seeded in 60 mm plates until they achieved 80% confluence. Cells were lysed with RIPA buffer containing protease inhibitor cocktail (Roche Diagnostics, Mannheim, Germany) and then detached from the plates to collect protein extracts. Protein levels were quantified using the Lowry assay, and 40 µg of protein per sample was denatured for 5 min at 95 °C in SDS-PAGE sample buffer before being loaded onto gels. Samples were resolved on 8% SDS-polyacrylamide gels and transferred to PVDF membranes (Amersham Hybond P, GE Healthcare Life Sciences, Little Chalfont, Bucks, UK) for 90 min with constant current at 4 °C. Membranes were blocked with 1% (*w*/*v*) BSA in TBS-Tween (50 mM Tris, 150 mM NaCl, 0.05% Tween, pH 7.5) for 60 min at room temperature and then incubated with the specified primary antibody overnight at 4 °C. The primary antibodies used included β-catenin (1:1000 dilution; BD Biosciences, San Jose, CA, USA) and E-cadherin (1:1000 dilution, BD Biosciences). After three washes with TBS-Tween to remove the primary antibodies, membranes were incubated with the appropriate peroxidase-conjugated secondary antibody (1:5000 dilution; BioRad, Hercules, CA, USA) for one hour at room temperature. Detection was carried out using chemiluminescence (Bio-Lumina; Kalium Technologies, Oklahoma City, OK, USA) and imaged with a Licor C-Digit Blot Scanner (LI-COR Biosciences, Lincoln, NE, USA) according to the manufacturer’s instructions. Quantification was performed by densitometry using Image J 1.8.0 software. Cropped images are displayed in the main figures, with full-length membranes shown in the Supplementary Information.

### 2.16. GTP-Rac1 Pull-Down Assay

The GST-Pak1 pull-down experiment to determine Rac1 activity in cells was performed as indicated [48]. Briefly, exponentially growing cells were harvested and resuspended in lysis buffer containing 20 mM Tris-HCl [pH 7.5], 150 mM NaCl, 5 mM MgCl_2_, 0.5% Triton X-100, 10 mM beta-glycerophosphate, 1 mM DTT, Complete (Roche, Basel, Switzerland), and 10 µg of GST fusion protein containing the Pak1 Rac1 binding domain (GST-Pak1 RBD, bacterially expressed). After incubations for 10 min on ice, cell lysates were pre-cleared by centrifugation at 14,000 rpm for 10 min at 4 °C, and supernatants were incubated with glutathione-Sepharose beads (GE Healthcare Life Biosciences) for 1h at 4 °C under gentle rotation. After extensive washes in lysis buffer, protein complexes were released by boiling in SDS-PAGE sample buffer, separated electrophoretically, transferred onto nitrocellulose filters, and analyzed by immunoblotting using an anti-Rac1 antibody (1:1000 dilution, BD Biosciences). GTP-Rac1 levels were quantified with the ImageJ analysis software using as normalizing control the total levels of Rac1 found in each cell lysate.

### 2.17. Animal Studies

Eight-week-old BALB/c female mice were obtained from the School of Veterinary Sciences at the National University of La Plata and treated in accordance with the Canadian Council on Animal Care and ARRIVE guidelines. Animals were maintained in the CIPReB facilities (Centro de Investigación y Producción de Reactivos Biológicos, Medicine School, National University of Rosario). Viable cells (1 × 10^6^ CT26^5FUR^) were resuspended in PBS (100 μL) and injected subcutaneously into the right flank of each animal. For all experiments, mice were distributed and treated as follows: control, consist in a daily intraperitoneal (ip) administration of vehicle (1% absolute alcohol solution); ivermectin, ip administration of 2 mg/kgBW/day (dissolved in absolute alcohol and then diluted in water to the final concentration); 1A-116, ip administration of 5 mg/kg BW/day in sterile water; 5-FU, ip administration 20 mg/kg BW/week in sterile water; iver  +  5-FU, ip administration of ivermectin and 5-FU treatments; 1A-116 + 5-FU, ip administration of 1A-116 and 5-FU treatments.

Animals were periodically weighed and checked for changes in skin, fur, eyes, secretions, excretions, and autonomic activity (lacrimation, pilo-erection, unusual respiratory pattern, movement, etc.).

Tumor volumes were calculated as V  =  0.4ab^2^, where a is the measurement of the tumor along its longest axis and b is its shortest. When any of the groups reached the ethically permitted tumor volume, the animals were euthanized. Lungs, spleens and tumors were removed, fixed, and stained with hematoxylin-eosin (H&E) for histological evaluation and detection and counting of micrometastases in an Olympus BX40 microscope (Olympus Corporation, Tokyo, Japan).

For intrasplenic inoculation of cells, mice were anesthetized by intraperitoneal injection of acepromazine/ketamine/midazolam (50 mg/kg, 100 mg/kg, and 50 mg/kg, respectively). A small incision was made to access the spleen and allow injection of 1 × 10^6^ CT26^5FUR^ viable cells resuspended in PBS (100 μL). Two days after surgery, animals were distributed in groups as described, and treatments were initiated. Three weeks after injection, animals were sacrificed for collection and weighing of spleens and livers.

For subcutaneous tumor development, 2 independent experimental rounds were performed. For the first round, N =  4/group; for the second round, N = 6/group. For intrasplenic injection, the number of animals used was N = 6/group.

### 2.18. Statistical Analysis

Statistical analyses were carried out using the GraphPad Prism 8.0 software (GraphPad Software, Inc., La Jolla, CA, USA). Single comparisons between two groups were performed with the Student's t-test, whereas for multiple comparisons, the ANOVA followed by Tukey’s multiple comparisons post-test was employed. The correlation between the two variables was assessed with Spearman’s rank correlation coefficient (r). For differential gene expression analysis, statistical significance was tested with the Student’s *t*-test followed by a false discovery rate (FDR) correction with the Benjamini–Hochberg procedure. Survival analysis was implemented according to the Kaplan–Meier method and log-rank test. Overall survival (OS) was defined as the time between the date of surgery and the date of death or the date of the last follow-up. In all cases, *p*-values less than 0.05 were considered statistically significant and were marked with an asterisk as follows: *, *p* ≤ 0.05; **, *p* ≤ 0.01; ***, *p* ≤ 0.001.

## 3. Results

### 3.1. Identification of Differentially Expressed Genes and Key Pathways in Recurrent CRC After 5-FU Monotherapy

To identify DEGs and key pathways associated with 5-FU resistance, raw data of the datasets GSE39582 and GSE81653 were downloaded from the GEO database. Each dataset was normalized using the RMA method (Figure S1A,B). Dataset GSE39582 contained a total of 585 samples, 82 of which met the requirements described in Section 2.1. Dataset GSE81653 contained 593 samples, 192 of which met the requirements. The selected patients from each study were divided into patients with and without tumor relapse groups. Before performing the analysis, probes with intensity values close to chip background were filtered and discarded (the proportion of probes removed is shown in Figure S1C,D).

**Figure 2:**
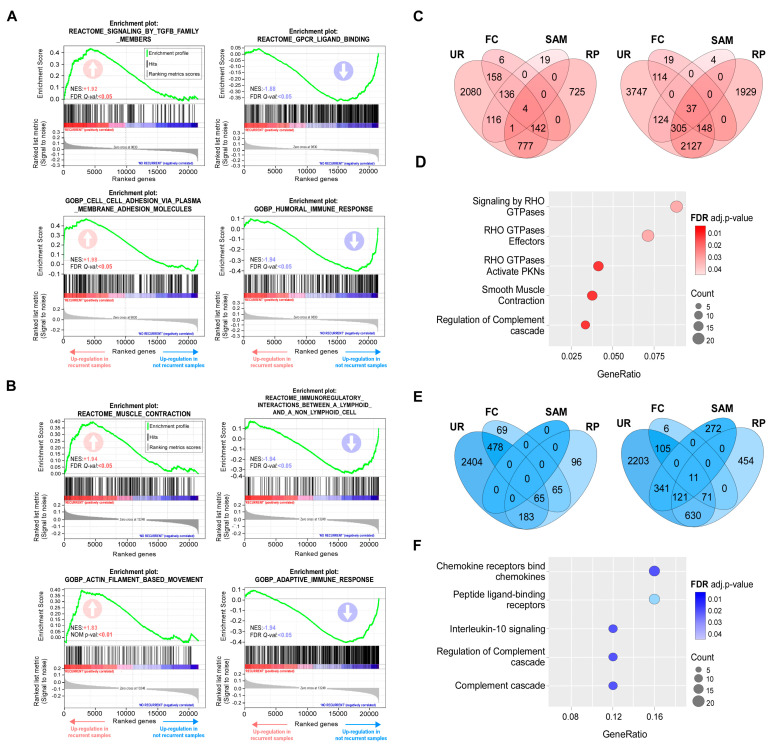
Gene set enrichments and DEGs associated with 5-FU monotherapy resistance. (**A**,**B**) Representative upregulated (**left**) and downregulated (**right**) gene sets in the recurrent phenotype according to Gene Set Enrichment Analysis (GSEA) for selected 5-FU-treated patients from the GEO dataset (**A**) GSE39582 and (**B**) GSE81653. NES and FDR values are indicated within each graph. Positive and negative enrichments are indicated with upward- and downward-pointing arrows, respectively. (**C**–**F**) DEGs analysis. Venn diagrams of overexpressed (**C**) and underexpressed (**E**) genes identified by four statistical methods (FC: Fold Change; RP: Rank Product; SAM: Significance Analysis of Microarray; UR: Unusual Ratio) for series GSE39582 (left diagram) and GSE81653 (right diagram); intersections indicate genes detected by two or more methods. (**D**) Reactome functional classification of upregulated (**D**) and downregulated (**F**) genes detected as DEGs in both datasets. Dot size is proportional to the number of genes associated with a term. Dot color intensity indicates the adjusted *p*-value resulting from the over-representation analysis. This graph displays only significant terms (*p* < 0.05).

In order to explore the putative association between recurrence and clinical parameters, correlation was assessed using Fisher’s exact test. The results indicated that 5-FU resistance was not associated with age, TNM stage (Tumor, Node, Metastasis staging system), or the main mutations described for CRC (KRAS, TP53, and BRAF; Table S5). As no correlation was noted, we performed enrichment analysis of gene sets associated with recurrent and non-recurrent phenotypes for both datasets. Through this approach, we observed that coordinated expression of genes grouped in categories linked to cell adhesion and migration was associated with a recurrent phenotype, while a good response to 5-FU monotherapy correlated with immune system and complement activation (Figure 2A,B and Tables S6 and S7).

In parallel, we aimed to identify DEGs associated with the 5-FU-resistant phenotype. To select DEGs in each dataset independently, we employed four methods: two exploratory (fold change [FC] and unusual ratio [UR]) and two non-parametric hypothesis tests (RankProd [RP] and the Significance Analysis of Microarrays [SAM]). The cut-off criteria for DEGs selection were described in Materials and Methods. DEGs lists were compared to extract common genes detected by at least two methods in both studies. The number of genes obtained by each method in each dataset was represented by a Venn diagram (Figure 2C,E). A total of 388 upregulated and 39 downregulated common DEGs were selected (Table S8). In order to detect pathways enriched in recurrent phenotypes, we performed an over-representation analysis (ORA). The results of this analysis indicated that the main upregulated pathways were associated with signaling mediated by Rho GTPase proteins and their effectors (Figure 2D), while among the downregulated pathways were found those related to cytokine receptors, receptors binding to peptide ligands, and the immune response (Figure 2F).

**Figure 3:**
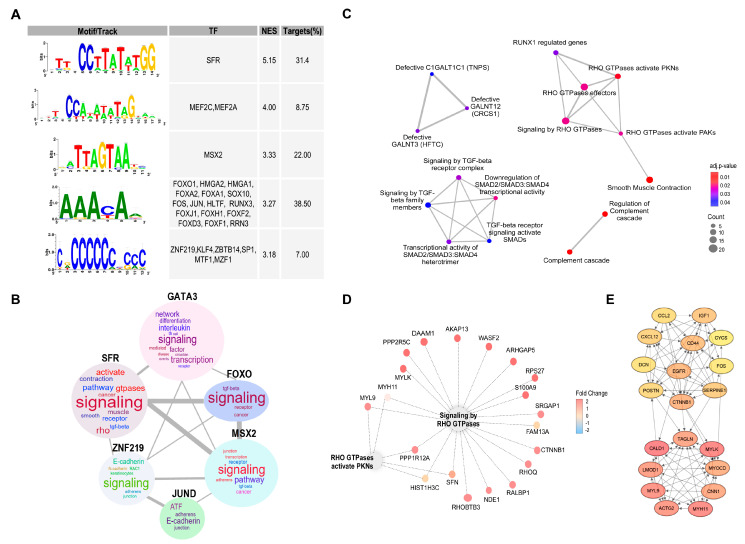
Regulation of DEGs associated with 5-FU-resistant phenotype. (**A**) Transcription factor binding sites enriched in promoter regions of upregulated genes in the 5-FU recurrent transcriptome. NES and target gene percentages are indicated. The motif/tracks column shows sequence logos. The horizontal axis represents base positions (Adenine: green, Cytosine: blue, Thymine: red, Guanine: orange) in the DNA, with the 3' end indicating strand orientation. The vertical axis shows information in bits, and letter height reflects base frequency at each position. (**B**) Word cloud showing the most frequent terms in the enrichment analysis result for each group of genes controlled by a transcriptional factor. The size of the word is proportional to the frequency the term appeared in the over-representation analysis (*p* > 0.05). (**C**) Enrichment map for visualizing the pathway/function relationships of DEGs associated with recurrence. Dot size is proportional to the number of genes associated with a term. Dot color intensity indicates the adjusted *p*-value resulting from the over-representation analysis. The thickness of the gray lines represents the level of connection between pathways. This graph displays only significant terms (*p* < 0.05). (**D**) Cnetplot showing the DEGs related to the RHO GTPases pathway; dot color indicates the fold change for the recurrent condition. (**E**) Top 20 hub genes identified by the CytoHubba Cytoscape plugin. A greater red color intensity is associated with the highest number of connections in the total DEGs PPI network.

Transcription factors are key regulators of biological processes that function by binding to gene regulatory regions. Each transcription factor recognizes a collection of DNA sequences or binding sites that can be represented as motifs. Motif characterization is important for understanding the regulatory functions of transcription factors shaping gene regulatory networks. To go further in characterizing expression changes associated to resistance, we look for common transcriptional factors binding sites present at the promotor regions of upregulated genes, finding that the most relevant factors include Serum Response Factor (SRF), Myocyte Enhancer Factor 2C and 2A (MEF2C, MEF2A), Msh Homeobox 2 (MSX2), and common motifs were found to FOXO1, HMGA2, HMGA1, FOXA2. FOXA1. SOX10, FOS, JUN, HLTF, and RUNX3 (Figure 3A). Genes specifically regulated by each transcription factor were extracted to perform a functional enrichment analysis (*p* < 0.05) through a word cloud graph, which indicates that the most relevant cellular processes modulated are related to signaling, GTPases, and E-cadherin (Figure 3B).

To visualize the most relevant relationships between pathways and functions associated with the 5-FU-resistant phenotype, we constructed an enrichment map that indicates over-representation of two particular clusters: Rho GTPases and TGF-beta signaling (Figure 3C,D). 5-FU-resitance-associated DEGs are highly connected genes by 20 hub genes, as visualized in Figure 3E.

### 3.2. Determinants of Resistance to 5-FU-Based Therapies

**Figure 4:**
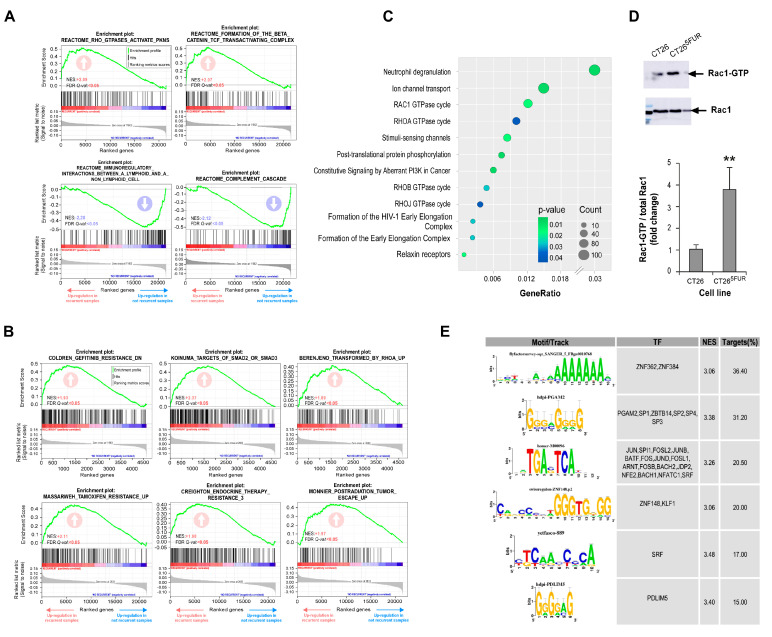
Gene set enrichments associated with resistance to 5-FU-based therapies. (**A**) Gene Set Enrichment Analysis (GSEA) associated with the transcriptome of recurrent phenotype following 5-FU-based chemotherapies using the Reactome database. (**B**) GSEA associated with the 5000 most explanatory genes in the recurrent phenotype after 5-FU-based chemotherapies selected by the Recursive Feature Elimination algorithm (RFE); gene sets from ‘c2.cgp.v2023.1.Hs.symbols.gmt’ database were employed to perform the analysis. The normalized enrichment scores (NES) and false discovery rate q-values (FDR q-val) are indicated within each graph. Positive and negative enrichments are specified by upward- and downward-pointing arrows, respectively. (**C**) over-representation analysis (ORA) of the 5000 most explanatory genes selected by RFE. Dot size is proportional to the number of genes associated with a term. Dot color indicates the *p*-value resulting from the ORA analysis. (**D**) Rac1 activation in control (CT26) and 5-FU resistant (CT26^5FUR^) cells determined by pull-down assays. Levels of Rac1-GTP and total Rac1 were analyzed by Western blot (upper panel) and quantified by ImageJ (lower panel). (**E**) Transcription factor binding sites found enriched in the promoter regions of 5000 RFE retained genes. The NES and percentage of regulated genes (targets) are also indicated. The motif column shows sequence logos. The logos horizontal axis represents base positions (Adenine: green, Cytosine: blue, Thymine: red, Guanine: orange) in the DNA, with the 3' end indicating strand orientation. The vertical axis shows information in bits, and letter height reflects base frequency at each position. ** *p* ≤ 0.01.

To go further in the search for determinants of resistance in CRC, we decided to extend our approach to databases of patients receiving therapies based on 5-FU, including FOLFOX and FOLFIRI. This approach allowed us to increase our samples to 567 to perform more robust analyses. As in the case of 5-FU monotherapy, no evident correlation was observed between resistance and available clinical parameters (Table S9). Unbiased GSEAs associated with the transcriptome of recurrent phenotype following 5-FU-based chemotherapies using Reactome, GO, and KEGG databases indicated a positive enrichment for Rho GTPases that activate PKNs and the formation of β-catenin:TCF transactivating complex and a negative enrichment for immunoregulatory interactions between lymphoid and non-lymphoid cells and complement cascades (Figure 4A, Table S10). Moreover, after selecting the 5000 most explanatory genes in the recurrent phenotype after 5-FU-based chemotherapies by the Recursive Feature Elimination algorithm (RFE) and a chemical and genetic perturbations database (MSigDB) we found a positive enrichment of gene sets associated with resistance to other therapies generally used to treat tumors other than CRC, such as gefitinib, tamoxifen, gemcitabine, and radiation (Figure 4B, Table S11), indicating that some of the identified pathways could be orchestrating general resistance mechanisms. Surprisingly, pathways overrepresented in this 5000 subset were related to neutrophil degranulation and Rho GTPases, among which Rac1 seems to be the most relevant (Figure 4C). Consistently, through pull-down experiments in CRC cells, we observed a significant increase in Rac1 activity associated with 5-FU resistance (Figure 4D). As it was the case for 5-FU monotherapy, we found binding motifs for SRF, JUN, and FOS transcription factors present at the promotor regions of upregulated genes (Figure 4E) associated with 5-FU-based therapy resistance.

### 3.3. Selection of Drugs to Overcome 5-FU Resistance in CRC

In order to overcome 5-FU resistance, we searched for compounds with the ability to reverse the expression of genes and pathways identified in our previous analysis by LINCS. This tool allows entering 150 upregulated and 150 downregulated genes. For 5-FU monotherapy, we used all common downregulated genes as they were less than 150. To reduce the number of upregulated genes to 150, we selected DEGs detected by three or more statistical methods (in both databases) and those with the higher association (with a lower *p* value) with overrepresented pathways in functional enrichment analysis (Table S12). The platform detected similar and opposite expression profiles in nine cell lines, including HT29.

Through this approach, we obtained a list of compounds that could potentially reverse the 5-FU resistant phenotype (Table S13). Some of these compounds are currently used in the clinic combined with 5-FU, such as irinotecan [49,50] or to treat other types of recurrent cancers, such as topotecan [51,52]; but we also found other compounds such as a norepinephrine inhibitor (amitriptyline), MEK inhibitors, a COX inhibitor, ivermectin, and the Rho-associated kinase inhibitor (Rockout), among others. Accordingly, cell perturbations that could reverse the expression profile associated with 5-FU resistance determined by the same platform include inhibition of Raf, MEK, PKC, or Src and loss of function of Rho GTPases activating proteins (Table S14).

### 3.4. Ivermectin and the Rac1 Inhibitor 1A-116 Restore 5-FU Sensitivity to 5-FU Resistant Cells

As drug repositioning provides cheaper, effective, and safe drugs with fewer side effects and speeds up drug development, we focused on irinotecan, amitriptyline, and ivermectin to confirm by cell studies their potential to sensitize 5-FU-resistant CRC cells. In parallel, as our studies highlighted a prominent role of the Rho GTPase Rac1 on 5-FU-resistance modulation, we decided to explore the effects of 1A-116, a novel Rac1 inhibitor previously reported to be promising to treat other cancer types [46,53,54,55]. To characterize the effect of these drugs on resistance, we used three 5-FU resistant cell lines generated as described before [47]. IC50 values for each selected drug were calculated on sensitive and resistant cell lines (Table S15).

**Figure 5:**
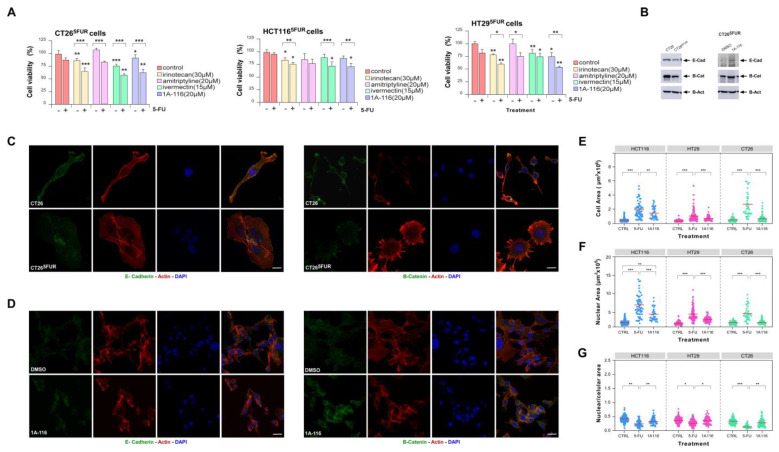
Characterization of 5-FU resistant cells and viability assays. (**A**) Viability of CT26^5FUR^, HCT116^5FUR^, and HT29^5FUR^ cells treated with selected drugs individually and in combination with 5µM 5-FU (N = 3). (**B**) Representative immunoblots showing modulation of epithelial marker expression associated with generation of resistance (**left** panel) and reversion of the expression after Rac1 inhibition (**right** panel). For loading control, we used the abundance of endogenous beta-actin (E-Cad: E-Cadherin; B-Cat: β-catenin; B-Act: beta-actin). (**C**,**D**) Confocal images for immunofluorescent detection of E-Cadherin (green color, left panel) and β-catenin (green color, right panel) in CRC cells counterstained with F-actin and nuclei (red and blue colors, respectively, scale bar = 10 μm). (**E**–**G**) Cells area, nuclei area, and nucleus/cell area relationship were quantified for each cell line (N > 50 per treatment). The red line indicates the median of the distribution. *, *p* ≤ 0.05; **, *p* ≤ 0.01; ***, *p* ≤ 0.001.

To evaluate the potential effect of 1A-116 and selected repurposing drugs on reversing resistance of CRC cells, we treated resistant cells with 5-FU in the presence of each drug. For all the assays, we selected doses for each drug with a minimal statistical effect on cell viability as evaluated by MTT-based assays (Figure S2 and Table S15). We found that incubation of cells with irinotecan, ivermectin, and 1A-116 improved responses to 5-FU in all cell lines tested (Figure 5A), while amitriptyline only had a partial effect on HT29 and CT26-resistant cells. For 1A-116 treatment, we confirmed the inhibition of Rac1 activation by this compound and its effect on reversing 5-FU resistance by counting viable cells after treatment (Figure S3A,B).

In vitro generation of 5-FU resistance is associated with cell morphological changes, as it was previously described for several cell lines, including CRC [56,57,58,59]. Previous reports also described that resistance to 5-FU in CRC cells promotes the loss of epithelial markers [58,59]. To confirm the link between resistance acquisition and EMT, we analyzed the expression of epithelial markers in CRC cells, validating the loss of E-Cadherin and β-catenin expression in resistance cells (Figure 5B,C). To assess morphological changes associated with 5-FU resistance, we performed nuclear, cytoskeletal, and microtubular staining of control and 5-FU-resistant cells (Figure S3D,E). In order to quantify changes in morphology, we measured nuclear and cell areas for each condition, noting a significant increase in both parameters for resistant cells (Figure 5E–G). Indeed, the relationship between nucleus/cytoplasm decreased in resistant cells, and cytoskeletal architecture changes became evident. Interestingly, treatment with the Rac1 inhibitor 1A-116 increased the expression of β-catenin and E-cadherin (Figure 5B,D) compared to untreated cells and reversed the morphological changes observed in the resistant phenotype, making the cells more similar to 5-FU-sensitive ones (Figure 5E–G and Figure S3B,C). This suggests a critical role for Rac1 activation in triggering events that lead to EMT-dependent 5-FU resistance in CRC.

### 3.5. Rac1 Inhibitor 1A-116 Reduces the Growth of CRC Resistant Cells, Sensitizes Them to 5-FU and Prevents Metastasis Development

**Figure 6:**
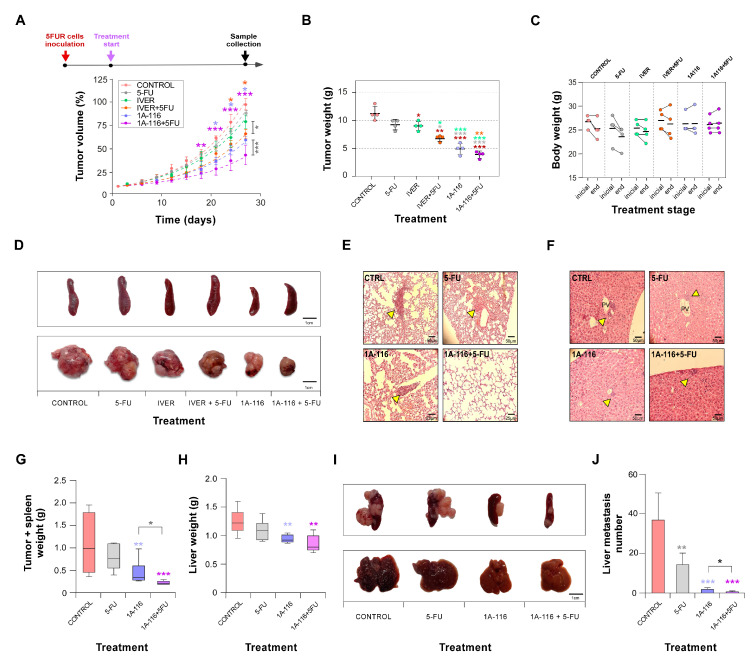
Ivermectin and 1A-116 in vivo studies. (**A**) BALB/c mice were subcutaneously challenged with CT26^5FUR^ cells. Ten days later, tumors became evident, and animals were randomly distributed in groups for treatment: control, 5-FU, ivermectin (IVER), ivermectin plus 5-FU (IVER + 5-FU), 1A116, and 1A116 + 5-FU (N = 4 per treatment). The tumor size was measured biweekly with a caliper and volume estimated (time  =  0 indicates the beginning of treatment). (**B**) At the end of the experiment, animals were euthanized, and tumors of each group were removed and weighed. (**C**) The body weight of the animals was measured at the beginning and at the end of the treatments to evaluate signs of its toxicity. (**D**) Representative images of tumor and spleen for each treatment group. (**E**) Hematoxylin and eosin lung staining images showing representative micrometastases (indicated with yellow arrows) for the control, 5-FU, 1A116, and 1A116 + 5-FU groups. (**F**) Hematoxylin and eosin liver staining images showing representative micrometastases (indicated with yellow arrows). (**G**–**J**) Analysis of Rac1 inhibition on experimental metastasis development. After intrasplenic injection, tumors containing spleens (**G**) and livers (**H**) were collected and weight (**I**). Representative images of spleens (upper panel) and livers (lower panel). (**J**) Metastasis were counted under a magnifying glass. *, *p* ≤ 0.05; **, *p* ≤ 0.01; ***, *p* ≤ 0.001.

Once confirmed by in vitro assays, the possibility to reverse resistance to 5-FU we moved to an in vivo assay by injecting CT26^5FUR^ cells in BALB/c mice. For this experiment, we selected one drug in repositioning, ivermectin, and the Rac1 inhibitor 1A-116. Tumor growth kinetics indicated that 5-FU or ivermectin alone had no statistical effect on resistant CRC cells growing (Figure 6A,B). Surprisingly, Rac1 inhibitor 1A-116 administration statistically affected the growth evolution of tumor cells. Interestingly, both ivermectin and 1A-116 were able to induce sensitivity to 5-FU in resistant CT26^5FUR^ cells (Figure 6A,B and Figure S4A,B). No signs of toxicity were associated with treatments as evaluated by general animal behavior and weight (Figure 6C and Figure S4C) and the measurement of metabolic parameters (Figure S4D).

At the end of the experiment, we collected tumors, spleen, liver, and lungs for histological observation. We did not observe histological differences between tumors by H&E staining (Figure S5), but it was evident that animals treated with 1A-116 or a combination of 1A-116 with 5-FU did not develop splenomegaly (Figure 6D and Table 1). Microscopic visualization of histological sections indicated that combined treatment with 1A-116 and 5-FU reduced liver and lung metastasis development in mice (Figure 6E,F and Table 1), suggesting a role for Rac1 in CRC tumor growth, resistance, and metastatic dissemination. To further confirm the antimetastatic potential of Rac1 inhibition on 5-FU resistant CRC, we performed an intrasplenic cell injection, noting that 1A-116 treatment drastically reduced spleen tumor formation and resistant cell dissemination to the liver (Figure 6G–J).

### 3.6. Generation of Prognostic Signatures to Predict 5-FU-Based Therapies Resistance

**Figure 7:**
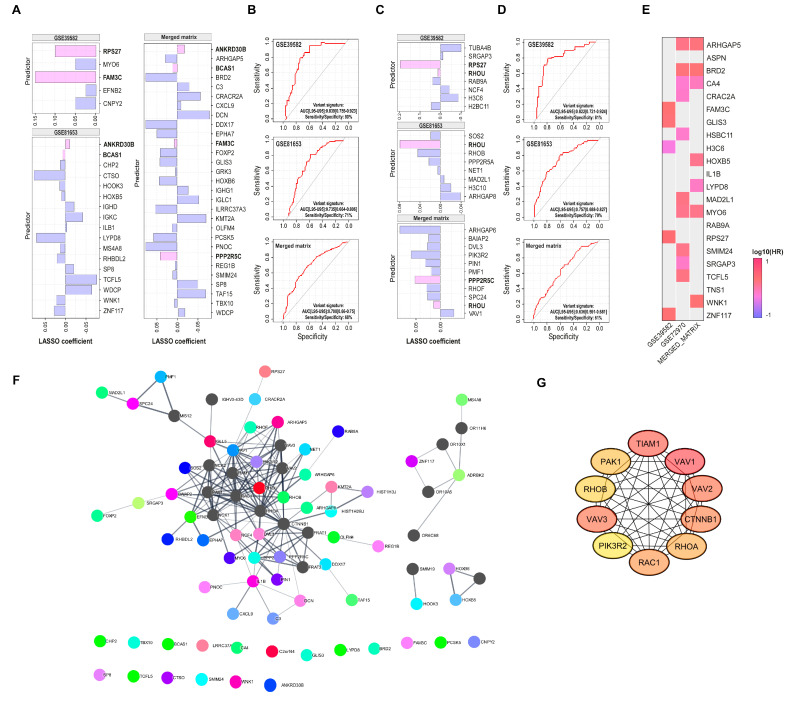
Generation of recurrence predictor signatures. (**A**) LASSO coefficient profiles showing the most important DEGs predictors of recurrence selected by LASSO regression analysis (left: DEGs predictors in data sets of patients treated with 5-FU monotherapy; right: DEGs predictors in pooled matrix of patients treated with 5-FU, FOLFIRI, or FOLFOX). (**B**) ROC curves showing the predictive efficiency of recurrence for each DEG signature on the test sub dataset (**C**) LASSO coefficient profiles showing the most important genes predictors of recurrence from the Reactome Signaling by Rho GTPases gene set selected by LASSO regression analysis in the 5-FU monotherapy datasets and combined treatments merged matrix. (**D**) ROC curves showing the predictive efficiency of recurrence for each DEG signature on the test sub dataset. In both (**A**) and (**C**), predictors that are common to more than one dataset or that appeared as predictors despite using different gene sets for the analysis are highlighted in light pink. (**E**) Heatmap showing the relationship between recurrence predictor gene expression and the patients’ overall survival. Patients were divided into groups with high and low mRNA predictor gene expression at median. Each heatmap cell corresponds to a predictor gene log_10_ HR (hazard ratio) for the respective dataset detailed in column name. Colors in the red range indicate HR > 0, while colors in the blue range indicate HR < 0. The graph only displays the predictor genes with significance level *p* < 0.05 in the log-rank test. (**F**) Expanded PPI network of the set of recurrence predictor genes (marked in color); the main common interactor proteins were obtained by Cytoscape StringApp and are shown in grey (selectivity of interactors = 0.5). (**G**) Top ten hub genes extracted from the predictors expanded PPI network; a greater red color intensity is associated with the highest number of connections in the network. AUC, area under the curve.

Finally, a LASSO regression was performed on our DEGs list to identify a gene signature predictive of recurrence for both 5-FU monotherapy and combined therapy datasets (Figure 7A). Given the observed importance of Rho GTPases in previous analyses, LASSO regression was also applied to 463 genes from the ‘Reactome Signaling by Rho GTPases’ gene set (REACTOME_SIGNALING_BY_RHO_GTPASES.v2024.1.Hs) to define a Rho GTPases-related signature in both datasets (Figure 7C). The predictive performance of the signatures was evaluated using ROC (Receiver Operating Characteristic) curve analysis (Figure 7B,D). While there was limited overlap in predictive genes between the different datasets, models fitted on monotherapy datasets exhibited better predictive performance, as seen in the ROC curve analysis. Notably, some of these predictive genes were associated with overall survival in CRC patients (Figure 7E).

To investigate the relationships among predictive genes linked to recurrence, a PPI network was constructed using the STRING database [44]. This network was then expanded to incorporate key interacting proteins associated with the input gene list, leading to the identification of the top ten hub genes (Figure 7F,G). These hub genes include the GTPases Rac1, RhoA, and RhoB; their guanine nucleotide exchange factors (GEFs) Vav1, Vav2, Vav3, and Tiam1; their effector Pak1; the phosphoinositide-3-kinase regulatory subunit PIK3R2; and β-catenin (CTNNB1). Despite the significant variability in the expression of these genes among patients, which hinders the identification of a consistent predictive signature, these findings collectively suggest a prominent role for Rho GTPases, particularly the Rac1 pathway, in modulating resistance to 5-FU-based therapies in CRC. This variability in gene expression likely reflects the heterogeneity of colorectal cancer, highlighting the need for personalized treatment approaches.

## 4. Discussion

CRC is the third most commonly diagnosed type of cancer worldwide. Early diagnosis, adenomas removal during screenings, and improved CRC treatments have reduced morbidity and mortality rates. However, CRC incidence is increasing in middle- and low-income countries, and early-onset CRC is also emerging, positioning CRC as a growing global public health challenge [60].

Since their discovery, 5-FU-based chemotherapies have been commonly used to treat CRC. However, resistance to treatment has greatly affected 5-FU clinical use. As CRC is a heterogeneous disease characterized by different genetic scenarios [61], a CRC molecular comprehensive characterization could shed light on the understanding of resistance mechanisms and improve therapies.

In this work, we addressed the identification of genes and pathways associated with the development of resistance to 5-FU and 5-FU-based treatments in CRC. Comparison of gene expression datasets revealed the enrichment of pathways previously reported to be associated with resistance, such as “TGFbeta signaling” and “SMAD2/3 activation by TGFbeta” [62,63,64]. TGFbeta is a cytokine involved both in physiological and pathological processes. Canonical signaling is mediated by SMAD transcription factors, but the TGFbeta “non-canonical” pathway involves activation of proteins such as MAPK, PI3K/AKT, and small GTPases as Rac1 and RhoA [65]. Categories and genes related to cell stemness [66,67], EGFR signaling [68,69], apoptosis [70], DNA repair [71], and interaction between tumor cells and the immune system [72] were also present in our analysis.

When we looked for potential compounds reversing 5-FU resistance-associated gene expression, we identified molecules belonging to different categories. Some of them were involved in modulation of hormonal messages (dopamine and serotonin receptor agonists and antagonists), selective estrogen receptor modulation (this category includes tamoxifen and raloxifene, widely used in breast cancer), and adrenergic receptor inhibition such as phentolamine. Additionally, we found anti-inflammatory compounds, mainly COX inhibitors. We focused on irinotecan, amitriptyline, and ivermectin, three drugs under repositioning, and selected ivermectin for our in vivo approach. As predicted by our analysis, ivermectin was able to re-sensitize CRC cells to 5-FU monotherapy, but no significant metastasis prevention was associated with this treatment. Noteworthy, the antitumor mechanism of action proposed for ivermectin is associated with the P21(Rac1)-Activated Kinase 1 (PAK1) function [73,74,75].

Interestingly, the most represented pathways in all datasets were directly or indirectly related to Rho GTPases activation. Indeed, activation of Rho GTPases may be under control of the Serum Response Factor as many upregulated genes in recurrent phenotypes contained serum response elements in their promoter sequences. Many studies have identified SRF as a central agent in the development of multiple types of cancer, which has classified it as a potential biomarker and therapeutic target, especially for cancers with a poor prognosis. SRF controls the expression of cytoskeleton, morphogenesis, and cell migration genes, but also SRF-MRTF complex activity also responds to Rho GTPase-induced actin changes, thereby coupling cytoskeletal gene expression to cytoskeletal dynamics [76,77,78]. Accordingly, it was recently described that active Rac1 modulates SRF/MRTF, which initiates a switch to a mesenchymal-like state characterized by therapy resistance in melanoma [79].

Rho-GTPases regulate a variety of important cellular activities, such as cytoskeletal remodeling, cell adhesion, cell movement, vesicle transport, angiogenesis, and cell cycle regulation [76,80,81,82,83]. Rho-GTPases are generally described as “molecular switches” because they fluctuate between their active conformation attached to GTP and the inactive conformation bound to GDP. The activation of the “molecular switch” is controlled by guanine nucleotide exchange factors (GEFs), which stimulate the release of GDP bound to the inactive form and promote combination with GTP [84]. The inactive state of Rho-GTPase is maintained by inhibitory molecules of the guanine nucleotide dissociation (GDIs) and GTPase activity activating proteins (GAPs) [84]. Through changes in Rho-GTPases protein levels, their activity, or their effector proteins, abnormal signaling could contribute to different steps of cancer progression, including proliferation, survival, invasion, and metastasis [22,85].

Rac1, RhoA, and Cdc42 are the three classical members of the Rho-GTPase family, with Rac1 being the one that has received increased attention [82]. Rac1 is widely expressed in tissues and is considered a regulatory factor related to cell movement and invasion. Rac1 is highly expressed and overactivated in many tumor types, and it has lately been related to resistance to therapy in several reports [79,86,87,88,89,90].

Based on our data and the literature, Rac1 could be engaging cellular mechanisms leading to 5-FU-based resistance in CRC. To confirm our hypothesis, we treated 5-FU-resistant CRC cells with Rac1 inhibitor 1A-116, noting that doses not compromising viability of CRC cells were sufficient to promote the reversal of morphological changes associated with resistance and re-sensitize resistant cells to 5-FU. Moreover, Rac1 inhibition restored the expression of epithelial markers in 5-FU-resistant cells, previously characterized in a mesenchymal-like state associated with therapy resistance. Administration of Rac1 inhibitors to mice bearing CRC tumors reduced tumor growth, sensitized resistant tumors to 5-FU monotherapy, and decreased metastasis development. Interestingly, tumors derived from animals treated with Rac1 inhibitor showed an increased number of immune cell infiltrates suggesting that Rac1 inhibition could be acting through different mechanisms, including immune escape mediated by tumor microenvironment [91].

Additionally, our data suggest that Rac1 inhibition could be an important strategy to overcome resistance to therapy in different cancer types. Indeed, Rac1-modulated pathways could be playing essential roles in developing resistance to different therapeutic approaches such as endocrine therapies, targeted therapies, and radiotherapy, as our enrichment analysis indicates common profiles between resistance to 5-FU-based therapies and gemcitabine and gefitinib in non-small cell lung cancer [92,93], dasatinib for breast, lung, and ovarian tumors [94], tamoxifen in estrogen receptor-positive breast cancer [95], and postradiation tumor escape of CRC [96].

Altogether, our data point to Rac1 as a potential target to overcome therapy resistance for CRC and other types of tumors and suggest that Rac1 inhibitor 1A-116 could represent a good therapeutic agent to overt CRC resistance for 5-FU-based therapies.

## 5. Conclusions

CRC is one of the third most diagnosed types of cancer worldwide and the second in terms of mortality. Therapies to treat CRC are often associated with the development of resistance, so initially responding tumors became resistant to treatment. Therapies based on 5-FU have been used in the clinics since 1950, but almost half of patients develop therapy resistance.

Our findings indicate that inhibition of Rac1 activation by the compound 1A-116 reverses 5-FU resistance in CRC cells, decreases tumor growth, and prevents metastasis development, suggesting a therapeutic application of 1A-116 for the treatment of therapy-resistant CRC. Further studies are needed to fully understand Rac1’s role in CRC progression and therapy resistance.

## Figures and Tables

**Table 1:** Analysis of metastasis. Mean of metastatic nodes detected per mouse by H&E in lung and liver. At the end of the experiment, the spleens of the mice were removed and measured with a caliper to detect signs of splenomegaly. The mean spleen lengths for each treatment are shown (N = 4).

Organ	Control	5-FU	Iver	Iver +5-FU	1A-116	1A-116 +5-FU
Lung (nodes/mouse)	2 ± 1	1.33 ± 1.15	1 ± 1	0.33 ± 0.58	0.67 ± 0.58	0 ± 0 *
Liver (nodes/mouse)	1.33 ± 0.58	1.33 ± 1.53	1.33 ± 1.53	1.33 ± 1.53	0.67 ± 0.58	0.67 ± 0.58
Splenomegaly ** (spleen length mm)	24.25 ± 2.06	25.75 ± 2.06	24.75 ± 1.50	26 ± 1.41	20.50 ± 1.91 *	23.25 ± 1.50

* *p* < 0.05 ordinary one-way ANOVA vs. control. ** Normal length reference: 15–20 mm.

## Data Availability

Data presented in this study are available upon request from the corresponding author.

## Supplementary Materials

The following supporting information can be downloaded at: https://www.mdpi.com/article/10.3390/cells13211776/s1, Figure S1: Dataset normalization and probe intensity filters; Figure S2: Cell viability assays; Figure S3: Rac1 activity inhibition in 5-FU resistant cells; Figure S4: Inhibition of Rac1 activity modulates in vivo CRC growth; Figure S5: Tumor histology; Figure S6: LASSO regression model; Table S1: 5-FU monotherapy detection platforms and sample sizes used in this study; Table S2: Detailed description of 5-FU monotherapy samples selected for analysis; Table S3: 5-FU-based therapies platforms and sample sizes used in this study; Table S4: Detailed description of 5-based therapies samples selected for analysis; Table S5: Available clinical data of patients treated with 5-FU monotherapy; Table S6: GSEA analysis between 5-FU treated recurrent and non-recurrent phenotypes for the GSE39582 dataset; Table S7: GSEA analysis between 5-FU treated recurrent and non-recurrent phenotypes for the GSE81653 dataset; Table S8: Common DEGs identified in patients with tumor recurrence after 5-FU therapy; Table S9: Available clinical data of patients treated with 5-FU-based therapies; Table S10: GSEA analysis between 5-FU, FOLFIRI, and FOLFLOX-treated recurrent and non-recurrent phenotypes for the merged matrix; Table S11: GSEA analysis between 5-FU, FOLFIRI, and FOLFLOX-treated recurrent and non-recurrent phenotypes for the 5000 more explicative genes selected by the RFE algorithm form a merged matrix; Table S12: Selected DEGs to perform CLUE Query (v.1.1) analysis; Table S13: CLUE Query (v.1.1) analysis for selected up-regulated and downregulated genes; Table S14: Perturbations CLUE Query (v.1.1) analysis for selected up-regulated and downregulated genes; Table S15: IC50 values of selected drugs were chosen from in silico studies on 5-FU sensitive and resistant cell lines.
