# Supplementary material for: Overcoming Therapy Resistance in Colorectal Cancer: Targeting the Rac1 Signaling Pathway as a Potential Therapeutic Approach
Source: Cells. 2024 Oct 26;13(21):1776. doi: 10.3390/cells13211776 (PMC11545287; doi:10.3390/cells13211776)
Supplement: Supplementary file 1 [file cells-13-01776-s001.zip › cells-3200276-supplementary.pdf]

SUPPLEMENTARY FIGURES:

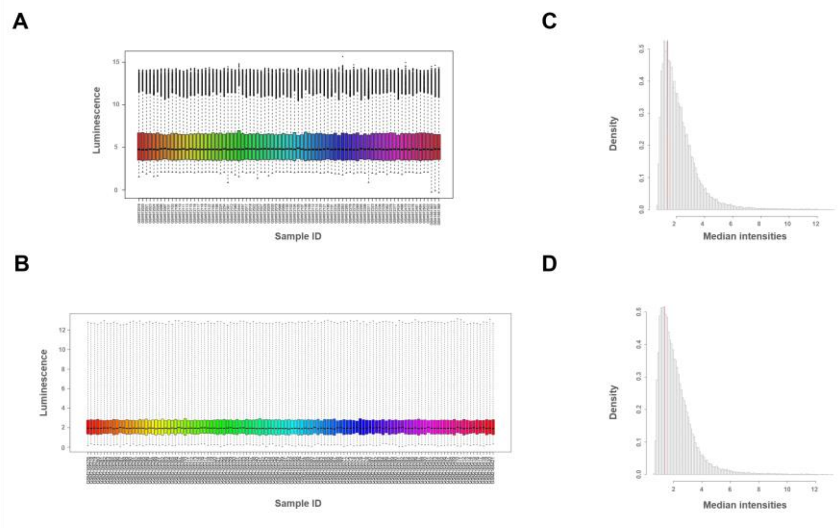

Figure S1. Dataset normalization and probes intensity filtering. (**A**,**B**) Distribution of log2 intensity values (mean and standard deviation) after normalization using the RMA method for series GSE39582 (A) and GSE81653 (B). (**C**,**D**) Histograms showing the median expression intensities for series GSE39582 (C) and GSE81653 (D). The red vertical line indicates the minimum expression threshold; probes located to the left of this line were discarded from the analysis.

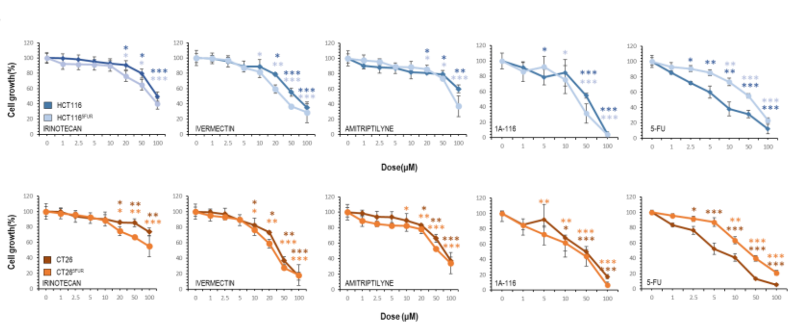

Figure S2. Cell viability assays. (**A-C**) Characterization of the effect of irinotecan, ivermectin, amitriptyline and 1A-116 on HCT116 and HCT116^{5FUR} (A) and CT26 and CT26^{5FUR} (B) cells viability. CRC cells were cultured in the presence of the indicated doses of drugs during 36 h. The number of living cells was estimated by tetrazolium salts reduction method (n = 3).

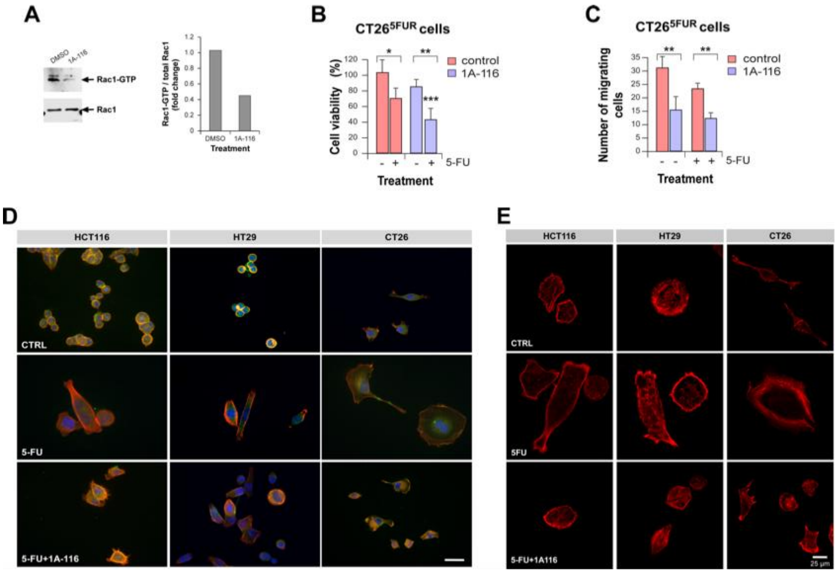

Figure S3. Rac1 activity inhibition in 5-FU resistant cells. (**A**) Inhibition of Rac1 activation in 5-FU resistant (CT26^{5FUR}) cells by 1A-116 treatment determined by pull-down assays. Levels of Rac1-GTP and total Rac1 were analyzed by Western blot (left panel) and quantified by ImageJ (right panel). (**B**) Reversion of resistance to 5-fluorouracil by treatment with the Rac1 inhibitor 1A-116 determined by viable cells counting after trypan blue staining. (**C**) Quantification of migrating 5-FU resistant CRC cells in the presence of 1A-116 by a wound-healing assay. (**D**) Differences in cell morphology between HCT116, HT29 and CT26 cells (upper row), HCT116^{5FUR}, HT29^{5FUR} and CT26^{5FUR} cells (middle row) and HCT116^{5FUR}, HT29^{5FUR}, CT26^{5FUR} cells treated with 20 µM 1A-116 for 36 h (lower row). Nuclear staining is shown in blue, tubulin in green and actin skeleton in red. Scale bar=50 µm. (**E**) Actin staining confocal images. Scale bar=25 µm.

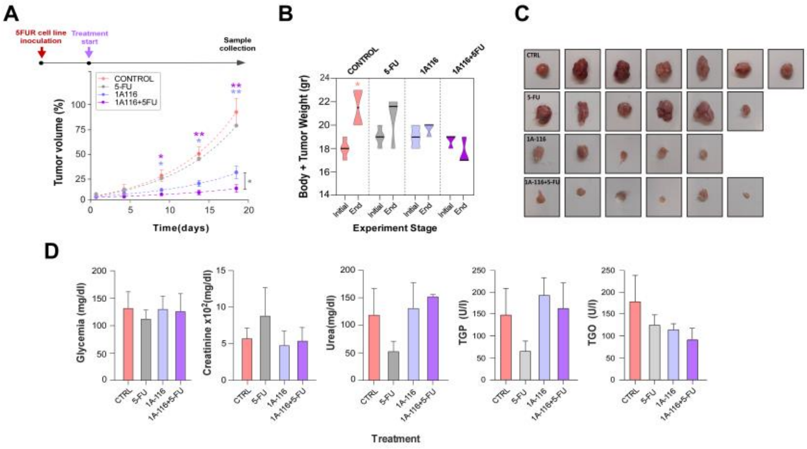

Figure S4. Inhibition of Rac1 activity modulates *in vivo* CRC growth. (**A**) BALB/c mice were subcutaneously challenged with CT26^{5FUR} cells. Ten days later tumors become evident and animals were randomly distributed in groups for treatment: control, 5-FU, 1A116 and 1A116+5FU (n = 6 per treatment). The tumor size was measured biweekly with a caliper and volume estimated (time = 0 indicates the beginning of treatment). (**B**) At the end of the experiment, animals were euthanized and tumors of each group were removed. (**C**) Body weight

of the animals was measured at the beginning and at the end of the treatments to evaluate signs of its toxicity. (**D**) Metabolic parameters were evaluated (GOT: glutamic-oxaloacetic transaminase; GPT: glutamic pyruvic transaminase).

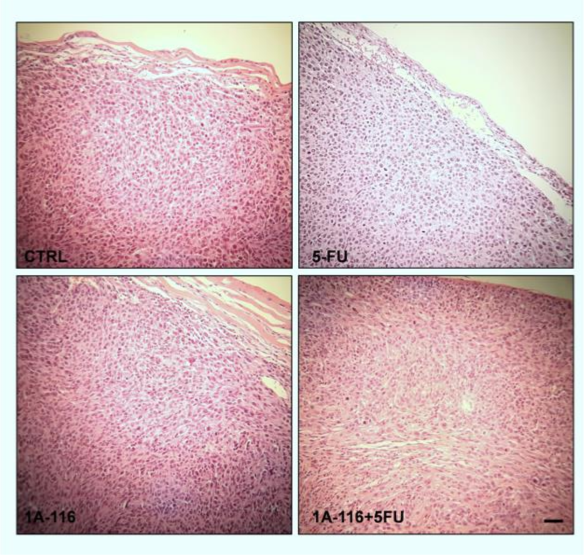

Figure S5. Tumor histology. Representative hematoxylin-eosin staining images of tumors at 20X magnification. Scale bar= 25 µm.

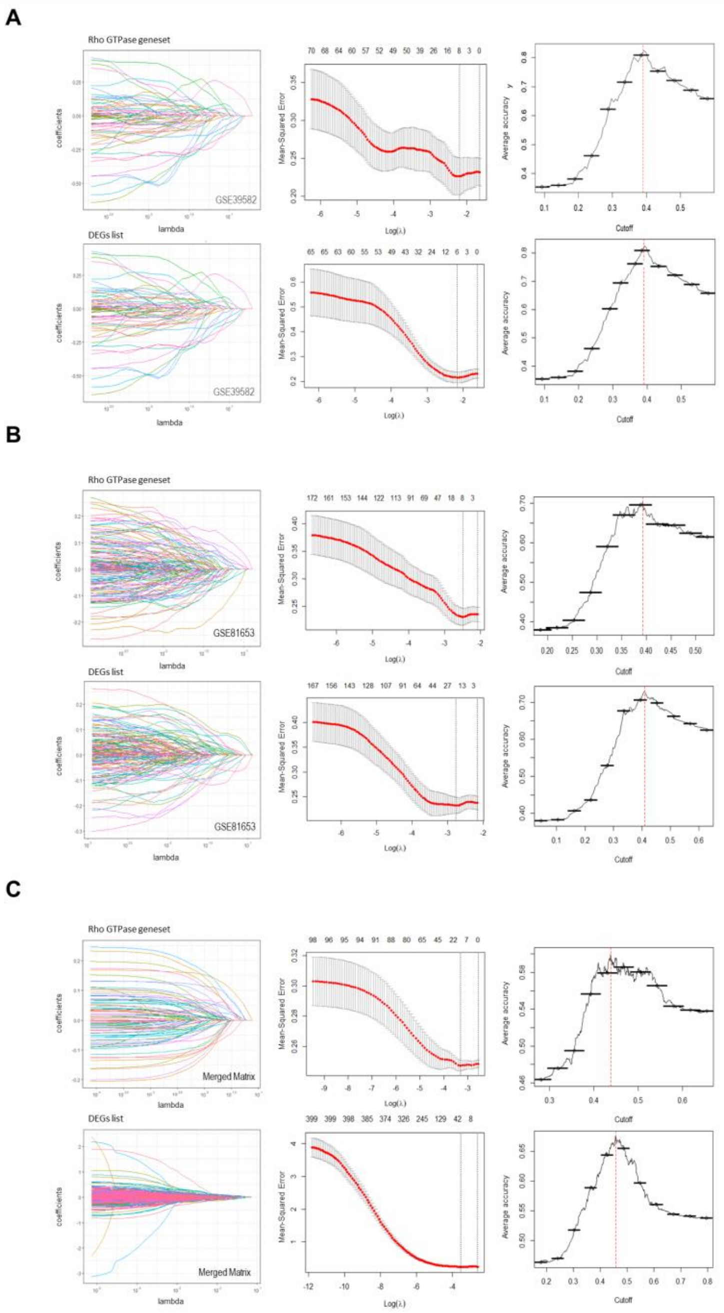

Figure S6. LASSO regression model. (**A**) GSE39582 LASSO regression model. Left: LASSO model coefficients as a function of regularization. Center: tenfold cross-validation for tuning parameter (lambda) selection, vertical lines were drawn at the optimal values by the minimum criteria and the 1 -SE criteria. Right: average accuracy vs. cutoff signature for ROC curve, vertical line indicates the optimal cutoff point with the highest average accuracy. Upper panel: Rho GTPases gene set, lower panel: DEGs list. (**B**) Same as A but for GSE81653 LASSO regression model. (**C**) Same as A, but for merged matrix LASSO regression model

SUPPLEMENTARY METHODS

Migration assay

$5x10^5$ $CT26^{5FUR}$ cells were seeded on culture-inserts for in vitro wound healing assays (ibidi, GmbH. Germany). 24 hours later, media was changed to DMEM supplemented with 1% FBS plus the corresponding treatment, and inserts were carefully removed. After 6 hours, pictures of the wound were taken and migrating cells were quantified (Nikon Ti2E fluorescent microscope).

SUPPLEMENTARY TABLES:

Table S1. 5-FU monotherapy detection platforms and sample sizes used in this study. Number of samples selected from series GSE81653 and GSE39582.

Plataform ID	Serie ID	Treatment	Selected samples	Recurrent	No recurrent
GPL570	GSE39582	5-FU	82	29	53
GPL16686	GSE81653	5-FU	192	73	119
	TOTAL		**274**	**102**	**172**

Table S2. Detailed description of 5-FU monotherapy samples selected for analysis. GEO codes for samples selected from GSE81653 and GSE39582 datasets.

plataform	serie.accesion	sample.accession	chemotherapy.type	recurrence.status
GPL570	GSE39582	GSM972017	5FU	NR
GPL570	GSE39582	GSM972018	5FU	NR
GPL570	GSE39582	GSM972019	5FU	R
GPL570	GSE39582	GSM972020	5FU	R
GPL570	GSE39582	GSM972021	5FU	R
GPL570	GSE39582	GSM972022	5FU	NR
GPL570	GSE39582	GSM972023	5FU	R
GPL570	GSE39582	GSM972024	5FU	R
GPL570	GSE39582	GSM972025	5FU	NR
GPL570	GSE39582	GSM972026	5FU	R
GPL570	GSE39582	GSM972027	5FU	NR
GPL570	GSE39582	GSM972028	5FU	NR
GPL570	GSE39582	GSM972029	5FU	NR
GPL570	GSE39582	GSM972030	5FU	NR
GPL570	GSE39582	GSM972079	5FU	NR
GPL570	GSE39582	GSM972096	5FU	R
GPL570	GSE39582	GSM972097	5FU	R
GPL570	GSE39582	GSM972098	5FU	NR
GPL570	GSE39582	GSM972099	5FU	NR
GPL570	GSE39582	GSM972100	5FU	NR
GPL570	GSE39582	GSM972101	5FU	R
GPL570	GSE39582	GSM972102	5FU	NR
GPL570	GSE39582	GSM972103	5FU	R
GPL570	GSE39582	GSM972104	5FU	NR
GPL570	GSE39582	GSM972105	5FU	NR
GPL570	GSE39582	GSM972106	5FU	R
GPL570	GSE39582	GSM972107	5FU	NR
GPL570	GSE39582	GSM972108	5FU	NR
GPL570	GSE39582	GSM972109	5FU	NR
GPL570	GSE39582	GSM972110	5FU	R
GPL570	GSE39582	GSM972111	5FU	R
GPL570	GSE39582	GSM972112	5FU	NR
GPL570	GSE39582	GSM972113	5FU	R
GPL570	GSE39582	GSM972114	5FU	NR
GPL570	GSE39582	GSM972115	5FU	R
GPL570	GSE39582	GSM972116	5FU	R
GPL570	GSE39582	GSM972117	5FU	R
GPL570	GSE39582	GSM972118	5FU	R

plataform	serie.accesion	sample.accession	chemotherapy.type	recurrence.status
GPL570	GSE39582	GSM972119	5FU	R
GPL570	GSE39582	GSM972120	5FU	NR
GPL570	GSE39582	GSM972121	5FU	NR
GPL570	GSE39582	GSM972122	5FU	R
GPL570	GSE39582	GSM972164	5FU	R
GPL570	GSE39582	GSM972166	5FU	R
GPL570	GSE39582	GSM972190	5FU	NR
GPL570	GSE39582	GSM972191	5FU	NR
GPL570	GSE39582	GSM972224	5FU	R
GPL570	GSE39582	GSM972234	5FU	NR
GPL570	GSE39582	GSM972254	5FU	R
GPL570	GSE39582	GSM972259	5FU	NR
GPL570	GSE39582	GSM972263	5FU	NR
GPL570	GSE39582	GSM972265	5FU	NR
GPL570	GSE39582	GSM972276	5FU	NR
GPL570	GSE39582	GSM972279	5FU	NR
GPL570	GSE39582	GSM972289	5FU	NR
GPL570	GSE39582	GSM972295	5FU	NR
GPL570	GSE39582	GSM972306	5FU	NR
GPL570	GSE39582	GSM972311	5FU	NR
GPL570	GSE39582	GSM972323	5FU	NR
GPL570	GSE39582	GSM972334	5FU	NR
GPL570	GSE39582	GSM972336	5FU	NR
GPL570	GSE39582	GSM972339	5FU	NR
GPL570	GSE39582	GSM972361	5FU	R
GPL570	GSE39582	GSM972362	5FU	NR
GPL570	GSE39582	GSM972372	5FU	R
GPL570	GSE39582	GSM972376	5FU	NR
GPL570	GSE39582	GSM972377	5FU	NR
GPL570	GSE39582	GSM972380	5FU	R
GPL570	GSE39582	GSM972424	5FU	NR
GPL570	GSE39582	GSM972444	5FU	R
GPL570	GSE39582	GSM972457	5FU	R
GPL570	GSE39582	GSM972466	5FU	NR
GPL570	GSE39582	GSM972470	5FU	NR
GPL570	GSE39582	GSM972472	5FU	NR
GPL570	GSE39582	GSM972476	5FU	NR
GPL570	GSE39582	GSM972481	5FU	NR
GPL570	GSE39582	GSM972484	5FU	NR
GPL570	GSE39582	GSM972503	5FU	NR
GPL570	GSE39582	GSM972522	5FU	NR

plataform	serie.accesion	sample.accession	chemotherapy.type	recurrence.status
GPL570	GSE39582	GSM1681363	5FU	NR
GPL570	GSE39582	GSM1681367	5FU	NR
GPL570	GSE39582	GSM1681369	5FU	NR
GPL16686	GSE81653	GSM2165276	5-FU	R
GPL16686	GSE81653	GSM2165277	5-FU	R
GPL16686	GSE81653	GSM2165278	5-FU	R
GPL16686	GSE81653	GSM2165279	5-FU	R
GPL16686	GSE81653	GSM2165280	5-FU	R
GPL16686	GSE81653	GSM2165281	5-FU	R
GPL16686	GSE81653	GSM2165282	5-FU	R
GPL16686	GSE81653	GSM2165283	5-FU	R
GPL16686	GSE81653	GSM2165284	5-FU	R
GPL16686	GSE81653	GSM2165285	5-FU	R
GPL16686	GSE81653	GSM2165286	5-FU	R
GPL16686	GSE81653	GSM2165287	5-FU	R
GPL16686	GSE81653	GSM2165288	5-FU	R
GPL16686	GSE81653	GSM2165289	5-FU	R
GPL16686	GSE81653	GSM2165290	5-FU	R
GPL16686	GSE81653	GSM2165291	5-FU	R
GPL16686	GSE81653	GSM2165292	5-FU	R
GPL16686	GSE81653	GSM2165293	5-FU	R
GPL16686	GSE81653	GSM2165294	5-FU	R
GPL16686	GSE81653	GSM2165295	5-FU	R
GPL16686	GSE81653	GSM2165296	5-FU	R
GPL16686	GSE81653	GSM2165297	5-FU	R
GPL16686	GSE81653	GSM2165298	5-FU	R
GPL16686	GSE81653	GSM2165299	5-FU	R
GPL16686	GSE81653	GSM2165300	5-FU	R
GPL16686	GSE81653	GSM2165301	5-FU	R
GPL16686	GSE81653	GSM2165302	5-FU	R
GPL16686	GSE81653	GSM2165303	5-FU	R
GPL16686	GSE81653	GSM2165304	5-FU	R
GPL16686	GSE81653	GSM2165305	5-FU	R
GPL16686	GSE81653	GSM2165306	5-FU	R
GPL16686	GSE81653	GSM2165307	5-FU	R
GPL16686	GSE81653	GSM2165308	5-FU	R
GPL16686	GSE81653	GSM2165309	5-FU	R
GPL16686	GSE81653	GSM2165310	5-FU	R
GPL16686	GSE81653	GSM2165311	5-FU	R
GPL16686	GSE81653	GSM2165312	5-FU	R
GPL16686	GSE81653	GSM2165313	5-FU	R

plataform	serie.accesion	sample.accession	chemotherapy.type	recurrence.status
GPL16686	GSE81653	GSM2165314	5-FU	R
GPL16686	GSE81653	GSM2165315	5-FU	R
GPL16686	GSE81653	GSM2165316	5-FU	R
GPL16686	GSE81653	GSM2165317	5-FU	R
GPL16686	GSE81653	GSM2165318	5-FU	R
GPL16686	GSE81653	GSM2165319	5-FU	R
GPL16686	GSE81653	GSM2165320	5-FU	R
GPL16686	GSE81653	GSM2165321	5-FU	R
GPL16686	GSE81653	GSM2165322	5-FU	R
GPL16686	GSE81653	GSM2165323	5-FU	R
GPL16686	GSE81653	GSM2165324	5-FU	R
GPL16686	GSE81653	GSM2165325	5-FU	R
GPL16686	GSE81653	GSM2165326	5-FU	R
GPL16686	GSE81653	GSM2165327	5-FU	R
GPL16686	GSE81653	GSM2165328	5-FU	R
GPL16686	GSE81653	GSM2165329	5-FU	R
GPL16686	GSE81653	GSM2165330	5-FU	R
GPL16686	GSE81653	GSM2165331	5-FU	R
GPL16686	GSE81653	GSM2165332	5-FU	R
GPL16686	GSE81653	GSM2165333	5-FU	R
GPL16686	GSE81653	GSM2165334	5-FU	R
GPL16686	GSE81653	GSM2165335	5-FU	R
GPL16686	GSE81653	GSM2165336	5-FU	R
GPL16686	GSE81653	GSM2165337	5-FU	R
GPL16686	GSE81653	GSM2165338	5-FU	R
GPL16686	GSE81653	GSM2165339	5-FU	R
GPL16686	GSE81653	GSM2165340	5-FU	R
GPL16686	GSE81653	GSM2165341	5-FU	R
GPL16686	GSE81653	GSM2165342	5-FU	R
GPL16686	GSE81653	GSM2165343	5-FU	R
GPL16686	GSE81653	GSM2165344	5-FU	R
GPL16686	GSE81653	GSM2165345	5-FU	R
GPL16686	GSE81653	GSM2165346	5-FU	R
GPL16686	GSE81653	GSM2165347	5-FU	R
GPL16686	GSE81653	GSM2165348	5-FU	R
GPL16686	GSE81653	GSM2165349	5-FU	NR
GPL16686	GSE81653	GSM2165350	5-FU	NR
GPL16686	GSE81653	GSM2165351	5-FU	NR
GPL16686	GSE81653	GSM2165352	5-FU	NR
GPL16686	GSE81653	GSM2165353	5-FU	NR
GPL16686	GSE81653	GSM2165354	5-FU	NR

plataform	serie.accesion	sample.accession	chemotherapy.type	recurrence.status
GPL16686	GSE81653	GSM2165355	5-FU	NR
GPL16686	GSE81653	GSM2165356	5-FU	NR
GPL16686	GSE81653	GSM2165357	5-FU	NR
GPL16686	GSE81653	GSM2165358	5-FU	NR
GPL16686	GSE81653	GSM2165359	5-FU	NR
GPL16686	GSE81653	GSM2165360	5-FU	NR
GPL16686	GSE81653	GSM2165361	5-FU	NR
GPL16686	GSE81653	GSM2165362	5-FU	NR
GPL16686	GSE81653	GSM2165363	5-FU	NR
GPL16686	GSE81653	GSM2165364	5-FU	NR
GPL16686	GSE81653	GSM2165365	5-FU	NR
GPL16686	GSE81653	GSM2165366	5-FU	NR
GPL16686	GSE81653	GSM2165367	5-FU	NR
GPL16686	GSE81653	GSM2165368	5-FU	NR
GPL16686	GSE81653	GSM2165369	5-FU	NR
GPL16686	GSE81653	GSM2165370	5-FU	NR
GPL16686	GSE81653	GSM2165371	5-FU	NR
GPL16686	GSE81653	GSM2165372	5-FU	NR
GPL16686	GSE81653	GSM2165373	5-FU	NR
GPL16686	GSE81653	GSM2165374	5-FU	NR
GPL16686	GSE81653	GSM2165375	5-FU	NR
GPL16686	GSE81653	GSM2165376	5-FU	NR
GPL16686	GSE81653	GSM2165377	5-FU	NR
GPL16686	GSE81653	GSM2165378	5-FU	NR
GPL16686	GSE81653	GSM2165379	5-FU	NR
GPL16686	GSE81653	GSM2165380	5-FU	NR
GPL16686	GSE81653	GSM2165381	5-FU	NR
GPL16686	GSE81653	GSM2165382	5-FU	NR
GPL16686	GSE81653	GSM2165383	5-FU	NR
GPL16686	GSE81653	GSM2165384	5-FU	NR
GPL16686	GSE81653	GSM2165385	5-FU	NR
GPL16686	GSE81653	GSM2165386	5-FU	NR
GPL16686	GSE81653	GSM2165387	5-FU	NR
GPL16686	GSE81653	GSM2165388	5-FU	NR
GPL16686	GSE81653	GSM2165389	5-FU	NR
GPL16686	GSE81653	GSM2165390	5-FU	NR
GPL16686	GSE81653	GSM2165391	5-FU	NR
GPL16686	GSE81653	GSM2165392	5-FU	NR
GPL16686	GSE81653	GSM2165393	5-FU	NR
GPL16686	GSE81653	GSM2165394	5-FU	NR
GPL16686	GSE81653	GSM2165395	5-FU	NR

plataform	serie.accesion	sample.accession	chemotherapy.type	recurrence.status
GPL16686	GSE81653	GSM2165396	5-FU	NR
GPL16686	GSE81653	GSM2165397	5-FU	NR
GPL16686	GSE81653	GSM2165398	5-FU	NR
GPL16686	GSE81653	GSM2165399	5-FU	NR
GPL16686	GSE81653	GSM2165400	5-FU	NR
GPL16686	GSE81653	GSM2165401	5-FU	NR
GPL16686	GSE81653	GSM2165402	5-FU	NR
GPL16686	GSE81653	GSM2165403	5-FU	NR
GPL16686	GSE81653	GSM2165404	5-FU	NR
GPL16686	GSE81653	GSM2165405	5-FU	NR
GPL16686	GSE81653	GSM2165406	5-FU	NR
GPL16686	GSE81653	GSM2165407	5-FU	NR
GPL16686	GSE81653	GSM2165408	5-FU	NR
GPL16686	GSE81653	GSM2165409	5-FU	NR
GPL16686	GSE81653	GSM2165410	5-FU	NR
GPL16686	GSE81653	GSM2165411	5-FU	NR
GPL16686	GSE81653	GSM2165412	5-FU	NR
GPL16686	GSE81653	GSM2165413	5-FU	NR
GPL16686	GSE81653	GSM2165414	5-FU	NR
GPL16686	GSE81653	GSM2165415	5-FU	NR
GPL16686	GSE81653	GSM2165416	5-FU	NR
GPL16686	GSE81653	GSM2165417	5-FU	NR
GPL16686	GSE81653	GSM2165418	5-FU	NR
GPL16686	GSE81653	GSM2165419	5-FU	NR
GPL16686	GSE81653	GSM2165420	5-FU	NR
GPL16686	GSE81653	GSM2165421	5-FU	NR
GPL16686	GSE81653	GSM2165422	5-FU	NR
GPL16686	GSE81653	GSM2165423	5-FU	NR
GPL16686	GSE81653	GSM2165424	5-FU	NR
GPL16686	GSE81653	GSM2165425	5-FU	NR
GPL16686	GSE81653	GSM2165426	5-FU	NR
GPL16686	GSE81653	GSM2165427	5-FU	NR
GPL16686	GSE81653	GSM2165428	5-FU	NR
GPL16686	GSE81653	GSM2165429	5-FU	NR
GPL16686	GSE81653	GSM2165430	5-FU	NR
GPL16686	GSE81653	GSM2165431	5-FU	NR
GPL16686	GSE81653	GSM2165432	5-FU	NR
GPL16686	GSE81653	GSM2165433	5-FU	NR
GPL16686	GSE81653	GSM2165434	5-FU	NR
GPL16686	GSE81653	GSM2165435	5-FU	NR
GPL16686	GSE81653	GSM2165436	5-FU	NR

plataform	serie.accesion	sample.accession	chemotherapy.type	recurrence.status
GPL16686	GSE81653	GSM2165437	5-FU	NR
GPL16686	GSE81653	GSM2165438	5-FU	NR
GPL16686	GSE81653	GSM2165439	5-FU	NR
GPL16686	GSE81653	GSM2165440	5-FU	NR
GPL16686	GSE81653	GSM2165441	5-FU	NR
GPL16686	GSE81653	GSM2165442	5-FU	NR
GPL16686	GSE81653	GSM2165443	5-FU	NR
GPL16686	GSE81653	GSM2165444	5-FU	NR
GPL16686	GSE81653	GSM2165445	5-FU	NR
GPL16686	GSE81653	GSM2165446	5-FU	NR
GPL16686	GSE81653	GSM2165447	5-FU	NR
GPL16686	GSE81653	GSM2165448	5-FU	NR
GPL16686	GSE81653	GSM2165449	5-FU	NR
GPL16686	GSE81653	GSM2165450	5-FU	NR
GPL16686	GSE81653	GSM2165451	5-FU	NR
GPL16686	GSE81653	GSM2165452	5-FU	NR
GPL16686	GSE81653	GSM2165453	5-FU	NR
GPL16686	GSE81653	GSM2165454	5-FU	NR
GPL16686	GSE81653	GSM2165455	5-FU	NR
GPL16686	GSE81653	GSM2165456	5-FU	NR
GPL16686	GSE81653	GSM2165457	5-FU	NR
GPL16686	GSE81653	GSM2165458	5-FU	NR
GPL16686	GSE81653	GSM2165459	5-FU	NR
GPL16686	GSE81653	GSM2165460	5-FU	NR
GPL16686	GSE81653	GSM2165461	5-FU	NR
GPL16686	GSE81653	GSM2165462	5-FU	NR
GPL16686	GSE81653	GSM2165463	5-FU	NR
GPL16686	GSE81653	GSM2165464	5-FU	NR
GPL16686	GSE81653	GSM2165465	5-FU	NR
GPL16686	GSE81653	GSM2165466	5-FU	NR
GPL16686	GSE81653	GSM2165467	5-FU	NR

Table S3. 5-FU-based therapies platforms and sample sizes used in this study. Number of selected samples from GSE81653, GSE39582, and GSE72970 for the construction of the merged expression matrix.

PlataformID	SerieID	Treatment	Selected samples	Recurrent	No recurrent	Total serie samples
GPL16686	GSE81653	5-FU	192	73	119	358
		FOLFOX	166	102	64	
GPL570	GSE39582	5-FU	82	29	53	117
		FOLFIRI	12	3	9	
		FOLFOX	23	11	12	
	GSE72970	FOLFIRI	60	33	27	92
		FOLFOX	32	12	20	
			**TOTAL**	**263**	**304**	**567**

Table S4. Detailed description of 5-based therapies samples selected for analysis. GEO codes from samples selected from GSE81653, GSE39582 and GSE72970 datasets for merged matrix construction.

plataform	serie.accesion	sample.accession	chemotherapy.type	recurrence.status
GPL570	GSE39582	GSM972045	FOLFIRI	NR
GPL570	GSE39582	GSM972252	FOLFIRI	NR
GPL570	GSE39582	GSM972253	FOLFIRI	NR
GPL570	GSE39582	GSM972272	FOLFIRI	NR
GPL570	GSE39582	GSM972284	FOLFIRI	NR
GPL570	GSE39582	GSM972288	FOLFIRI	NR
GPL570	GSE39582	GSM972299	FOLFIRI	NR
GPL570	GSE39582	GSM972371	FOLFIRI	NR
GPL570	GSE39582	GSM972467	FOLFIRI	NR
GPL570	GSE39582	GSM1681368	FOLFIRI	NR
GPL570	GSE72970	GSM1875901	FOLFIRI	NR
GPL570	GSE72970	GSM1875904	FOLFIRI	NR
GPL570	GSE72970	GSM1875909	FOLFIRI	NR
GPL570	GSE72970	GSM1875911	FOLFIRI	NR
GPL570	GSE72970	GSM1875912	FOLFIRI	NR
GPL570	GSE72970	GSM1875915	FOLFIRI	NR
GPL570	GSE72970	GSM1875931	FOLFIRI	NR
GPL570	GSE72970	GSM1875933	FOLFIRI	NR
GPL570	GSE72970	GSM1875936	FOLFIRI	NR
GPL570	GSE72970	GSM1875940	FOLFIRI	NR
GPL570	GSE72970	GSM1875941	FOLFIRI	NR
GPL570	GSE72970	GSM1875942	FOLFIRI	NR
GPL570	GSE72970	GSM1875943	FOLFIRI	NR

plataform	serie.accesion	sample.accession	chemotherapy.type	recurrence.status
GPL570	GSE72970	GSM1875945	FOLFIRI	NR
GPL570	GSE72970	GSM1875946	FOLFIRI	NR
GPL570	GSE72970	GSM1875950	FOLFIRI	NR
GPL570	GSE72970	GSM1875957	FOLFIRI	NR
GPL570	GSE72970	GSM1875958	FOLFIRI	NR
GPL570	GSE72970	GSM1875962	FOLFIRI	NR
GPL570	GSE72970	GSM1875963	FOLFIRI	NR
GPL570	GSE72970	GSM1875966	FOLFIRI	NR
GPL570	GSE72970	GSM1875971	FOLFIRI	NR
GPL570	GSE72970	GSM1875980	FOLFIRI	NR
GPL570	GSE72970	GSM1875991	FOLFIRI	NR
GPL570	GSE72970	GSM1876011	FOLFIRI	NR
GPL570	GSE72970	GSM1876012	FOLFIRI	NR
GPL570	GSE72970	GSM1876013	FOLFIRI	NR
GPL570	GSE39582	GSM972064	FOLFIRI	R
GPL570	GSE39582	GSM972402	FOLFIRI	R
GPL570	GSE72970	GSM1875903	FOLFIRI	R
GPL570	GSE72970	GSM1875905	FOLFIRI	R
GPL570	GSE72970	GSM1875906	FOLFIRI	R
GPL570	GSE72970	GSM1875908	FOLFIRI	R
GPL570	GSE72970	GSM1875910	FOLFIRI	R
GPL570	GSE72970	GSM1875913	FOLFIRI	R
GPL570	GSE72970	GSM1875921	FOLFIRI	R
GPL570	GSE72970	GSM1875922	FOLFIRI	R
GPL570	GSE72970	GSM1875925	FOLFIRI	R
GPL570	GSE72970	GSM1875926	FOLFIRI	R
GPL570	GSE72970	GSM1875927	FOLFIRI	R
GPL570	GSE72970	GSM1875928	FOLFIRI	R
GPL570	GSE72970	GSM1875930	FOLFIRI	R
GPL570	GSE72970	GSM1875934	FOLFIRI	R
GPL570	GSE72970	GSM1875939	FOLFIRI	R
GPL570	GSE72970	GSM1875944	FOLFIRI	R
GPL570	GSE72970	GSM1875949	FOLFIRI	R
GPL570	GSE72970	GSM1875951	FOLFIRI	R
GPL570	GSE72970	GSM1875953	FOLFIRI	R
GPL570	GSE72970	GSM1875960	FOLFIRI	R
GPL570	GSE72970	GSM1875961	FOLFIRI	R
GPL570	GSE72970	GSM1875964	FOLFIRI	R
GPL570	GSE72970	GSM1875965	FOLFIRI	R
GPL570	GSE72970	GSM1875967	FOLFIRI	R
GPL570	GSE72970	GSM1875968	FOLFIRI	R
GPL570	GSE72970	GSM1875973	FOLFIRI	R

plataform	serie.accesion	sample.accession	chemotherapy.type	recurrence.status
GPL570	GSE72970	GSM1875974	FOLFIRI	R
GPL570	GSE72970	GSM1875976	FOLFIRI	R
GPL570	GSE72970	GSM1875977	FOLFIRI	R
GPL570	GSE72970	GSM1875978	FOLFIRI	R
GPL570	GSE72970	GSM1875983	FOLFIRI	R
GPL570	GSE72970	GSM1875993	FOLFIRI	R
GPL570	GSE72970	GSM1875998	FOLFIRI	R
GPL570	GSE39582	GSM972128	FOLFOX	NR
GPL570	GSE39582	GSM972227	FOLFOX	NR
GPL570	GSE39582	GSM972230	FOLFOX	NR
GPL570	GSE39582	GSM972231	FOLFOX	NR
GPL570	GSE39582	GSM972232	FOLFOX	NR
GPL570	GSE39582	GSM972235	FOLFOX	NR
GPL570	GSE39582	GSM972236	FOLFOX	NR
GPL570	GSE39582	GSM972238	FOLFOX	NR
GPL570	GSE39582	GSM972240	FOLFOX	NR
GPL570	GSE39582	GSM972250	FOLFOX	NR
GPL570	GSE39582	GSM972260	FOLFOX	NR
GPL570	GSE39582	GSM972266	FOLFOX	NR
GPL570	GSE72970	GSM1875897	FOLFOX	NR
GPL570	GSE72970	GSM1875898	FOLFOX	NR
GPL570	GSE72970	GSM1875900	FOLFOX	NR
GPL570	GSE72970	GSM1875902	FOLFOX	NR
GPL570	GSE72970	GSM1875914	FOLFOX	NR
GPL570	GSE72970	GSM1875916	FOLFOX	NR
GPL570	GSE72970	GSM1875918	FOLFOX	NR
GPL570	GSE72970	GSM1875919	FOLFOX	NR
GPL570	GSE72970	GSM1875920	FOLFOX	NR
GPL570	GSE72970	GSM1875923	FOLFOX	NR
GPL570	GSE72970	GSM1875924	FOLFOX	NR
GPL570	GSE72970	GSM1875929	FOLFOX	NR
GPL570	GSE72970	GSM1875932	FOLFOX	NR
GPL570	GSE72970	GSM1875948	FOLFOX	NR
GPL570	GSE72970	GSM1875954	FOLFOX	NR
GPL570	GSE72970	GSM1875955	FOLFOX	NR
GPL570	GSE72970	GSM1875956	FOLFOX	NR
GPL570	GSE72970	GSM1875969	FOLFOX	NR
GPL570	GSE72970	GSM1875972	FOLFOX	NR
GPL570	GSE72970	GSM1875981	FOLFOX	NR
GPL16686	GSE81653	GSM2165532	FOLFOX	NR
GPL16686	GSE81653	GSM2165533	FOLFOX	NR
GPL16686	GSE81653	GSM2165534	FOLFOX	NR

plataform	serie.accesion	sample.accession	chemotherapy.type	recurrence.status
GPL16686	GSE81653	GSM2165535	FOLFOX	NR
GPL16686	GSE81653	GSM2165536	FOLFOX	NR
GPL16686	GSE81653	GSM2165537	FOLFOX	NR
GPL16686	GSE81653	GSM2165538	FOLFOX	NR
GPL16686	GSE81653	GSM2165539	FOLFOX	NR
GPL16686	GSE81653	GSM2165540	FOLFOX	NR
GPL16686	GSE81653	GSM2165541	FOLFOX	NR
GPL16686	GSE81653	GSM2165542	FOLFOX	NR
GPL16686	GSE81653	GSM2165543	FOLFOX	NR
GPL16686	GSE81653	GSM2165544	FOLFOX	NR
GPL16686	GSE81653	GSM2165545	FOLFOX	NR
GPL16686	GSE81653	GSM2165546	FOLFOX	NR
GPL16686	GSE81653	GSM2165547	FOLFOX	NR
GPL16686	GSE81653	GSM2165548	FOLFOX	NR
GPL16686	GSE81653	GSM2165549	FOLFOX	NR
GPL16686	GSE81653	GSM2165550	FOLFOX	NR
GPL16686	GSE81653	GSM2165551	FOLFOX	NR
GPL16686	GSE81653	GSM2165552	FOLFOX	NR
GPL16686	GSE81653	GSM2165553	FOLFOX	NR
GPL16686	GSE81653	GSM2165554	FOLFOX	NR
GPL16686	GSE81653	GSM2165555	FOLFOX	NR
GPL16686	GSE81653	GSM2165556	FOLFOX	NR
GPL16686	GSE81653	GSM2165557	FOLFOX	NR
GPL16686	GSE81653	GSM2165558	FOLFOX	NR
GPL16686	GSE81653	GSM2165559	FOLFOX	NR
GPL16686	GSE81653	GSM2165560	FOLFOX	NR
GPL16686	GSE81653	GSM2165561	FOLFOX	NR
GPL16686	GSE81653	GSM2165562	FOLFOX	NR
GPL16686	GSE81653	GSM2165563	FOLFOX	NR
GPL16686	GSE81653	GSM2165564	FOLFOX	NR
GPL16686	GSE81653	GSM2165565	FOLFOX	NR
GPL16686	GSE81653	GSM2165566	FOLFOX	NR
GPL16686	GSE81653	GSM2165567	FOLFOX	NR
GPL16686	GSE81653	GSM2165568	FOLFOX	NR
GPL16686	GSE81653	GSM2165569	FOLFOX	NR
GPL16686	GSE81653	GSM2165570	FOLFOX	NR
GPL16686	GSE81653	GSM2165571	FOLFOX	NR
GPL16686	GSE81653	GSM2165572	FOLFOX	NR
GPL16686	GSE81653	GSM2165573	FOLFOX	NR
GPL16686	GSE81653	GSM2165574	FOLFOX	NR
GPL16686	GSE81653	GSM2165575	FOLFOX	NR
GPL16686	GSE81653	GSM2165576	FOLFOX	NR

plataform	serie.accesion	sample.accession	chemotherapy.type	recurrence.status
GPL16686	GSE81653	GSM2165577	FOLFOX	NR
GPL16686	GSE81653	GSM2165578	FOLFOX	NR
GPL16686	GSE81653	GSM2165579	FOLFOX	NR
GPL16686	GSE81653	GSM2165580	FOLFOX	NR
GPL16686	GSE81653	GSM2165581	FOLFOX	NR
GPL16686	GSE81653	GSM2165582	FOLFOX	NR
GPL16686	GSE81653	GSM2165583	FOLFOX	NR
GPL16686	GSE81653	GSM2165584	FOLFOX	NR
GPL16686	GSE81653	GSM2165585	FOLFOX	NR
GPL16686	GSE81653	GSM2165586	FOLFOX	NR
GPL16686	GSE81653	GSM2165587	FOLFOX	NR
GPL16686	GSE81653	GSM2165588	FOLFOX	NR
GPL16686	GSE81653	GSM2165589	FOLFOX	NR
GPL16686	GSE81653	GSM2165590	FOLFOX	NR
GPL16686	GSE81653	GSM2165591	FOLFOX	NR
GPL16686	GSE81653	GSM2165592	FOLFOX	NR
GPL16686	GSE81653	GSM2165593	FOLFOX	NR
GPL16686	GSE81653	GSM2165594	FOLFOX	NR
GPL16686	GSE81653	GSM2165595	FOLFOX	NR
GPL16686	GSE81653	GSM2165596	FOLFOX	NR
GPL16686	GSE81653	GSM2165597	FOLFOX	NR
GPL16686	GSE81653	GSM2165598	FOLFOX	NR
GPL16686	GSE81653	GSM2165599	FOLFOX	NR
GPL16686	GSE81653	GSM2165600	FOLFOX	NR
GPL16686	GSE81653	GSM2165601	FOLFOX	NR
GPL16686	GSE81653	GSM2165602	FOLFOX	NR
GPL16686	GSE81653	GSM2165603	FOLFOX	NR
GPL16686	GSE81653	GSM2165604	FOLFOX	NR
GPL16686	GSE81653	GSM2165605	FOLFOX	NR
GPL16686	GSE81653	GSM2165606	FOLFOX	NR
GPL16686	GSE81653	GSM2165607	FOLFOX	NR
GPL16686	GSE81653	GSM2165608	FOLFOX	NR
GPL16686	GSE81653	GSM2165609	FOLFOX	NR
GPL16686	GSE81653	GSM2165610	FOLFOX	NR
GPL16686	GSE81653	GSM2165611	FOLFOX	NR
GPL16686	GSE81653	GSM2165612	FOLFOX	NR
GPL16686	GSE81653	GSM2165613	FOLFOX	NR
GPL16686	GSE81653	GSM2165614	FOLFOX	NR
GPL16686	GSE81653	GSM2165615	FOLFOX	NR
GPL16686	GSE81653	GSM2165616	FOLFOX	NR
GPL16686	GSE81653	GSM2165617	FOLFOX	NR
GPL16686	GSE81653	GSM2165618	FOLFOX	NR

plataform	serie.accesion	sample.accession	chemotherapy.type	recurrence.status
GPL16686	GSE81653	GSM2165619	FOLFOX	NR
GPL16686	GSE81653	GSM2165620	FOLFOX	NR
GPL16686	GSE81653	GSM2165621	FOLFOX	NR
GPL16686	GSE81653	GSM2165622	FOLFOX	NR
GPL16686	GSE81653	GSM2165623	FOLFOX	NR
GPL16686	GSE81653	GSM2165624	FOLFOX	NR
GPL16686	GSE81653	GSM2165625	FOLFOX	NR
GPL16686	GSE81653	GSM2165626	FOLFOX	NR
GPL16686	GSE81653	GSM2165627	FOLFOX	NR
GPL16686	GSE81653	GSM2165628	FOLFOX	NR
GPL16686	GSE81653	GSM2165629	FOLFOX	NR
GPL16686	GSE81653	GSM2165630	FOLFOX	NR
GPL16686	GSE81653	GSM2165631	FOLFOX	NR
GPL16686	GSE81653	GSM2165632	FOLFOX	NR
GPL16686	GSE81653	GSM2165633	FOLFOX	NR
GPL570	GSE39582	GSM972039	FOLFOX	R
GPL570	GSE39582	GSM972051	FOLFOX	R
GPL570	GSE39582	GSM972062	FOLFOX	R
GPL570	GSE39582	GSM972066	FOLFOX	R
GPL570	GSE39582	GSM972078	FOLFOX	R
GPL570	GSE39582	GSM972256	FOLFOX	R
GPL570	GSE39582	GSM972291	FOLFOX	R
GPL570	GSE39582	GSM972366	FOLFOX	R
GPL570	GSE39582	GSM972449	FOLFOX	R
GPL570	GSE39582	GSM972474	FOLFOX	R
GPL570	GSE39582	GSM972512	FOLFOX	R
GPL570	GSE72970	GSM1875899	FOLFOX	R
GPL570	GSE72970	GSM1875907	FOLFOX	R
GPL570	GSE72970	GSM1875917	FOLFOX	R
GPL570	GSE72970	GSM1875935	FOLFOX	R
GPL570	GSE72970	GSM1875937	FOLFOX	R
GPL570	GSE72970	GSM1875938	FOLFOX	R
GPL570	GSE72970	GSM1875947	FOLFOX	R
GPL570	GSE72970	GSM1875952	FOLFOX	R
GPL570	GSE72970	GSM1875959	FOLFOX	R
GPL570	GSE72970	GSM1875989	FOLFOX	R
GPL570	GSE72970	GSM1876008	FOLFOX	R
GPL570	GSE72970	GSM1876009	FOLFOX	R
GPL16686	GSE81653	GSM2165468	FOLFOX	R
GPL16686	GSE81653	GSM2165469	FOLFOX	R
GPL16686	GSE81653	GSM2165470	FOLFOX	R
GPL16686	GSE81653	GSM2165471	FOLFOX	R

plataform	serie.accesion	sample.accession	chemotherapy.type	recurrence.status
GPL16686	GSE81653	GSM2165472	FOLFOX	R
GPL16686	GSE81653	GSM2165473	FOLFOX	R
GPL16686	GSE81653	GSM2165474	FOLFOX	R
GPL16686	GSE81653	GSM2165475	FOLFOX	R
GPL16686	GSE81653	GSM2165476	FOLFOX	R
GPL16686	GSE81653	GSM2165477	FOLFOX	R
GPL16686	GSE81653	GSM2165478	FOLFOX	R
GPL16686	GSE81653	GSM2165479	FOLFOX	R
GPL16686	GSE81653	GSM2165480	FOLFOX	R
GPL16686	GSE81653	GSM2165481	FOLFOX	R
GPL16686	GSE81653	GSM2165482	FOLFOX	R
GPL16686	GSE81653	GSM2165483	FOLFOX	R
GPL16686	GSE81653	GSM2165484	FOLFOX	R
GPL16686	GSE81653	GSM2165485	FOLFOX	R
GPL16686	GSE81653	GSM2165486	FOLFOX	R
GPL16686	GSE81653	GSM2165487	FOLFOX	R
GPL16686	GSE81653	GSM2165488	FOLFOX	R
GPL16686	GSE81653	GSM2165489	FOLFOX	R
GPL16686	GSE81653	GSM2165490	FOLFOX	R
GPL16686	GSE81653	GSM2165491	FOLFOX	R
GPL16686	GSE81653	GSM2165492	FOLFOX	R
GPL16686	GSE81653	GSM2165493	FOLFOX	R
GPL16686	GSE81653	GSM2165494	FOLFOX	R
GPL16686	GSE81653	GSM2165495	FOLFOX	R
GPL16686	GSE81653	GSM2165496	FOLFOX	R
GPL16686	GSE81653	GSM2165497	FOLFOX	R
GPL16686	GSE81653	GSM2165498	FOLFOX	R
GPL16686	GSE81653	GSM2165499	FOLFOX	R
GPL16686	GSE81653	GSM2165500	FOLFOX	R
GPL16686	GSE81653	GSM2165501	FOLFOX	R
GPL16686	GSE81653	GSM2165502	FOLFOX	R
GPL16686	GSE81653	GSM2165503	FOLFOX	R
GPL16686	GSE81653	GSM2165504	FOLFOX	R
GPL16686	GSE81653	GSM2165505	FOLFOX	R
GPL16686	GSE81653	GSM2165506	FOLFOX	R
GPL16686	GSE81653	GSM2165507	FOLFOX	R
GPL16686	GSE81653	GSM2165508	FOLFOX	R
GPL16686	GSE81653	GSM2165509	FOLFOX	R
GPL16686	GSE81653	GSM2165510	FOLFOX	R
GPL16686	GSE81653	GSM2165511	FOLFOX	R
GPL16686	GSE81653	GSM2165512	FOLFOX	R
GPL16686	GSE81653	GSM2165513	FOLFOX	R

plataform	serie.accesion	sample.accession	chemotherapy.type	recurrence.status
GPL16686	GSE81653	GSM2165514	FOLFOX	R
GPL16686	GSE81653	GSM2165515	FOLFOX	R
GPL16686	GSE81653	GSM2165516	FOLFOX	R
GPL16686	GSE81653	GSM2165517	FOLFOX	R
GPL16686	GSE81653	GSM2165518	FOLFOX	R
GPL16686	GSE81653	GSM2165519	FOLFOX	R
GPL16686	GSE81653	GSM2165520	FOLFOX	R
GPL16686	GSE81653	GSM2165521	FOLFOX	R
GPL16686	GSE81653	GSM2165522	FOLFOX	R
GPL16686	GSE81653	GSM2165523	FOLFOX	R
GPL16686	GSE81653	GSM2165524	FOLFOX	R
GPL16686	GSE81653	GSM2165525	FOLFOX	R
GPL16686	GSE81653	GSM2165526	FOLFOX	R
GPL16686	GSE81653	GSM2165527	FOLFOX	R
GPL16686	GSE81653	GSM2165528	FOLFOX	R
GPL16686	GSE81653	GSM2165529	FOLFOX	R
GPL16686	GSE81653	GSM2165530	FOLFOX	R
GPL16686	GSE81653	GSM2165531	FOLFOX	R

Table S5. Available clinical data of patients treated with 5-FU monotherapy. Relationship between recurrence condition and available clinicopathological features from patients treated with 5-FU monotherapy selected to perform differential expression analysis.

Variables	Cases (n)	Recurrence		*p* value (fisher test)
		No	Yes	
**GSE39582**				
**Total**	82	53	29	
**Age (years)**				
Mean (SD)	66.02439 (13.84836)	64.75472 (14.19088)	68.34483 (13.12107)	0.803
<60	25	17	8	
>=60	57	36	21	
**TNM stage**				
II	12	12	0	0.006*
III/IV	70	41	29	
T stage				
T2	5	3	2	0.842
T3/T4	77	50	27	
N stage				
Negative	13	12	1	0.026
Positive	69	41	28	
M stage [a]				
Negative	78	51	27	0.881
Positive	3	2	1	
tp53 Mutation [b]				
M	14	11	3	0.890
WT	12	9	3	
kras Mutation [c]				
M	26	16	10	0.418
WT	41	30	11	
braf Mutation [d]				
M	7	5	2	0.940
WT	57	41	16	

a One case wasn't able to characterize
b 85 cases were NA
c 16 cases were NA
d 9 cases were NA
Not available public clinical information for dataset GSE81653 (n=119)

Table S6. GSEA analysis between 5-FU treated recurrent and non-recurrent phenotypes for the GSE39582 dataset. Output table of the GSEA analysis between 5-FU treated recurrent and non-recurrent phenotypes for the GSE39582 dataset. To perform the analysis, the gene sets contained in "*c2.cp.reactome.v2023.1.Hs.symbols.gmt*" and "*c5.go.bp.v2023.1.Hs.symbols.gmt*" databases were used. Only enriched gene sets with an FDR q-val < 0.05 are shown.

GEN SET NAME	ENRICHMENT IN PHENOTYPE	SIZE	ES	NES	NOM p-val	FDR q-val	FWER p-val	RANK AT MAX	LEADING EDGE
GOBP_CELL_CELL_ADHESION_ VIA_PLASMA_MEMBRANE_ ADHESON_MOLECULES	Recurrent	171	0.498	2.31	0.000	0.000	0.000	3545	tags=40%. list=17%. signal=47%
REACTOME_NUCLEAR_EVENTS_ KINASE_AND_TRANSCRIPTION_ FACTOR_ACTIVATION	Recurrent	61	0.52	2.01	0.000	0.027	0.091	2006	tags=26%. list=9%. signal=29%
REACTOME_TGF_BETA_RECEPTO R_ SIGNALING_ACTIVATES_SMADS	Recurrent	46	0.55	2.07	0.000	0.028	0.038	3751	tags=46%. list=18%. signal=55%
REACTOME_PHOSPHORYLATION_ OF_THE_APC_C	Recurrent	20	0.68	2.04	0.000	0.029	0.059	4173	tags=55%. list=19%. signal=68%
REACTOME_NGF_STIMULATED_ TRANSCRIPTION	Recurrent	39	0.57	2.02	0.000	0.029	0.078	2423	tags=36%. list=11%. signal=40%
GOBP_REGULATION_OF_CELLULA R_RESPONSE_TO_TRANSFORMING _GROWTH_FACTOR_BETA_ STIMULUS	Recurrent	142	0.475	2.13	0.000	0.030	0.062	4059	tags=39%. list=19%. signal=47%
GOBP_MICROTUBULE_ANCHORIN G	Recurrent	26	0.684	2.15	0.000	0.032	0.044	3722	tags=54%. list=17%. signal=65%
REACTOME_APC_C_CDC20_MEDIA TED_DEGRADATION_OF_CYCLIN_B	Recurrent	24	0.61	1.92	0.002	0.033	0.242	4173	tags=46%. list=19%. signal=57%
REACTOME_SIGNALING_BY_TGF_ BETA_RECEPTOR_COMPLEX	Recurrent	93	0.47	1.98	0.000	0.034	0.13	4110	tags=39%. list=19%. signal=48%
REACTOME_MITOTIC_G2_G2_M_ PHASES	Recurrent	196	0.42	1.97	0.000	0.034	0.15	5444	tags=41%. list=25%. signal=54%
REACTOME_ELASTIC_FIBRE_ FORMATION	Recurrent	44	0.52	1.91	0.000	0.035	0.273	3632	tags=41%. list=17%. signal=49%

GEN SET NAME	ENRICHMENT IN PHENOTYPE	SIZE	ES	NES	NOM p-val	FDR q-val	FWER p-val	RANK AT MAX	LEADING EDGE
REACTOME_MITOTIC_PROMETAPH ASE	Recurrent	200	0.41	1.95	0.000	0.036	0.184	5444	tags=43%. list=25%. signal=57%
REACTOME_RECRUITMENT_OF_ MITOTIC_CENTROSOME_PROTEINS _ AND_COMPLEXES	Recurrent	81	0.47	1.92	0.000	0.036	0.242	5444	tags=44%. list=25%. signal=59%
REACTOME_RIPK1_MEDIATED_ REGULATED_NECROSIS	Recurrent	29	0.58	1.94	0.000	0.038	0.214	3037	tags=31%. list=14%. signal=36%
REACTOME_SIGNALING_BY_TGFB _ FAMILY_MEMBERS	Recurrent	122	0.44	1.92	0.000	0.038	0.234	4321	tags=39%. list=20%. signal=49%
REACTOME_AURKA_ACTIVATION_ BY_ TPX2	Recurrent	72	0.47	1.9	0.000	0.038	0.317	5444	tags=46%. list=25%. signal=61%
REACTOME_FORMATION_OF_INCIS ION_COMPLEX_IN_GG_NER	Recurrent	43	0.52	1.85	0.000	0.043	0.452	2790	tags=30%. list=13%. signal=35%
REACTOME_RECRUITMENT_OF_NU MA_ TO_MITOTIC_CENTROSOMES	Recurrent	92	0.44	1.84	0.000	0.045	0.486	5444	tags=43%. list=25%. signal=58%
REACTOME_APC_CDC20_MEDIATE D_ DEGRADATION_OF_NEK2A	Recurrent	26	0.57	1.84	0.000	0.045	0.496	6328	tags=58%. list=30%. signal=82%
REACTOME_RHOBTB_GTPASE_CY CLE	Recurrent	35	0.54	1.86	0.000	0.046	0.439	6983	tags=57%. list=33%. signal=85%
REACTOME_REGULATION_OF_PLK 1_ ACTIVITY_AT_G2_M_TRANSITION	Recurrent	87	0.51	2.1	0.000	0.047	0.032	5444	tags=48%. list=25%. signal=64%
REACTOME_M_PHASE	Recurrent	407	0.37	1.86	0.000	0.047	0.409	5105	tags=36%. list=24%. signal=47%
REACTOME_MOLECULES_ASSOCI ATED_WITH_ELASTIC_FIBRES	Recurrent	37	0.53	1.86	0.002	0.047	0.424	3632	tags=46%. list=17%. signal=55%
REACTOME_RHOU_GTPASE_CYCL E	Recurrent	40	0.52	1.87	0.002	0.048	0.394	5805	tags=52%. list=27%. signal=72%

GEN SET NAME	ENRICHMENT IN PHENOTYPE	SIZE	ES	NES	NOM p-val	FDR q-val	FWER p-val	RANK AT MAX	LEADING EDGE
REACTOME_OLFACTORY_SIGNALING_ PATHWAY	Non recurrent	105	-0.54	-2.24	0.000	0.000	0.000	5195	tags=57%. list=24%. signal=75%
REACTOME_ASPIRIN_ADME	Non recurrent	38	-0.66	-2.23	0.000	0.000	0.000	1637	tags=50%. list=8%. signal=54%
REACTOME_DIGESTION_AND_ ABSORPTION	Non recurrent	27	-0.69	-2.18	0.000	0.001	0.003	1660	tags=52%. list=8%. signal=56%
GOBP_SENSORY_PERCEPTION_OF_ _ SMELL	Non recurrent	122	-0.52	-2.23	0.000	0.002	0.003	5195	tags=52%. list=24%. signal=68%
GOBP_CELLULAR_GLUCURONIDATION	Non recurrent	18	-0.76	-2.18	0.000	0.003	0.008	786	tags=56%. list=4%. signal=58%
REACTOME_CHOLESTEROL_ BIOSYNTHESIS	Non recurrent	27	-0.65	-2.06	0.000	0.004	0.027	3480	tags=52%. list=16%. signal=62%
REACTOME_GLUCURONIDATION	Non recurrent	21	-0.69	-2.05	0.000	0.004	0.036	1637	tags=52%. list=8%. signal=57%
REACTOME_DRUG_ADME	Non recurrent	92	-0.5	-2.03	0.000	0.004	0.041	2609	tags=37%. list=12%. signal=42%
REACTOME_PEPTIDE_LIGAND_BINDING_RECEPTORS	Non recurrent	193	-0.44	-2.01	0.000	0.005	0.057	4835	tags=40%. list=23%. signal=51%
REACTOME_DIGESTION	Non recurrent	23	-0.65	-2	0.000	0.005	0.07	1660	tags=48%. list=8%. signal=52%
REACTOME_CHEMOKINE_RECEPTORS_BIND_CHEMOKINES	Non recurrent	58	-0.53	-1.99	0.000	0.005	0.075	3291	tags=40%. list=15%. signal=47%
REACTOME_CLASS_A_1_RHODOPSIN_ LIKE_RECEPTORS	Non recurrent	324	-0.42	-1.98	0.00	0.005	0.082	4929	tags=40%. list=23%. signal=51%
GOBP_CELLULAR_AMINO_ACID_ CATABOLIC_PROCESS	Non recurrent	113	-0.51	-2.12	0.000	0.006	0.035	5350	tags=50%. list=25%. signal=66%

GEN SET NAME	ENRICHMENT IN PHENOTYPE	SIZE	ES	NES	NOM p-val	FDR q-val	FWER p-val	RANK AT MAX	LEADING EDGE
GOBP_3_UTR_MEDIATED_MRNA_ STABILIZATION	Non recurrent	24	-0.69	-2.13	0.000	0.007	0.032	1361	tags=25%. list=6%. signal=27%
GOBP_ASPARTATE_FAMILY_AMINO_ ACID_CATABOLIC_PROCESS	Non recurrent	18	-0.73	-2.09	0.000	0.007	0.052	3271	tags=61%. list=15%. signal=72%
REACTOME_COMPLEMENT_CASCADE	Non recurrent	91	-0.47	-1.91	0.000	0.011	0.204	3451	tags=36%. list=16%. signal=43%
REACTOME_REGULATION_OF_GENE_ EXPRESSION_IN_BETA_CELLS	Non recurrent	20	-0.64	-1.92	0.000	0.012	0.19	2286	tags=40%. list=11%. signal=45%
REACTOME_SYNTHESIS_OF_BILE_ ACIDS_AND_BILE_SALTS_VIA_7ALPHA_HYDROXYCHOLESTEROL	Non recurrent	24	-0.62	-1.89	0.000	0.012	0.244	2158	tags=46%. list=10%. signal=51%
REACTOME_GPCR_LIGAND_BINDING	Non recurrent	448	-0.38	-1.88	0.000	0.013	0.27	6482	tags=46%. list=30%. signal=65%
GOBP_URONIC_ACID_METABOLIC_ PROCESS	Non recurrent	23	-0.66	-2.03	0.000	0.013	0.126	2956	tags=57%. list=14%. signal=66%
GOBP_ORGANIC_ACID_CATABOLIC_ PROCESS	Non recurrent	246	-0.44	-2.02	0.000	0.013	0.149	5615	tags=46%. list=26%. signal=62%
GOBP_ALPHA_AMINO_ACID_ CATABOLIC_PROCESS	Non recurrent	95	-0.50	-2.04	0.000	0.014	0.121	5350	tags=53%. list=25%. signal=70%
REACTOME_REGULATION_OF_BETA_ CELL_DEVELOPMENT	Non recurrent	40	-0.54	-1.87	0.000	0.015	0.312	4036	tags=43%. list=19%. signal=52%
REACTOME_BIOLOGICAL_OXIDATIONS	Non recurrent	211	-0.4	-1.84	0.000	0.02	0.422	4496	tags=38%. list=21%. signal=48%
REACTOME_PHASE_4_RESTING_ MEMBRANE_POTENTIAL	Non recurrent	18	-0.64	-1.83	0.004	0.02	0.45	3429	tags=50%. list=16%. signal=59%
REACTOME_SENSORY_PERCEPTION	Non recurrent	313	-0.38	-1.82	0.000	0.022	0.511	5255	tags=39%. list=25%. signal=50%

GEN SET NAME	ENRICHMENT IN PHENOTYPE	SIZE	ES	NES	NOM p-val	FDR q-val	FWER p-val	RANK AT MAX	LEADING EDGE
REACTOME_PROSTACYCLIN_ SIGNALLING_THROUGH_ PROSTACYCLIN_RECEPTOR	Non recurrent	19	-0.63	-1.82	0.004	0.022	0.525	2735	tags=42%. list=13%. signal=48%
GOBP_RESPONSE_TO_CHEMOKIN E	Non recurrent	97	-0.48	-1.98	0.000	0.023	0.272	2459	tags=32%. list=11%. signal=36%
GOBP_POSITIVE_REGULATION_OF _ TRANSLATIONAL_INITIATION	Non recurrent	31	-0.61	-1.97	0.000	0.025	0.315	307	tags=16%. list=1%. signal=16%
REACTOME_G_ALPHA_I_SIGNALLI NG_ EVENTS	Non recurrent	302	-0.37	-1.8	0.000	0.027	0.612	3835	tags=29%. list=18%. signal=35%
GOBP_ACUTE_PHASE_RESPONSE	Non recurrent	48	-0.53	-1.93	0.000	0.028	0.525	1758	tags=31%. list=8%. signal=34%
GOBP_ALDITOL_METABOLIC_PRO CESS	Non recurrent	22	-0.64	-1.94	0.000	0.029	0.459	3408	tags=41%. list=16%. signal=49%
GOBP_CELLULAR_RESPONSE_TO_ XENOBIOTIC_STIMULUS	Non recurrent	180	-0.43	-1.93	0.000	0.029	0.521	4083	tags=38%. list=19%. signal=47%
GOBP_DETECTION_OF_STIMULUS_ INVOLVED_IN_SENSORY_PERCEPT ION	Non recurrent	206	-0.42	-1.94	0.000	0.03	0.455	4847	tags=43%. list=23%. signal=55%
GOBP_HEPATICOBILIARY_SYSTEM _ DEVELOPMENT	Non recurrent	143	-0.45	-1.94	0.000	0.031	0.432	4402	tags=36%. list=21%. signal=45%
GOBP_ALPHA_AMINO_ACID_ METABOLIC_PROCESS	Non recurrent	204	-0.42	-1.93	0.000	0.031	0.518	3398	tags=31%. list=16%. signal=37%
REACTOME_BILE_ACID_AND_BILE _ SALT_METABOLISM	Non recurrent	45	-0.5	-1.78	0.000	0.032	0.685	2609	tags=40%. list=12%. signal=45%
REACTOME_REGULATION_OF_IFN A_IFNB_SIGNALING	Non recurrent	26	-0.57	-1.77	0.005	0.032	0.709	4984	tags=50%. list=23%. signal=65%
GOBP_CELLULAR_AMINO_ACID_ METABOLIC_PROCESS	Non recurrent	287	-0.40	-1.91	0.000	0.032	0.607	3427	tags=29%. list=16%. signal=34%

GEN SET NAME	ENRICHMENT IN PHENOTYPE	SIZE	ES	NES	NOM p-val	FDR q-val	FWER p-val	RANK AT MAX	LEADING EDGE
GOBP_SENSORY_PERCEPTION_OF_ _ CHEMICAL_STIMULUS	Non recurrent	193	-0.42	-1.89	0.000	0.032	0.675	5729	tags=49%. list=27%. signal=66%
GOBP_HUMORAL_IMMUNE_RESPO NSE	Non recurrent	284	-0.41	-1.95	0.000	0.033	0.424	3129	tags=31%. list=15%. signal=35%
GOBP_ASPARTATE_FAMILY_AMIN O_ ACID_METABOLIC_PROCESS	Non recurrent	48	-0.53	-1.91	0.000	0.033	0.633	3299	tags=33%. list=15%. signal=39%
GOBP_NEUTROPHIL_CHEMOTAXIS	Non recurrent	103	-0.46	-1.9	0.000	0.033	0.648	3291	tags=33%. list=15%. signal=39%
GOBP_XENOBIOTIC_METABOLIC_ PROCESS	Non recurrent	115	-0.45	-1.9	0.000	0.034	0.671	5775	tags=55%. list=27%. signal=75%
GOBP_COMPLEMENT_ACTIVATION	Non recurrent	98	-0.46	-1.88	0.000	0.037	0.744	4039	tags=38%. list=19%. signal=46%
GOBP_ANTIMICROBIAL_HUMORAL _ IMMUNE_RESPONSE_MEDIATED_B Y_ ANTIMICROBIAL_PEPTIDE	Non recurrent	81	-0.48	-1.87	0.000	0.04	0.78	2046	tags=32%. list=10%. signal=35%
GOBP_KILLING_BY_HOST_OF_ SYMBIONT_CELLS	Non recurrent	29	-0.57	-1.85	0.002	0.042	0.845	1593	tags=34%. list=7%. signal=37%
GOBP_XENOBIOTIC_CATABOLIC_ PROCESS	Non recurrent	26	-0.59	-1.86	0.002	0.043	0.811	4913	tags=65%. list=23%. signal=85%
GOBP_LIVER_REGENERATION	Non recurrent	29	-0.59	-1.86	0.000	0.043	0.829	4258	tags=34%. list=20%. signal=43%
GOBP_GRANULOCYTE_CHEMOTAX IS	Non recurrent	127	-0.43	-1.85	0.000	0.044	0.845	3291	tags=31%. list=15%. signal=37%
GOBP_HUMORAL_IMMUNE_RESPO NSE_MEDIATED_BY_CIRCULATING _IMMUNOGLOBULIN	Non recurrent	86	-0.45	-1.85	0.002	0.044	0.861	3937	tags=36%. list=18%. signal=44%

GEN SET NAME	ENRICHMENT IN PHENOTYPE	SIZE	ES	NES	NOM p-val	FDR q-val	FWER p-val	RANK AT MAX	LEADING EDGE
GOBP_SECONDARY_ALCOHOL_ METABOLIC_PROCESS	Non recurrent	145	-0.42	-1.84	0.000	0.045	0.892	6468	tags=49%. list=30%. signal=70%
GOBP_DETECTION_OF_CHEMICAL _ STIMULUS	Non recurrent	175	-0.41	-1.84	0.000	0.046	0.892	5195	tags=46%. list=24%. signal=60%
REACTOME_G_BETA_GAMMA_ SIGNALLING_THROUGH_PI3KGAM MA	Non recurrent	25	-0.55	-1.73	0.010	0.047	0.856	2735	tags=32%. list=13%. signal=37%
GOBP_ANTIMICROBIAL_HUMORAL _ RESPONSE	Non recurrent	123	-0.43	-1.83	0.000	0.047	0.905	3551	tags=34%. list=17%. signal=41%
GOBP_RESPONSE_TO_INTERLEUK IN_6	Non recurrent	36	-0.54	-1.83	0.000	0.048	0.921	2869	tags=39%. list=13%. signal=45%
REACTOME_HEME_DEGRADATION	Non recurrent	15	-0.63	-1.72	0.011	0.048	0.872	2609	tags=60%. list=12%. signal=68%

Table S7. GSEA analysis between 5-FU treated recurrent and non-recurrent phenotypes for the GSE81653 dataset. Output table of the GSEA analysis between 5-FU treated recurrent and non-recurrent phenotypes for the GSE81653 dataset. To perform the analysis, the gene sets contained in "*c2.cp.reactome.v2023.1.Hs.symbols.gmt*" and "*c5.go.bp.v2023.1.Hs.symbols.gmt*" databases were used. Only enriched gene sets with an FDR q-val < 0.05 are shown.

GEN SET NAME	ENRICHMENT IN PHENOTYPE	SIZE	ES	NES	NOM p-val	FDR q-val	FWER p-val	RANK AT MAX	LEADING EDGE
REACTOME_SMOOTH_MUSCLE_CO NTRACTION	Recurrent	43	0.590	2.210	0.000	0.006	0.006	5467	tags=47% list=21% signal=59%
REACTOME_LAMININ_ INTERACTIONS	Recurrent	30	0.610	2.070	0.000	0.013	0.036	6621	tags=50%. list=26%. signal=67%
REACTOME_CGMP_EFFECTS	Recurrent	16	0.730	2.080	0.000	0.018	0.033	3993	tags=63%. list=15%. signal=74%

GEN SET NAME	ENRICHMENT IN PHENOTYPE	SIZE	ES	NES	NOM p-val	FDR q-val	FWER p-val	RANK AT MAX	LEADING EDGE
REACTOME_EPHA_MEDIATED_GROWTH_CONE_COLLAPSE	Recurrent	29	0.590	2.020	0.000	0.023	0.084	4163	tags=45%. list=16%. signal=53%
GOBP_CELLULAR_RESPONSE_TO_ACIDIC_PH	Recurrent	17	0.750	2.190	0.000	0.026	0.020	4239	tags=65%. list=16%. signal=77%
REACTOME_NON_INTEGRIN_MEMBRANE_ECM_INTERACTIONS	Recurrent	59	0.490	1.970	0.000	0.032	0.144	2607	tags=27%. list=10%. signal=30%
REACTOME_NITRIC_OXIDE_STIMULATES_GUANYLATE_CYCLASE	Recurrent	22	0.620	1.940	0.000	0.036	0.221	5467	tags=55%. list=21%. signal=69%
REACTOME_MUSCLE_CONTRACTION	Recurrent	203	0.400	1.940	0.000	0.037	0.199	5559	tags=33%. list=21%. signal=42%
REACTOME_SCAVENGING_OF_HEME_FROM_PLASMA	Non recurrent	23	-0.700	-2.200	0.000	0.001	0.003	975	tags=48%. list=4%. signal=50%
REACTOME_ANTIGEN_ACTIVATES_B_CELL_RECEPTOR_BCR_LEADING_TO_GENERATION_OF_SECOND_MESSENGERS	Non recurrent	40	-0.630	-2.200	0.000	0.001	0.003	4731	tags=45%. list=18%. signal=55%
REACTOME_CHEMOKINE_RECEPTORS_BIND_CHEMOKINES	Non recurrent	58	-0.580	-2.240	0.000	0.002	0.002	4094	tags=45%. list=16%. signal=53%
REACTOME_FCGR_ACTIVATION	Non recurrent	23	-0.700	-2.140	0.000	0.002	0.008	3454	tags=57%. list=13%. signal=65%
REACTOME_CD22_MEDIATED_BCR_REGULATION	Non recurrent	15	-0.770	-2.120	0.000	0.002	0.009	4731	tags=80%. list=18%. signal=98%
REACTOME_HOMOLOGOUS_DNA_PAIRING_AND_STRAND_EXCHANGE	Non recurrent	43	-0.580	-2.100	0.000	0.002	0.011	3264	tags=40%. list=13%. signal=45%
GOBP_B_CELL_RECEPTOR_SIGNALING_PATHWAY	Non recurrent	74	-0.570	-2.280	0.000	0.002	0.002	4731	tags=45%. list=18%. signal=54%
REACTOME_TELOMERE_C_STRAND_LAGGING_STRAND_SYNTHESIS	Non recurrent	34	-0.590	-2.000	0.000	0.007	0.066	5399	tags=44%. list=21%. signal=56%

GEN SET NAME	ENRICHMENT IN PHENOTYPE	SIZE	ES	NES	NOM p-val	FDR q-val	FWER p-val	RANK AT MAX	LEADING EDGE
GOBP_PHAGOCYTOSIS_ RECOGNITION	Non recurrent	47	-0.600	-2.170	0.000	0.007	0.018	4628	tags=49%. list=18%. signal=59%
REACTOME_CREATION_OF_C4_ AND_C2_ACTIVATORS	Non recurrent	25	-0.620	-2.010	0.000	0.008	0.063	4296	tags=56%. list=17%. signal=67%
REACTOME_DNA_STRAND_ ELONGATION	Non recurrent	32	-0.590	-1.980	0.000	0.009	0.087	6319	tags=53%. list=24%. signal=70%
REACTOME_ROLE_OF_ PHOSPHOLIPIDS_IN_ PHAGOCYTOSIS	Non recurrent	36	-0.570	-1.980	0.000	0.009	0.089	4296	tags=39%. list=17%. signal=47%
REACTOME_ACTIVATION_OF_ATR_ IN_RESPONSE_TO_REPLICATION_ STRESS	Non recurrent	37	-0.560	-1.960	0.000	0.010	0.120	1919	tags=30%. list=7%. signal=32%
REACTOME_IMMUNOREGULATORY _INTERACTIONS_BETWEEN_A_ LYMPHOID_AND_A_NON_ LYMPHOID_CELL	Non recurrent	140	-0.440	-1.940	0.000	0.012	0.153	5170	tags=39%. list=20%. signal=49%
REACTOME_INTERLEUKIN_10_ SIGNALING	Non recurrent	46	-0.520	-1.920	0.000	0.015	0.217	4199	tags=39%. list=16%. signal=47%
REACTOME_POLYMERASE_ SWITCHING_ON_THE_C_STRAND_ OF_THE_TELOMERE	Non recurrent	26	-0.600	-1.920	0.000	0.016	0.208	7575	tags=54%. list=29%. signal=76%
REACTOME_HDR_THROUGH_ SINGLE_STRAND_ANNEALING_SSA	Non recurrent	37	-0.550	-1.900	0.000	0.018	0.255	5399	tags=49%. list=21%. signal=61%
REACTOME_ACTIVATION_OF_THE_ PRE_REPLICATIVE_COMPLEX	Non recurrent	33	-0.540	-1.840	0.000	0.028	0.456	5080	tags=42%. list=20%. signal=53%
REACTOME_SIGNALING_BY_THE_ B_CELL_RECEPTOR_BCR	Non recurrent	120	-0.420	-1.840	0.002	0.028	0.468	5658	tags=35%. list=22%. signal=45%
REACTOME_RESOLUTION_OF_D_ LOOP_STRUCTURES	Non recurrent	35	-0.540	-1.850	0.002	0.029	0.437	3264	tags=37%. list=13%. signal=42%
REACTOME_FCGR3A_MEDIATED_ IL10_SYNTHESIS	Non recurrent	49	-0.500	-1.840	0.000	0.029	0.456	4296	tags=37%. list=17%. signal=44%

GEN SET NAME	ENRICHMENT IN PHENOTYPE	SIZE	ES	NES	NOM p-val	FDR q-val	FWER p-val	RANK AT MAX	LEADING EDGE
**REACTOME_NUCLEAR_ENVELOPE_BREAKDOWN**	Non recurrent	53	-0.490	-1.840	0.000	0.029	0.503	7982	tags=55%. list=31%. signal=79%
**GOBP_DNA_REPLICATION_INITIATION**	Non recurrent	38	-0.590	-2.040	0.000	0.044	0.158	5169	tags=50%. list=20%. signal=62%
**GOBP_SODIUM_INDEPENDENT_ORGANIC_ANION_TRANSPORT**	Non recurrent	15	-0.700	-1.990	0.002	0.047	0.314	3584	tags=60%. list=14%. signal=70%
**REACTOME_LAGGING_STRAND_SYNTHESIS**	Non recurrent	20	-0.600	-1.790	0.007	0.048	0.696	6319	tags=50%. list=24%. signal=66%

Table S8. DEGs identified in patients with tumor recurrence after 5-FU therapy. Common DEGs identified in patient with tumor recurrence after 5-FU therapy in both GSE39582 and GSE81653 datasets independently by two or more methods (|log_2FC|>1 and an FDR < 0.01).

ENTEREZ_ID	GENE_NAME	GENE_SYMBOL	STATE
23157	septin 6	SEP6	upregulated
989	septin 7	SEP7	upregulated
25890	ABI family member 3 binding protein	ABI3BP	upregulated
3983	actin binding LIM protein 1	ABLIM1	upregulated
59272	angiotensin I converting enzyme 2	ACE2	upregulated
72	actin, gamma 2, smooth muscle, enteric	ACTG2	upregulated
8038	ADAM metallopeptidase domain 12	ADAM12	upregulated
27299	ADAM like decysin 1	ADAMDEC1	upregulated
9510	ADAM metallopeptidase with thrombospondin type 1 motif 1	ADAMTS1	upregulated
100507098	ADAMTS9 antisense RNA 2	ADAMTS9-AS2	upregulated
84873	adhesion G protein-coupled receptor G7	ADGRG7	upregulated
125	alcohol dehydrogenase 1B (class I), beta polypeptide	ADH1B	upregulated
126	alcohol dehydrogenase 1C (class I), gamma polypeptide	ADH1C	upregulated
84830	androgen dependent TFPI regulating protein	ADTRP	upregulated
119016	ArfGAP with GTPase domain, ankyrin repeat and PH domain 4	AGAP4	upregulated
155465	anterior gradient 3, protein disulphide isomerase family member	AGR3	upregulated

ENTEREZ_ID	GENE_NAME	GENE_SYMBOL	STATE
130872	AHA1, activator of heat shock 90kDa protein ATPase homolog 2 (yeast)	AHSA2	upregulated
11214	A-kinase anchoring protein 13	AKAP13	upregulated
57016	aldo-keto reductase family 1 member B10	AKR1B10	upregulated
55608	ankyrin repeat domain 10	ANKRD10	upregulated
23253	ankyrin repeat domain 12	ANKRD12	upregulated
284232	ankyrin repeat domain 20 family member A9, pseudogene	ANKRD20A9P	upregulated
23243	ankyrin repeat domain 28	ANKRD28	upregulated
57730	ankyrin repeat domain 36B	ANKRD36B	upregulated
645784	ankyrin repeat domain 36B pseudogene 2	ANKRD36BP2	upregulated
301	annexin A1	ANXA1	upregulated
339	apolipoprotein B mRNA editing enzyme catalytic subunit 1	APOBEC1	upregulated
394	Rho GTPase activating protein 5	ARHGAP5	upregulated
54829	asporin	ASPN	upregulated
467	activating transcription factor 3	ATF3	upregulated
493	ATPase plasma membrane Ca2+ transporting 4	ATP2B4	upregulated
8992	ATPase H+ transporting V0 subunit e1	ATP6V0E1	upregulated
546	ATRX, chromatin remodeler	ATRX	upregulated
25805	BMP and activin membrane bound inhibitor	BAMBI	upregulated
9031	bromodomain adjacent to zinc finger domain 1B	BAZ1B	upregulated
8537	breast carcinoma amplified sequence 1	BCAS1	upregulated
440603	BCL2 like 15	BCL2L15	upregulated
8553	basic helix-loop-helix family member e40	BHLHE40	upregulated
55589	BMP2 inducible kinase	BMP2K	upregulated
642826	BMS1, ribosome biogenesis factor pseudogene 6	BMS1P6	upregulated
259282	biorientation of chromosomes in cell division 1 like 1	BOD1L1	upregulated
6046	bromodomain containing 2	BRD2	upregulated
55727	BTB domain containing 7	BTBD7	upregulated
79908	butyrophilin like 8	BTNL8	upregulated
387695	chromosome 10 open reading frame 99	C10orf99	upregulated
84419	chromosome 15 open reading frame 48	C15orf48	upregulated
79002	chromosome 19 open reading frame 43	C19orf43	upregulated
718	complement C3	C3	upregulated
720	complement C4A (Rodgers blood group)	C4A	upregulated
721	complement C4B (Chido blood group)	C4B	upregulated
100293534	complement component 4B (Chido blood group), copy 2	C4B_2	upregulated
730	complement C7	C7	upregulated
56892	chromosome 8 open reading frame 4	C8orf4	upregulated
100127983	chromosome 8 open reading frame 88	C8orf88	upregulated

ENTEREZ_ID	GENE_NAME	GENE_SYMBOL	STATE
760	carbonic anhydrase 2	CA2	upregulated
762	carbonic anhydrase 4	CA4	upregulated
800	caldesmon 1	CALD1	upregulated
55450	calcium/calmodulin dependent protein kinase II inhibitor 1	CAMK2N1	upregulated
728264	cardiac mesoderm enhancer-associated non-coding RNA	CARMN	upregulated
113201	cancer susceptibility candidate 4	CASC4	upregulated
837	caspase 4	CASP4	upregulated
84869	carbonyl reductase 4	CBR4	upregulated
55749	cell division cycle and apoptosis regulator 1	CCAR1	upregulated
57639	coiled-coil domain containing 146	CCDC146	upregulated
152137	coiled-coil domain containing 50	CCDC50	upregulated
6356	C-C motif chemokine ligand 11	CCL11	upregulated
6347	C-C motif chemokine ligand 2	CCL2	upregulated
56477	C-C motif chemokine ligand 28	CCL28	upregulated
57018	cyclin L1	CCNL1	upregulated
57126	CD177 molecule	CD177	upregulated
960	CD44 molecule (Indian blood group)	CD44	upregulated
961	CD47 molecule	CD47	upregulated
1604	CD55 molecule (Cromer blood group)	CD55	upregulated
965	CD58 molecule	CD58	upregulated
966	CD59 molecule	CD59	upregulated
401577	CD99 molecule pseudogene 1	CD99P1	upregulated
8476	CDC42 binding protein kinase alpha	CDC42BPA	upregulated
1087	carcinoembryonic antigen related cell adhesion molecule 7	CEACAM7	upregulated
9731	centrosomal protein 104	CEP104	upregulated
90799	centrosomal protein 95	CEP95	upregulated
3075	complement factor H	CFH	upregulated
1073	cofilin 2	CFL2	upregulated
1106	chromodomain helicase DNA binding protein 2	CHD2	upregulated
63928	calcineurin like EF-hand protein 2	CHP2	upregulated
91851	chordin like 1	CHRDL1	upregulated
1179	chloride channel accessory 1	CLCA1	upregulated
22802	chloride channel accessory 4	CLCA4	upregulated
9073	claudin 8	CLDN8	upregulated
9685	clathrin interactor 1	CLINT1	upregulated
57396	CDC like kinase 4	CLK4	upregulated
1264	calponin 1	CNN1	upregulated
10330	canopy FGF signaling regulator 2	CNPY2	upregulated
1270	ciliary neurotrophic factor	CNTF	upregulated

ENTEREZ_ID	GENE_NAME	GENE_SYMBOL	STATE
7373	collagen type XIV alpha 1 chain	COL14A1	upregulated
1346	cytochrome c oxidase subunit 7A1	COX7A1	upregulated
83716	cysteine rich secretory protein LCCL domain containing 2	CRISPLD2	upregulated
1415	crystallin beta B2	CRYBB2	upregulated
79848	centrosome and spindle pole associated protein 1	CSPP1	upregulated
1499	catenin beta 1	CTNNB1	upregulated
1519	cathepsin O	CTSO	upregulated
6387	C-X-C motif chemokine ligand 12	CXCL12	upregulated
9547	C-X-C motif chemokine ligand 14	CXCL14	upregulated
4283	C-X-C motif chemokine ligand 9	CXCL9	upregulated
100290481	immunoglobulin lambda light chain-like	CYAT1	upregulated
54205	cytochrome c, somatic	CYCS	upregulated
1562	cytochrome P450 family 2 subfamily C member 18	CYP2C18	upregulated
1577	cytochrome P450 family 3 subfamily A member 5	CYP3A5	upregulated
23002	dishevelled associated activator of morphogenesis 1	DAAM1	upregulated
54876	DDB1 and CUL4 associated factor 16	DCAF16	upregulated
1634	decorin	DCN	upregulated
10521	DEAD-box helicase 17	DDX17	upregulated
57062	DEAD-box helicase 24	DDX24	upregulated
1656	DEAD-box helicase 6	DDX6	upregulated
1671	defensin alpha 6	DEFA6	upregulated
163486	DENN domain containing 1B	DENND1B	upregulated
1674	desmin	DES	upregulated
1803	dipeptidyl peptidase 4	DPP4	upregulated
1830	desmoglein 3	DSG3	upregulated
667	dystonin	DST	upregulated
1843	dual specificity phosphatase 1	DUSP1	upregulated
503639	double homeobox A pseudogene 10	DUXAP10	upregulated
100506710	endogenous Bornavirus-like nucleoprotein 3, pseudogene	EBLN3P	upregulated
10085	EGF like repeats and discoidin domains 3	EDIL3	upregulated
1917	eukaryotic translation elongation factor 1 alpha 2	EEF1A2	upregulated
2202	EGF containing fibulin like extracellular matrix protein 1	EFEMP1	upregulated
1948	ephrin B2	EFNB2	upregulated
1956	epidermal growth factor receptor	EGFR	upregulated
1958	early growth response 1	EGR1	upregulated
1975	eukaryotic translation initiation factor 4B	EIF4B	upregulated
2012	epithelial membrane protein 1	EMP1	upregulated
5168	ectonucleotide pyrophosphatase/phosphodiesterase 2	ENPP2	upregulated
2045	EPH receptor A7	EPHA7	upregulated

ENTEREZ_ID	GENE_NAME	GENE_SYMBOL	STATE
54206	ERBB receptor feedback inhibitor 1	ERRFI1	upregulated
2114	ETS proto-oncogene 2, transcription factor	ETS2	upregulated
54536	exocyst complex component 6	EXOC6	upregulated
2168	fatty acid binding protein 1	FABP1	upregulated
2170	fatty acid binding protein 3	FABP3	upregulated
92689	family with sequence similarity 114 member A1	FAM114A1	upregulated
116496	family with sequence similarity 129 member A	FAM129A	upregulated
10144	family with sequence similarity 13 member A	FAM13A	upregulated
222234	family with sequence similarity 185 member A	FAM185A	upregulated
54906	family with sequence similarity 208 member B	FAM208B	upregulated
54097	family with sequence similarity 3 member B	FAM3B	upregulated
10447	family with sequence similarity 3 member C	FAM3C	upregulated
131177	family with sequence similarity 3 member D	FAM3D	upregulated
100216479	fatty acyl-CoA reductase 2 pseudogene 2	FAR2P2	upregulated
260436	follicular dendritic cell secreted protein	FDCSP	upregulated
10979	fermitin family member 2	FERMT2	upregulated
2252	fibroblast growth factor 7	FGF7	upregulated
23048	formin binding protein 1	FNBP1	upregulated
2353	Fos proto-oncogene, AP-1 transcription factor subunit	FOS	upregulated
2354	FosB proto-oncogene, AP-1 transcription factor subunit	FOSB	upregulated
93986	forkhead box P2	FOXP2	upregulated
8087	FMR1 autosomal homolog 1	FXR1	upregulated
2564	gamma-aminobutyric acid type A receptor epsilon subunit	GABRE	upregulated
2589	polypeptide N-acetylgalactosaminyltransferase 1	GALNT1	upregulated
57733	glucosylceramidase beta 3 (gene/pseudogene)	GBA3	upregulated
54834	ganglioside induced differentiation associated protein 2	GDAP2	upregulated
2669	GTP binding protein overexpressed in skeletal muscle	GEM	upregulated
2734	golgi glycoprotein 1	GLG1	upregulated
11010	GLI pathogenesis related 1	GLIPR1	upregulated
169792	GLIS family zinc finger 3	GLIS3	upregulated
2803	golgin A4	GOLGA4	upregulated
157	G protein-coupled receptor kinase 3	GRK3	upregulated
3039	hemoglobin subunit alpha 1	HBA1	upregulated
25831	HECT domain E3 ubiquitin protein ligase 1	HECTD1	upregulated
8352	histone cluster 1 H3 family member c	HIST1H3C	upregulated
8968	histone cluster 1 H3 family member f	HIST1H3F	upregulated
3097	human immunodeficiency virus type I enhancer binding protein 2	HIVEP2	upregulated

ENTEREZ_ID	GENE_NAME	GENE_SYMBOL	STATE
3117	major histocompatibility complex, class II, DQ alpha 1	HLA-DQA1	upregulated
3122	major histocompatibility complex, class II, DR alpha	HLA-DRA	upregulated
3123	major histocompatibility complex, class II, DR beta 1	HLA-DRB1	upregulated
84376	hook microtubule tethering protein 3	HOOK3	upregulated
3215	homeobox B5	HOXB5	upregulated
3216	homeobox B6	HOXB6	upregulated
3219	homeobox B9	HOXB9	upregulated
3248	hydroxyprostaglandin dehydrogenase 15-(NAD)	HPGD	upregulated
3294	hydroxysteroid 17-beta dehydrogenase 2	HSD17B2	upregulated
126917	intermediate filament family orphan 2	IFFO2	upregulated
3479	insulin like growth factor 1	IGF1	upregulated
3492	immunoglobulin heavy locus	IGH	upregulated
3500	immunoglobulin heavy constant gamma 1 (G1m marker)	IGHG1	upregulated
3537	immunoglobulin lambda constant 1	IGLC1	upregulated
28831	immunoglobulin lambda joining 3	IGLJ3	upregulated
100505573	InaF motif containing 2	INAFM2	upregulated
27130	inversin	INVS	upregulated
3672	integrin subunit alpha 1	ITGA1	upregulated
3707	inositol-trisphosphate 3-kinase B	ITPKB	upregulated
221037	jumonji domain containing 1C	JMJD1C	upregulated
3728	junction plakoglobin	JUP	upregulated
25959	KN motif and ankyrin repeat domains 2	KANK2	upregulated
23325	KIAA1033	KIAA1033	upregulated
4297	lysine methyltransferase 2A	KMT2A	upregulated
3845	KRAS proto-oncogene, GTPase	KRAS	upregulated
54474	keratin 20	KRT20	upregulated
25984	keratin 23	KRT23	upregulated
3977	leukemia inhibitory factor receptor alpha	LIFR	upregulated
51474	LIM domain and actin binding 1	LIMA1	upregulated
200879	lipase H	LIPH	upregulated
25802	leiomodin 1	LMOD1	upregulated
100288570	glycosylphosphatidylinositol anchor attachment protein 1 homolog (yeast) pseudogene	LOC100288570	upregulated
100507334	two pore channel 3 pseudogene	LOC100507334	upregulated
100507412	uncharacterized LOC100507412	LOC100507412	upregulated
440895	two pore channel 3 pseudogene	LOC440895	upregulated
645513	septin 7 pseudogene	LOC645513	upregulated
4026	LIM domain containing preferred translocation partner in lipoma	LPP	upregulated

ENTEREZ_ID	GENE_NAME	GENE_SYMBOL	STATE
84859	leucine rich repeats and calponin homology domain containing 3	LRCH3	upregulated
4052	latent transforming growth factor beta binding protein 1	LTBP1	upregulated
646627	LY6/PLAUR domain containing 8	LYPD8	upregulated
10586	mab-21 like 2	MAB21L2	upregulated
23499	microtubule-actin crosslinking factor 1	MACF1	upregulated
4094	MAF bZIP transcription factor	MAF	upregulated
84441	mastermind like transcriptional coactivator 2	MAML2	upregulated
1326	mitogen-activated protein kinase kinase kinase 8	MAP3K8	upregulated
10150	muscleblind like splicing regulator 2	MBNL2	upregulated
2122	MDS1 and EVI1 complex locus	MECOM	upregulated
4224	meprin A subunit alpha	MEP1A	upregulated
23269	MGA, MAX dimerization protein	MGA	upregulated
10724	meningioma expressed antigen 5 (hyaluronidase)	MGEA5	upregulated
4256	matrix Gla protein	MGP	upregulated
83661	membrane spanning 4-domains A8	MS4A8	upregulated
4496	metallothionein 1H	MT1H	upregulated
4582	mucin 1, cell surface associated	MUC1	upregulated
10071	mucin 12, cell surface associated	MUC12	upregulated
140453	mucin 17, cell surface associated	MUC17	upregulated
4583	mucin 2, oligomeric mucus/gel-forming	MUC2	upregulated
4585	mucin 4, cell surface associated	MUC4	upregulated
4629	myosin heavy chain 11	MYH11	upregulated
10398	myosin light chain 9	MYL9	upregulated
4638	myosin light chain kinase	MYLK	upregulated
4646	myosin VI	MYO6	upregulated
93649	myocardin	MYOCD	upregulated
26509	myoferlin	MYOF	upregulated
4671	NLR family apoptosis inhibitory protein	NAIP	upregulated
4678	nuclear autoantigenic sperm protein	NASP	upregulated
55672	neuroblastoma breakpoint family member 1	NBPF1	upregulated
100132406	neuroblastoma breakpoint family member 10	NBPF10	upregulated
25832	neuroblastoma breakpoint family member 14	NBPF14	upregulated
101060684	neuroblastoma breakpoint family member 26	NBPF26	upregulated
400818	neuroblastoma breakpoint family member 9	NBPF9	upregulated
54820	nudE neurodevelopment protein 1	NDE1	upregulated
4714	NADH:ubiquinone oxidoreductase subunit B8	NDUFB8	upregulated
91624	nexilin F-actin binding protein	NEXN	upregulated
10725	nuclear factor of activated T-cells 5	NFAT5	upregulated
84901	nuclear factor of activated T-cells 2 interacting protein	NFATC2IP	upregulated

ENTEREZ_ID	GENE_NAME	GENE_SYMBOL	STATE
22795	nidogen 2	NID2	upregulated
388677	notch 2 N-terminal like	NOTCH2NL	upregulated
2494	nuclear receptor subfamily 5 group A member 2	NR5A2	upregulated
8829	neuropilin 1	NRP1	upregulated
8473	O-linked N-acetylglucosamine (GlcNAc) transferase	OGT	upregulated
10562	olfactomedin 4	OLFM4	upregulated
23022	palladin, cytoskeletal associated protein	PALLD	upregulated
167153	polyA) RNA polymerase D4, non-canonical	PAPD4	upregulated
79668	poly(ADP-ribose) polymerase family member 8	PARP8	upregulated
55742	parvin alpha	PARVA	upregulated
5125	proprotein convertase subtilisin/kexin type 5	PCSK5	upregulated
9659	phosphodiesterase 4D interacting protein	PDE4DIP	upregulated
8654	phosphodiesterase 5A	PDE5A	upregulated
5152	phosphodiesterase 9A	PDE9A	upregulated
27295	PDZ and LIM domain 3	PDLIM3	upregulated
10611	PDZ and LIM domain 5	PDLIM5	upregulated
57162	pellino E3 ubiquitin protein ligase 1	PELI1	upregulated
80162	protein-glucosylgalactosylhydroxylysine glucosidase	PGGHG	upregulated
9749	phosphatase and actin regulator 2	PHACTR2	upregulated
5266	peptidase inhibitor 3	PI3	upregulated
5284	polymeric immunoglobulin receptor	PIGR	upregulated
5286	phosphatidylinositol-4-phosphate 3-kinase catalytic subunit type 2 alpha	PIK3C2A	upregulated
8399	phospholipase A2 group X	PLA2G10	upregulated
5320	phospholipase A2 group IIA	PLA2G2A	upregulated
51316	placenta specific 8	PLAC8	upregulated
51196	phospholipase C epsilon 1	PLCE1	upregulated
5350	phospholamban	PLN	upregulated
25957	PNN interacting serine and arginine rich protein	PNISR	upregulated
5411	pinin, desmosome associated protein	PNN	upregulated
5368	prepronociceptin	PNOC	upregulated
10631	periostin	POSTN	upregulated
27068	pyrophosphatase (inorganic) 2	PPA2	upregulated
4659	protein phosphatase 1 regulatory subunit 12A	PPP1R12A	upregulated
5527	protein phosphatase 2 regulatory subunit B'gamma	PPP2R5C	upregulated
84366	prostate cancer susceptibility candidate 1	PRAC1	upregulated
55119	pre-mRNA processing factor 38B	PRPF38B	upregulated
26191	protein tyrosine phosphatase, non-receptor type 22	PTPN22	upregulated
22931	RAB18, member RAS oncogene family	RAB18	upregulated
10928	ralA binding protein 1	RALBP1	upregulated
158158	RAS and EF-hand domain containing	RASEF	upregulated
58517	RNA binding motif protein 25	RBM25	upregulated

ENTEREZ_ID	GENE_NAME	GENE_SYMBOL	STATE
10181	RNA binding motif protein 5	RBM5	upregulated
5937	RNA binding motif single stranded interacting protein 1	RBMS1	upregulated
5967	regenerating family member 1 alpha	REG1A	upregulated
5968	regenerating family member 1 beta	REG1B	upregulated
5068	regenerating family member 3 alpha	REG3A	upregulated
473	arginine-glutamic acid dipeptide repeats	RERE	upregulated
5996	regulator of G-protein signaling 1	RGS1	upregulated
5997	regulator of G-protein signaling 2	RGS2	upregulated
54933	rhomboid like 2	RHBDL2	upregulated
22836	Rho related BTB domain containing 3	RHOBTB3	upregulated
23433	ras homolog family member Q	RHOQ	upregulated
55183	replication timing regulatory factor 1	RIF1	upregulated
55599	RNA binding region (RNP1, RRM) containing 3	RNPC3	upregulated
6160	ribosomal protein L31	RPL31	upregulated
6232	ribosomal protein S27	RPS27	upregulated
6239	ras responsive element binding protein 1	RREB1	upregulated
51773	remodeling and spacing factor 1	RSF1	upregulated
6280	S100 calcium binding protein A9	S100A9	upregulated
9169	SR-related CTD associated factor 11	SCAF11	upregulated
57466	SR-related CTD associated factor 4	SCAF4	upregulated
9728	SECIS binding protein 2 like	SECISBP2L	upregulated
5269	serpin family B member 6	SERPINB6	upregulated
5054	serpin family E member 1	SERPINE1	upregulated
710	serpin family G member 1	SERPING1	upregulated
2810	stratifin	SFN	upregulated
6423	secreted frizzled related protein 2	SFRP2	upregulated
30011	SH3 domain containing kinase binding protein 1	SH3KBP1	upregulated
6476	sucrase-isomaltase	SI	upregulated
57606	SLAIN motif family member 2	SLAIN2	upregulated
1836	solute carrier family 26 member 2	SLC26A2	upregulated
1811	solute carrier family 26 member 3	SLC26A3	upregulated
9153	solute carrier family 28 member 2	SLC28A2	upregulated
151258	solute carrier family 38 member 11	SLC38A11	upregulated
6590	secretory leukocyte peptidase inhibitor	SLPI	upregulated
128710	SLX4 interacting protein	SLX4IP	upregulated
9126	structural maintenance of chromosomes 3	SMC3	upregulated
23347	structural maintenance of chromosomes flexible hinge domain containing 1	SMCHD1	upregulated
201895	small integral membrane protein 14	SMIM14	upregulated
284422	small integral membrane protein 24	SMIM24	upregulated
64750	SMAD specific E3 ubiquitin protein ligase 2	SMURF2	upregulated

ENTEREZ_ID	GENE_NAME	GENE_SYMBOL	STATE
9043	sperm associated antigen 9	SPAG9	upregulated
8404	SPARC like 1	SPARCL1	upregulated
23013	spen family transcriptional repressor	SPEN	upregulated
6690	serine peptidase inhibitor, Kazal type 1	SPINK1	upregulated
57522	SLIT-ROBO Rho GTPase activating protein 1	SRGAP1	upregulated
6733	SRSF protein kinase 2	SRPK2	upregulated
27286	sushi repeat containing protein, X-linked 2	SRPX2	upregulated
23524	serine/arginine repetitive matrix 2	SRRM2	upregulated
6429	serine and arginine rich splicing factor 4	SRSF4	upregulated
27284	sulfotransferase family 1B member 1	SULT1B1	upregulated
23336	synemin	SYNM	upregulated
6867	transforming acidic coiled-coil containing protein 1	TACC1	upregulated
8148	TATA-box binding protein associated factor 15	TAF15	upregulated
79101	TATA-box binding protein associated factor, RNA polymerase I subunit D	TAF1D	upregulated
6876	transgelin	TAGLN	upregulated
79718	transducin beta like 1 X-linked receptor 1	TBL1XR1	upregulated
347853	T-box 10	TBX10	upregulated
51186	transcription elongation factor A like 9	TCEAL9	upregulated
54881	testis expressed 10	TEX10	upregulated
7046	transforming growth factor beta receptor 1	TGFBR1	upregulated
83941	TM2 domain containing 1	TM2D1	upregulated
79853	transmembrane 4 L six family member 20	TM4SF20	upregulated
7104	transmembrane 4 L six family member 4	TM4SF4	upregulated
79838	transmembrane channel like 5	TMC5	upregulated
7110	TATA element modulatory factor 1	TMF1	upregulated
7113	transmembrane protease, serine 2	TMPRSS2	upregulated
3371	tenascin C	TNC	upregulated
23043	TRAF2 and NCK interacting kinase	TNIK	upregulated
7145	tensin 1	TNS1	upregulated
10140	transducer of ERBB2, 1	TOB1	upregulated
7150	topoisomerase (DNA) I	TOP1	upregulated
7153	topoisomerase (DNA) II alpha	TOP2A	upregulated
9878	TOX high mobility group box family member 4	TOX4	upregulated
28738	T cell receptor alpha joining 17	TRAJ17	upregulated
66008	trafficking kinesin protein 2	TRAK2	upregulated
57616	teashirt zinc finger homeobox 3	TSHZ3	upregulated
10103	tetraspanin 1	TSPAN1	upregulated
151613	tetratricopeptide repeat domain 14	TTC14	upregulated
55075	uveal autoantigen with coiled-coil domains and ankyrin repeats	UACA	upregulated
26043	UBX domain protein 7	UBXN7	upregulated

ENTEREZ_ID	GENE_NAME	GENE_SYMBOL	STATE
7367	UDP glucuronosyltransferase family 2 member B17	UGT2B17	upregulated
29761	ubiquitin specific peptidase 25	USP25	upregulated
84196	ubiquitin specific peptidase 48	USP48	upregulated
8239	ubiquitin specific peptidase 9, X-linked	USP9X	upregulated
81671	vacuole membrane protein 1	VMP1	upregulated
157680	vacuolar protein sorting 13 homolog B	VPS13B	upregulated
10163	WAS protein family member 2	WASF2	upregulated
55717	WD repeat domain 11	WDR11	upregulated
79971	wntless Wnt ligand secretion mediator	WLS	upregulated
65125	WNK lysine deficient protein kinase 1	WNK1	upregulated
26118	WD repeat and SOCS box containing 1	WSB1	upregulated
26137	zinc finger and BTB domain containing 20	ZBTB20	upregulated
79670	zinc finger CCHC-type containing 6	ZCCHC6	upregulated
84186	zinc finger CCHC-type containing 7	ZCCHC7	upregulated
80829	ZFP91 zinc finger protein	ZFP91	upregulated
7750	zinc finger MYM-type containing 2	ZMYM2	upregulated
23613	zinc finger MYND-type containing 8	ZMYND8	upregulated
51351	zinc finger protein 117	ZNF117	upregulated
100129482	zinc finger protein 37B, pseudogene	ZNF37BP	upregulated
55205	zinc finger protein 532	ZNF532	upregulated
152687	zinc finger protein 595	ZNF595	upregulated
374860	Ankyrin repeat domain 30B	ANKRD30B	downregulated
1066	Carboxylesterase 1	CES1	downregulated
10877	Complement factor H related 4	CFHR4	downregulated
84766	Calcium release activated channel regulator 2A	CRACR2A	downregulated
6373	C-X-C motif chemokine ligand 11	CXCL11	downregulated
3576	C-X-C motif chemokine ligand 8	CXCL8	downregulated
3502	Immunoglobulin heavy constant gamma 3	G3m marker	downregulated
729396	G antigen 12J	GAGE12J	downregulated
2574	G antigen 2C	GAGE2C	downregulated
2543	G antigen 1	GAGE1	downregulated
2576	G antigen 4	GAGE4	downregulated
100101629	G antigen 8	GAGE8	downregulated
2747	glutamate dehydrogenase 2	GLUD2	downregulated
3119	Major histocompatibility complex, class II, DQ beta 1	HLA-DQB1	downregulated
3284	hydroxy-delta-5-steroid dehydrogenase, 3 beta- and steroid delta-isomerase 2	HSD3B2	downregulated
3493	Immunoglobulin heavy constant alpha 1	IGHA1	downregulated
3495	Immunoglobulin heavy constant delta	IGHD	downregulated
3507	Immunoglobulin heavy constant mu	IGHM	downregulated
28461	Immunoglobulin heavy variable 1-69	IGHV1-69	downregulated
28396	Immunoglobulin heavy variable 4-31	IGHV4-31	downregulated

ENTEREZ_ID	GENE_NAME	GENE_SYMBOL	STATE
50802	Immunoglobulin kappa locus	IGK	downregulated
3514	Immunoglobulin kappa constant	IGKC	downregulated
3537	Immunoglobulin lambda constant 1	IGLC1	downregulated
3552	Interleukin 1 alpha	IL1A	downregulated
3553	Interleukin 1 beta	IL1B	downregulated
3768	potassium voltage-gated channel subfamily J member 12	KCNJ12	downregulated
8549	Leucine rich repeat containing G protein-coupled receptor 5	LGR5	downregulated
374819	Leucine rich repeat containing 37 member A3	LRRC37A3	downregulated
100507027	myoregulin	MRLN	downregulated
55154	misato 1, mitochondrial distribution and morphology regulator	MSTO1	downregulated
645682	POU class 5 homeobox 1 pseudogene 4	POU5F1P4	downregulated
5690	proteasome subunit beta 2	PSMB2	downregulated
5789	protein tyrosine phosphatase, receptor type D	PTPRD	downregulated
6004	regulator of G-protein signaling 16	RGS16	downregulated
6256	retinoid X receptor alpha	RXRA	downregulated
221833	Sp8 transcription factor	SP8	downregulated
10732	transcription factor like 5	TCFL5	downregulated
80304	WD repeat and coiled coil containing	WDCP	downregulated
10730	YME1 like 1 ATPase	YME1L1	downregulated

Table S9. Available clinical data of patients treated with 5-FU-based therapies. Relationship between recurrence condition and available clinicopathological features from patients treated with 5-FU, FOLFIRI or FOLFLOX selected for merged matrix construction.

Variables	Cases (n)	Recurrence		*p* value
		No	Yes	(fisher test)
		**GSE39582**		
Total	117	74	43	
Treatment				
5-FU	82	53	29	0.73
FOLFLOX	23	12	11	
FOLFIRI	12	9	3	
Age (years)				
Mean	63.23	64.26	63.21	0.33
<60	45	31	14	
>=60	72	43	29	
TNM stage				
II	16	13	3	0.16
III/IV	101	61	40	
T stage				
T2	8	6	2	0.84
T3/T4	109	68	41	
N stage				
Negative	23	14	9	0.81
Positive	94	60	34	
M stage [a]				
Negative	93	59	34	0.87
Positive	23	15	8	
tp53 Mutation [b]				
M	18	13	5	0.71
WT	14	9	5	
kras Mutation [c]				
M	44	25	19	0.14
WT	57	41	16	
braf Mutation [d]				
M	7	5	2	0.8
WT	91	61	30	
		**GSE72970**		
Total	92	47	45	
Treatment				

FOLFIRI	60	27	33	
FOLFLOX	32	20	12	
Age (years)				
Mean	62.74	63.7	61.8	0.28
<60	37	16	21	
>=60	55	31	24	
T stage [e]				
T1/T2	8	4	4	0.87
T3/T4	63	34	29	
M stage				
Negative	15	7	8	0.78
Positive	77	40	37	
Location				
Caecum	3	2	1	0.32
Left colon	41	25	16	
Rectum	15	6	9	
Rectum-sigmoid junction	12	7	5	
Right colon	20	7	13	

a One case wasn't able to characterize b 85 cases were NA

c 16 cases were NA d 19 cases were NA

Not available public clinical information for dataset GSE81653(n=358)

Table S10. GSEA analysis between 5-FU, FOLFIRI and FOLFLOX treated recurrent and non-recurrent phenotypes for the merged matrix. Output table of the GSEA analysis between 5-FU, FOLFIRI and FOLFLOX treated recurrent and non-recurrent phenotypes for the merged matrix. To perform the analysis the gene sets contained in c2.cp.reactome.v2023.1.Hs.symbols.gmt, c5.go.bp.v2023.1.Hs.symbols.gmt, c5.go.mf.v2023.1.Hs.symbols.gmt, c5.go.cc.v2023.1.Hs.symbols.gmt and c2.cp.kegg.v2023.1. Hs.symbols.gmt databases were used. Only enriched gene sets with an FDRq-val<0.05 are shown.

GEN SET NAME	ENRICHMENT IN PHENOTYPE	SIZE	ES	NES	NOM p-val	FDR q-val	FWER p-val	RANK AT MAX	LEADING EDGE
GOBP_CELLULAR_GLUCURONIDATION	Recurrent	18	0,83	2,37	0,000	0,000	0,000	452	tags=56%, list=2%, signal=57%
REACTOME_GLUCURONIDATION	Recurrent	21	0,78	2,33	0,000	0,000	0,001	452	tags=48%, list=2%, signal=49%
KEGG_DRUG_METABOLISM_OTHER_ ENZYMES	Recurrent	48	0,65	2,36	0,000	0,001	0,001	452	tags=31%, list=2%, signal=32%
GOBP_URONIC_ACID_METABOLIC_ PROCESS	Recurrent	23	0,74	2,27	0,000	0,001	0,003	452	tags=43%, list=2%, signal=44%
KEGG_ASCORBATE_AND_ALDARATE_METABOLISM	Recurrent	22	0,75	2,27	0,000	0,001	0,003	452	tags=45%, list=2%, signal=46%
KEGG_PENTOSE_AND_GLUCURONATE_INTERCONVERSIONS	Recurrent	25	0,7	2,20	0,000	0,002	0,008	452	tags=40%, list=2%, signal=41%
GOBP_NEGATIVE_REGULATION_OF_ MEGAKARYOCYTE_DIFFERENTIATION	Recurrent	17	0,77	2,15	0,000	0,004	0,025	3074	tags=59%, list=15%, signal=69%
REACTOME_RHOQ_GTPASE_CYCLE	Recurrent	59	0,57	2,12	0,000	0,009	0,058	5830	tags=56%, list=29%, signal=79%
REACTOME_RHO_GTPASES_ACTIVATE_PKNS	Recurrent	86	0,52	2,09	0,000	0,011	0,079	4028	tags=43%, list=20%, signal=54%
GOMF_GLUCURONOSYLTRANSFERASE_ACTIVITY	Recurrent	31	0,63	2,08	0,000	0,011	0,106	452	tags=32%, list=2%, signal=33%
REACTOME_HDACS_DEACETYLATE_ HISTONES	Recurrent	84	0,52	2,08	0,000	0,011	0,112	4065	tags=45%, list=20%, signal=56%

GEN SET NAME	ENRICHMENT IN PHENOTYPE	SIZE	ES	NES	NOM p-val	FDR q-val	FWER p-val	RANK AT MAX	LEADING EDGE
**REACTOME_FORMATION_OF_THE_ BETA_CATENIN_TCF_ TRANSACTIVATING_COMPLEX**	Recurrent	84	0,52	2,07	0,000	0,011	0,126	4327	tags=50%, list=22%, signal=63%
**GOBP_REGULATION_OF_ MEGAKARYOCYTE_DIFFERENTIATION**	Recurrent	35	0,61	2,05	0,000	0,011	0,158	3074	tags=46%, list=15%, signal=54%
**REACTOME_DEFECTIVE_C1GALT1C1_ CAUSES_TNPS**	Recurrent	16	0,75	2,05	0,000	0,011	0,158	2353	tags=56%, list=12%, signal=64%
**REACTOME_DEFECTIVE_GALNT3_ CAUSES_HFTC**	Recurrent	15	0,77	2,09	0,000	0,012	0,100	2353	tags=60%, list=12%, signal=68%
**GOBP_RESPONSE_TO_PLATELET_ DERIVED_GROWTH_FACTOR**	Recurrent	24	0,66	2,08	0,000	0,012	0,106	2617	tags=50%, list=13%, signal=57%
**REACTOME_TERMINATION_OF_O_ GLYCAN_BIOSYNTHESIS**	Recurrent	22	0,69	2,06	0,000	0,012	0,146	2353	tags=45%, list=12%, signal=51%
**GOBP_MICROTUBULE_ORGANIZING_ CENTER_LOCALIZATION**	Recurrent	33	0,62	2,06	0,000	0,012	0,152	3185	tags=42%, list=16%, signal=50%
**GOCC_MICROVILLUS_MEMBRANE**	Recurrent	27	0,65	2,05	0,000	0,012	0,157	2843	tags=44%, list=14%, signal=52%
**GOBP_NUCLEOSOME_ORGANIZATION**	Recurrent	136	0,47	2,04	0,000	0,012	0,184	4327	tags=43%, list=22%, signal=54%
**REACTOME_HCMV_EARLY_EVENTS**	Recurrent	125	0,48	2,04	0,000	0,012	0,198	4400	tags=44%, list=22%, signal=56%
**GOBP_MICROVILLUS_ORGANIZATION**	Recurrent	30	0,61	2,04	0,000	0,012	0,200	4915	tags=67%, list=25%, signal=88%
**GOMF_EPHRIN_RECEPTOR_BINDING**	Recurrent	29	0,62	2,04	0,000	0,012	0,204	5425	tags=62%, list=27%, signal=85%
**GOMF_CADHERIN_BINDING**	Recurrent	333	0,42	2,03	0,000	0,012	0,213	6785	tags=51%, list=34%, signal=76%

GEN SET NAME	ENRICHMENT IN PHENOTYPE	SIZE	ES	NES	NOM p-val	FDR q-val	FWER p-val	RANK AT MAX	LEADING EDGE
KEGG_STARCH_AND_SUCROSE_ METABOLISM	Recurrent	49	0,55	2,02	0,000	0,013	0,240	1026	tags=31%, list=5%, signal=32%
GOBP_BASE_EXCISION_REPAIR	Recurrent	43	0,57	2,02	0,000	0,013	0,248	6747	tags=70%, list=34%, signal=105%
GOBP_MICROVILLUS_ASSEMBLY	Recurrent	22	0,66	2,01	0,000	0,013	0,263	4915	tags=73%, list=25%, signal=96%
GOBP_NUCLEOSOME_ASSEMBLY	Recurrent	101	0,49	2,01	0,000	0,013	0,264	4327	tags=44%, list=22%, signal=55%
GOCC_INTEGRATOR_COMPLEX	Recurrent	27	0,63	2,02	0,002	0,014	0,261	4926	tags=67%, list=25%, signal=88%
REACTOME_HCMV_INFECTION	Recurrent	149	0,46	2,00	0,000	0,016	0,354	4605	tags=43%, list=23%, signal=55%
REACTOME_SIRT1_NEGATIVELY_ REGULATES_RRNA_EXPRESSION	Recurrent	60	0,52	2,00	0,000	0,017	0,347	4028	tags=47%, list=20%, signal=58%
REACTOME_SENESCENCE_ ASSOCIATED_SECRETORY_ PHENOTYPE_SASP	Recurrent	104	0,47	1,99	0,000	0,018	0,393	5796	tags=54%, list=29%, signal=75%
GOBP_PROTEIN_LOCALIZATION_TO_ CELL_CELL_JUNCTION	Recurrent	21	0,67	1,98	0,000	0,019	0,411	4976	tags=71%, list=25%, signal=95%
GOBP_MORPHOGENESIS_OF_AN_ EPITHELIAL_SHEET	Recurrent	59	0,53	1,98	0,000	0,019	0,426	3891	tags=39%, list=19%, signal=48%
REACTOME_ERCC6_CSB_AND_ EHMT2_G9A_POSITIVELY_REGULATE_RRNA_ EXPRESSION	Recurrent	68	0,51	1,97	0,000	0,019	0,436	4028	tags=46%, list=20%, signal=57%
REACTOME_PRC2_METHYLATES_ HISTONES_AND_DNA	Recurrent	65	0,51	1,97	0,000	0,019	0,438	4028	tags=48%, list=20%, signal=59%
REACTOME_CHROMATIN_MODIFYING_ ENZYMES	Recurrent	263	0,42	1,97	0,000	0,019	0,447	5158	tags=42%, list=26%, signal=56%

GEN SET NAME	ENRICHMENT IN PHENOTYPE	SIZE	ES	NES	NOM p-val	FDR q-val	FWER p-val	RANK AT MAX	LEADING EDGE
GOMF_RETINOIC_ACID_BINDING	Recurrent	25	0,63	1,97	0,000	0,019	0,467	452	tags=32%, list=2%, signal=33%
KEGG_PORPHYRIN_AND_CHLOROPHYLL_METABOLISM	Recurrent	37	0,57	1,97	0,000	0,020	0,466	1154	tags=32%, list=6%, signal=34%
REACTOME_CONDENSATION_OF_PROPHASE_CHROMOSOMES	Recurrent	66	0,51	1,96	0,000	0,023	0,541	4028	tags=45%, list=20%, signal=57%
REACTOME_ACTIVATED_PKN1_STIMULATES_TRANSCRIPTION_OF_AR_ANDROGEN_RECEPTOR_REGULATED_GENES_KLK2_AND_KLK3	Recurrent	59	0,52	1,95	0,002	0,023	0,551	4028	tags=44%, list=20%, signal=55%
REACTOME_ASSEMBLY_OF_THE_ORC_COMPLEX_AT_THE_ORIGIN_OF_REPLICATION	Recurrent	61	0,52	1,95	0,000	0,023	0,565	4327	tags=48%, list=22%, signal=60%
GOBP_POSITIVE_REGULATION_OF_CYCLIN_DEPENDENT_PROTEIN_KINASE_ACTIVITY	Recurrent	39	0,57	1,94	0,000	0,026	0,618	2884	tags=44%, list=14%, signal=51%
GOBP_DNA_REPLICATION_DEPENDENT_CHROMATIN_ASSEMBLY	Recurrent	30	0,59	1,94	0,000	0,026	0,635	4007	tags=60%, list=20%, signal=75%
GOBP_REGULATION_OF_AUTOPHAGY_OF_MITOCHONDRION	Recurrent	36	0,57	1,94	0,000	0,026	0,652	5438	tags=64%, list=27%, signal=87%
REACTOME_DNA_REPAIR	Recurrent	328	0,4	1,94	0,000	0,027	0,646	7071	tags=51%, list=35%, signal=78%
REACTOME_RUNX1_REGULATES_GENES_INVOLVED_IN_MEGAKARYOCYTE_DIFFERENTIATION_AND_PLATELET_FUNCTION	Recurrent	86	0,48	1,92	0,000	0,028	0,730	4913	tags=50%, list=24%, signal=66%
GOMF_BETA_CATENIN_BINDING	Recurrent	88	0,48	1,93	0,000	0,029	0,706	5051	tags=48%, list=25%, signal=64%

GEN SET NAME	ENRICHMENT IN PHENOTYPE	SIZE	ES	NES	NOM p-val	FDR q-val	FWER p-val	RANK AT MAX	LEADING EDGE
REACTOME_HDMS_DEMETHYLATE_ HISTONES	Recurrent	45	0,53	1,93	0,000	0,029	0,715	4007	tags=49%, list=20%, signal=61%
REACTOME_SPHINGOLIPID_DE_NOVO_BIOSYNTHESIS	Recurrent	44	0,54	1,93	0,002	0,029	0,719	5654	tags=64%, list=28%, signal=88%
REACTOME_DNA_METHYLATION	Recurrent	57	0,51	1,92	0,000	0,029	0,730	4028	tags=47%, list=20%, signal=59%
REACTOME_PKMTS_METHYLATE_ HISTONE_LYSINES	Recurrent	66	0,5	1,92	0,000	0,029	0,748	5057	tags=53%, list=25%, signal=71%
REACTOME_DECTIN_2_FAMILY	Recurrent	24	0,62	1,92	0,000	0,029	0,756	2353	tags=42%, list=12%, signal=47%
REACTOME_OXIDATIVE_STRESS_ INDUCED_SENESCENCE	Recurrent	113	0,46	1,93	0,000	0,030	0,714	4768	tags=46%, list=24%, signal=60%
REACTOME_RHOJ_GTPASE_CYCLE	Recurrent	55	0,52	1,92	0,000	0,030	0,765	5830	tags=49%, list=29%, signal=69%
REACTOME_SPHINGOLIPID_ METABOLISM	Recurrent	87	0,48	1,91	0,000	0,031	0,776	5654	tags=53%, list=28%, signal=73%
GOBP_POSITIVE_REGULATION_OF_PROTEASOMAL_UBIQUITIN_DEPENDENT_ PROTEIN_CATABOLIC_PROCESS	Recurrent	95	0,46	1,91	0,000	0,031	0,786	3078	tags=36%, list=15%, signal=42%
REACTOME_RND2_GTPASE_CYCLE	Recurrent	43	0,53	1,91	0,000	0,031	0,796	5763	tags=53%, list=29%, signal=75%
REACTOME_NEGATIVE_REGULATION_ OF_MAPK_PATHWAY	Recurrent	43	0,55	1,91	0,000	0,031	0,802	4594	tags=49%, list=23%, signal=63%
GOBP_REGULATION_OF_UBIQUITIN_ PROTEIN_TRANSFERASE_ACTIVITY	Recurrent	56	0,5	1,89	0,000	0,033	0,879	6382	tags=50%, list=32%, signal=73%

GEN SET NAME	ENRICHMENT IN PHENOTYPE	SIZE	ES	NES	NOM p-val	FDR q-val	FWER p-val	RANK AT MAX	LEADING EDGE
REACTOME_BASE_EXCISION_REPAIR_AP_SITE_FORMATION	Recurrent	58	0,5	1,89	0,000	0,033	0,880	4327	tags=43%, list=22%, signal=55%
GOMF_SNAP_RECEPTOR_ACTIVITY	Recurrent	37	0,55	1,89	0,000	0,033	0,882	6072	tags=62%, list=30%, signal=89%
GOCC_TRANSCRIPTION_ELONGATION_FACTOR_COMPLEX	Recurrent	47	0,52	1,90	0,000	0,034	0,835	6421	tags=51%, list=32%, signal=75%
REACTOME_BASE_EXCISION_REPAIR	Recurrent	86	0,46	1,90	0,000	0,034	0,842	6240	tags=53%, list=31%, signal=77%
REACTOME_REGULATION_OF_PLK1_ ACTIVITY_AT_G2_M_TRANSITION	Recurrent	87	0,48	1,90	0,000	0,034	0,843	6788	tags=57%, list=34%, signal=86%
REACTOME_COPI_INDEPENDENT_ GOLGI_TO_ER_RETROGRADE_TRAFFIC	Recurrent	50	0,51	1,90	0,000	0,034	0,855	3651	tags=42%, list=18%, signal=51%
GOBP_NEPHRIC_DUCT_DEVELOPMENT	Recurrent	16	0,69	1,89	0,002	0,034	0,871	824	tags=31%, list=4%, signal=33%
REACTOME_DEPOSITION_OF_NEW_ CENPA_CONTAINING_NUCLEOSOMES_AT_THE_CENTROMERE	Recurrent	68	0,49	1,89	0,000	0,034	0,871	4028	tags=43%, list=20%, signal=53%
GOCC_PML_BODY	Recurrent	105	0,45	1,89	0,000	0,034	0,875	5000	tags=42%, list=25%, signal=56%
GOBP_MICROTUBULE_ANCHORING	Recurrent	26	0,59	1,89	0,000	0,034	0,877	6352	tags=73%, list=32%, signal=107%
REACTOME_RHOF_GTPASE_CYCLE	Recurrent	41	0,54	1,89	0,003	0,034	0,878	4847	tags=41%, list=24%, signal=55%
REACTOME_GLOBAL_GENOME_ _ NUCLEOTIDE_EXCISION_REPAIR_GG_	Recurrent	84	0,47	1,88	0,000	0,034	0,892	7568	tags=60%, list=38%, signal=95%

GEN SET NAME	ENRICHMENT IN PHENOTYPE	SIZE	ES	NES	NOM p-val	FDR q-val	FWER p-val	RANK AT MAX	LEADING EDGE
NER									
REACTOME_PROTEIN_UBIQUITINATION	Recurrent	78	0,48	1,89	0,002	0,035	0,871	6641	tags=55%, list=33%, signal=82%
GOBP_AUTOPHAGY_OF_ MITOCHONDRION	Recurrent	93	0,46	1,89	0,000	0,035	0,871	6203	tags=52%, list=31%, signal=74%
KEGG_ADHERENS_JUNCTION	Recurrent	73	0,47	1,88	0,000	0,036	0,907	5311	tags=45%, list=26%, signal=61%
KEGG_INSULIN_SIGNALING_PATHWAY	Recurrent	136	0,43	1,87	0,000	0,036	0,912	5838	tags=45%, list=29%, signal=63%
GOCC_ADHERENS_JUNCTION	Recurrent	179	0,42	1,87	0,000	0,036	0,913	3475	tags=32%, list=17%, signal=39%
REACTOME_NONHOMOLOGOUS_END_ JOINING_NHEJ	Recurrent	64	0,49	1,88	0,000	0,037	0,904	3225	tags=36%, list=16%, signal=43%
GOMF_CYCLIN_DEPENDENT_PROTEIN_SERINE_THREONINE_ KINASE_ REGULATOR_ACTIVITY	Recurrent	47	0,51	1,88	0,000	0,037	0,905	6013	tags=60%, list=30%, signal=85%
GOBP_INTRINSIC_APOPTOTIC_ SIGNALING_PATHWAY_IN_RESPONSE_TO_DNA_DAMAGE_BY_ P53_CLASS_ MEDIATOR	Recurrent	43	0,53	1,88	0,002	0,037	0,906	5067	tags=51%, list=25%, signal=68%
GOBP_SNRNA_PROCESSING	Recurrent	36	0,55	1,87	0,002	0,039	0,925	4926	tags=50%, list=25%, signal=66%
GOBP_POSITIVE_REGULATION _OF_P38MAPK_CASCADE	Recurrent	27	0,59	1,86	0,000	0,039	0,929	4653	tags=52%, list=23%, signal=67%
REACTOME_SIGNALING_BY_WNT_IN_ CANCER	Recurrent	33	0,55	1,86	0,000	0,042	0,944	7378	tags=64%, list=37%, signal=100%
REACTOME_RMTS_METHYLATE_ HISTONE_ARGININES	Recurrent	71	0,48	1,86	0,000	0,043	0,944	4028	tags=45%, list=20%, signal=56%

GEN SET NAME	ENRICHMENT IN PHENOTYPE	SIZE	ES	NES	NOM p-val	FDR q-val	FWER p-val	RANK AT MAX	LEADING EDGE
REACTOME_MITOTIC_G2_G2_M_ _ PHASES	Recurrent	196	0,41	1,86	0,000	0,043	0,947	6809	tags=50%, list=34%, signal=75%
REACTOME_ESTROGEN_DEPENDENT_ GENE_EXPRESSION	Recurrent	138	0,43	1,85	0,000	0,043	0,949	4925	tags=41%, list=25%, signal=53%
GOBP_REGULATION_OF_ENDOTHELIAL_CELL_DIFFERENTIATION	Recurrent	39	0,54	1,85	0,002	0,044	0,956	3213	tags=41%, list=16%, signal=49%
REACTOME_ACTIVATION_OF_ ANTERIOR_HOX_GENES_IN_HINDBRAIN_DEVELOPMENT_DURING_EARLY_ EMBRYOGENESIS	Recurrent	114	0,44	1,85	0,000	0,045	0,960	4776	tags=42%, list=24%, signal=55%
REACTOME_TBC_RABGAPS	Recurrent	44	0,51	1,85	0,000	0,045	0,960	7778	tags=66%, list=39%, signal=107%
GOCC_ACTIN_CYTOSKELETON	Recurrent	494	0,37	1,84	0,000	0,046	0,963	6212	tags=44%, list=31%, signal=63%
REACTOME_E3_UBIQUITIN_LIGASES_ UBIQUITINATE_TARGET_PROTEINS	Recurrent	58	0,49	1,84	0,000	0,046	0,964	6352	tags=53%, list=32%, signal=78%
GOMF_STRUCTURAL_CONSTITUENT_ OF_CYTOSKELETON	Recurrent	102	0,44	1,84	0,000	0,047	0,968	4853	tags=42%, list=24%, signal=55%
REACTOME_TRANSCRIPTIONAL_ REGULATION_OF_GRANULOPOIESIS	Recurrent	82	0,46	1,84	0,000	0,047	0,971	4340	tags=41%, list=22%, signal=53%
GOMF_STRUCTURAL_CONSTITUENT_ OF_CHROMATIN	Recurrent	76	0,46	1,84	0,000	0,047	0,972	4028	tags=41%, list=20%, signal=51%
GOCC_CYCLIN_DEPENDENT_PROTEIN_KINASE_HOLOENZYME_COMPLEX	Recurrent	49	0,5	1,84	0,000	0,048	0,975	6051	tags=57%, list=30%, signal=82%
GOBP_PEPTIDYL_TYROSINE_ AUTOPHOSPHORYLATION	Recurrent	21	0,62	1,84	0,000	0,049	0,975	3305	tags=52%, list=16%, signal=63%

GEN SET NAME	ENRICHMENT IN PHENOTYPE	SIZE	ES	NES	NOM p-val	FDR q-val	FWER p-val	RANK AT MAX	LEADING EDGE
GOBP_POSITIVE_REGULATION_OF_DNA_REPLICATION	Recurrent	40	0,53	1,83	0,000	0,049	0,976	6051	tags=60%, list=30%, signal=86%
REACTOME_RHO_GTPASE_EFFECTORS	Recurrent	313	0,38	1,83	0,000	0,049	0,978	5827	tags=44%, list=29%, signal=61%
GOBP_ORGANELLE_DISASSEMBLY	Recurrent	139	0,42	1,83	0,000	0,049	0,978	6483	tags=47%, list=32%, signal=70%
REACTOME_NUCLEAR_EVENTS_ KINASE_AND_TRANSCRIPTION_ FACTOR_ACTIVATION	Recurrent	61	0,48	1,83	0,000	0,050	0,981	4795	tags=44%, list=24%, signal=58%
GOBP_POSITIVE_REGULATION_OF_ UBIQUITIN_PROTEIN_TRANSFERASE_ ACTIVITY	Recurrent	32	0,56	1,83	0,000	0,050	0,982	3900	tags=41%, list=19%, signal=50%
GOMF_OLFACTORY_RECEPTOR_ ACTIVITY	Not recurrent	92	-0,6	-2,67	0,000	0,000	0,000	4291	tags=58%, list=21%, signal=73%
REACTOME_OLFACTORY_SIGNALING_ PATHWAY	Not recurrent	102	-0,55	-2,49	0,000	0,000	0,000	3354	tags=47%, list=17%, signal=56%
GOMF_GPRC_LIGAND_BINDING	Not recurrent	22	-0,77	-2,46	0,000	0,000	0,000	2063	tags=55%, list=10%, signal=61%
GOBP_SENSORY_PERCEPTION_OF_ SMELL	Not recurrent	117	-0,53	-2,46	0,000	0,000	0,000	3354	tags=43%, list=17%, signal=51%
GOBP_DETECTION_OF_STIMULUS_ INVOLVED_IN_SENSORY_PERCEPTION	Not recurrent	201	-0,47	-2,46	0,000	0,000	0,003	2410	tags=32%, list=12%, signal=36%
REACTOME_SCAVENGING_BY_CLASS_A_RECEPTORS	Not recurrent	19	-0,72	-2,32	0,000	0,001	0,006	1549	tags=53%, list=8%, signal=57%
REACTOME_IMMUNOREGULATORY_ INTERACTIONS_BETWEEN_A_	Not recurrent	123	-0,49	-2,28	0,000	0,001	0,011	4038	tags=48%, list=20%, signal=60%

GEN SET NAME	ENRICHMENT IN PHENOTYPE	SIZE	ES	NES	NOM p-val	FDR q-val	FWER p-val	RANK AT MAX	LEADING EDGE
LYMPHOID_AND_A_NON_LYMP HOID_ CELL									
GOBP_SENSORY_PERCEPTION _OF_ CHEMICAL_STIMULUS	Not recurrent	187	-0,45	-2,25	0,000	0,002	0,023	3680	tags=41%, list=18%, signal=50%
GOBP_SEROTONIN_TRANSPOR T	Not recurrent	19	-0,69	-2,19	0,000	0,004	0,072	1226	tags=26%, list=6%, signal=28%
KEGG_COMPLEMENT_AND_ COAGULATION_CASCADES	Not recurrent	69	-0,52	-2,18	0,000	0,004	0,076	2496	tags=35%, list=12%, signal=40%
GOBP_REGULATION_OF_PEPTI DYL_ SERINE_PHOSPHORYLATION_ OF_ STAT_PROTEIN	Not recurrent	22	-0,68	-2,18	0,000	0,004	0,077	884	tags=36%, list=4%, signal=38%
GOMF_EXTRACELLULAR_MAT RIX_STRUCTURAL_CONSTITUE NT_CONFERRING_COMPRESSI ON_RESISTANCE	Not recurrent	22	-0,66	-2,19	0,000	0,005	0,066	1541	tags=45%, list=8%, signal=49%
GOBP_DETECTION_OF_CHEMI CAL_ STIMULUS	Not recurrent	169	-0,45	-2,19	0,000	0,005	0,069	3575	tags=41%, list=18%, signal=49%
GOCC_HIGH_DENSITY_LIPOPR OTEIN_ PARTICLE	Not recurrent	27	-0,63	-2,16	0,000	0,005	0,108	2521	tags=48%, list=13%, signal=55%
GOBP_COMPLEMENT_ACTIVAT ION	Not recurrent	69	-0,51	-2,15	0,000	0,006	0,121	3252	tags=45%, list=16%, signal=53%
KEGG_OLFACTORY_TRANSDU CTION	Not recurrent	118	-0,46	-2,13	0,000	0,006	0,146	4291	tags=49%, list=21%, signal=62%
REACTOME_COMPLEMENT_CA SCADE	Not recurrent	60	-0,52	-2,12	0,000	0,007	0,169	3672	tags=47%, list=18%, signal=57%
GOMF_TYPE_I_INTERFERON_ RECEPTOR_BINDING	Not recurrent	16	-0,71	-2,11	0,000	0,007	0,184	884	tags=44%, list=4%, signal=46%

GEN SET NAME	ENRICHMENT IN PHENOTYPE	SIZE	ES	NES	NOM p-val	FDR q-val	FWER p-val	RANK AT MAX	LEADING EDGE
GOBP_PHAGOCYTOSIS_RECOGNITION	Not recurrent	42	-0,57	-2,11	0,000	0,007	0,205	2160	tags=36%, list=11%, signal=40%
GOBP_SERINE_PHOSPHORYLATION_OF_STAT_PROTEIN	Not recurrent	26	-0,62	-2,11	0,003	0,008	0,196	884	tags=31%, list=4%, signal=32%
GOBP_COLLAGEN_FIBRIL_ ORGANIZATION	Not recurrent	63	-0,51	-2,09	0,000	0,009	0,241	1601	tags=25%, list=8%, signal=28%
GOBP_PROTEIN_ACTIVATION_ CASCADE	Not recurrent	20	-0,65	-2,06	0,000	0,012	0,333	1012	tags=30%, list=5%, signal=32%
GOMF_NADPLUS_NUCLEOSIDASE_ ACTIVITY	Not recurrent	16	-0,68	-2,06	0,000	0,013	0,358	3606	tags=44%, list=18%, signal=53%
GOMF_G_PROTEIN_COUPLED_ CHEMOATTRACTANT_RECEPTOR_ ACTIVITY	Not recurrent	26	-0,61	-2,04	0,000	0,014	0,424	2995	tags=38%, list=15%, signal=45%
REACTOME_PLASMA_LIPOPROTEIN_ ASSEMBLY	Not recurrent	19	-0,64	-2,04	0,000	0,015	0,415	3021	tags=53%, list=15%, signal=62%
GOBP_DETECTION_OF_STIMULUS	Not recurrent	322	-0,39	-2,03	0,000	0,015	0,450	2410	tags=26%, list=12%, signal=30%
GOMF_MONOOXYGENASE_ACTIVITY	Not recurrent	100	-0,45	-2,02	0,000	0,017	0,508	2685	tags=31%, list=13%, signal=36%
GOMF_METALLOCARBOXYPEPTIDASE_ACTIVITY	Not recurrent	30	-0,57	-2,02	0,000	0,017	0,508	3947	tags=47%, list=20%, signal=58%
REACTOME_BINDING_AND_UPTAKE_OF_LIGANDS_BY_SCAVENGER_ RECEPTORS	Not recurrent	44	-0,53	-2,01	0,000	0,017	0,539	1549	tags=30%, list=8%, signal=32%
GOMF_MHC_CLASS_I_RECEPTOR_ ACTIVITY	Not recurrent	17	-0,66	-2,00	0,000	0,019	0,577	3029	tags=65%, list=15%, signal=76%
GOMF_ARACHIDONIC_ACID_ EPOXYGENASE_ACTIVITY	Not recurrent	16	-0,68	-1,99	0,002	0,020	0,611	1323	tags=38%, list=7%, signal=40%

GEN SET NAME	ENRICHMENT IN PHENOTYPE	SIZE	ES	NES	NOM p-val	FDR q-val	FWER p-val	RANK AT MAX	LEADING EDGE
REACTOME_COMMON_PATHWAY_OF_ FIBRIN_CLOT_FORMATION	Not recurrent	22	-0,62	-1,97	0,000	0,020	0,680	2385	tags=41%, list=12%, signal=46%
GOMF_BITTER_TASTE_RECEPTOR_ ACTIVITY	Not recurrent	19	-0,63	-1,98	0,000	0,021	0,644	2143	tags=47%, list=11%, signal=53%
GOCC_BLOOD_MICROPARTICLE	Not recurrent	122	-0,43	-1,98	0,000	0,021	0,656	2161	tags=29%, list=11%, signal=32%
GOMF_CARBOXYPEPTIDASE_ACTIVITY	Not recurrent	45	-0,51	-1,98	0,000	0,021	0,661	3947	tags=47%, list=20%, signal=58%
GOCC_COMPLEX_OF_COLLAGEN_ TRIMERS	Not recurrent	21	-0,62	-1,97	0,000	0,021	0,676	910	tags=33%, list=5%, signal=35%
GOBP_AMMONIUM_ION_METABOLIC_ PROCESS	Not recurrent	23	-0,62	-1,97	0,000	0,021	0,697	1294	tags=35%, list=6%, signal=37%
GOBP_COMPLEMENT_ACTIVATION_ ALTERNATIVE_PATHWAY	Not recurrent	16	-0,66	-1,95	0,000	0,025	0,782	2033	tags=56%, list=10%, signal=63%
GOBP_NATURAL_KILLER_CELL_ ACTIVATION_INVOLVED_IN_IMMUNE_ RESPONSE	Not recurrent	32	-0,55	-1,95	0,003	0,025	0,785	1000	tags=28%, list=5%, signal=30%
GOBP_REGULATION_OF_STEROID_ HORMONE_SECRETION	Not recurrent	22	-0,61	-1,94	0,000	0,026	0,806	2864	tags=45%, list=14%, signal=53%
GOBP_KILLING_OF_CELLS_OF_ ANOTHER_ORGANISM	Not recurrent	42	-0,52	-1,94	0,000	0,026	0,809	2240	tags=33%, list=11%, signal=37%
GOBP_PIRNA_METABOLIC_PROCESS	Not recurrent	24	-0,59	-1,94	0,000	0,026	0,816	4139	tags=50%, list=21%, signal=63%
GOMF_PEPTIDE_RECEPTOR_ACTIVITY	Not recurrent	147	-0,4	-1,94	0,000	0,026	0,835	5189	tags=43%, list=26%, signal=57%
GOBP_EPOXYGENASE_P450_PATHWAY	Not recurrent	18	-0,63	-1,94	0,002	0,027	0,833	3619	tags=50%, list=18%, signal=61%

GEN SET NAME	ENRICHMENT IN PHENOTYPE	SIZE	ES	NES	NOM p-val	FDR q-val	FWER p-val	RANK AT MAX	LEADING EDGE
GOMF_IMMUNE_RECEPTOR_A CTIVITY	Not recurrent	133	-0,4	-1,93	0,000	0,028	0,860	3124	tags=33%, list=16%, signal=39%
GOBP_STEROID_HORMONE_ SECRETION	Not recurrent	27	-0,57	-1,92	0,000	0,028	0,868	2864	tags=44%, list=14%, signal=52%
REACTOME_SENSORY_PERCE PTION	Not recurrent	310	-0,36	-1,92	0,000	0,028	0,874	3354	tags=31%, list=17%, signal=36%
REACTOME_CREATION_OF_C4 _AND_C2_ACTIVATORS	Not recurrent	17	-0,63	-1,92	0,003	0,028	0,878	3508	tags=59%, list=17%, signal=71%
GOMF_ANION_SODIUM_SYMPO RTER_ ACTIVITY	Not recurrent	17	-0,64	-1,92	0,000	0,028	0,885	2553	tags=35%, list=13%, signal=40%
REACTOME_DISEASES_ASSOC IATED_ WITH_GLYCOSAMINOGLYCAN_ METABOLISM	Not recurrent	41	-0,51	-1,91	0,000	0,029	0,896	835	tags=22%, list=4%, signal=23%
REACTOME_FORMATION_OF_F IBRIN_ CLOT_CLOTTING_CASCADE	Not recurrent	39	-0,51	-1,91	0,000	0,030	0,912	5004	tags=46%, list=25%, signal=61%
GOCC_PROTEIN_LIPID_COMPL EX	Not recurrent	39	-0,5	-1,90	0,003	0,032	0,929	2521	tags=41%, list=13%, signal=47%
REACTOME_PEPTIDE_LIGAND_ BINDING_RECEPTORS	Not recurrent	192	-0,38	-1,90	0,000	0,033	0,926	2437	tags=26%, list=12%, signal=29%
KEGG_AUTOIMMUNE_THYROID _ DISEASE	Not recurrent	31	-0,53	-1,89	0,000	0,033	0,938	2547	tags=39%, list=13%, signal=44%
GOBP_HUMORAL_IMMUNE_RE SPONSE	Not recurrent	249	-0,36	-1,89	0,000	0,033	0,940	2033	tags=26%, list=10%, signal=28%
GOMF_ANTIGEN_BINDING	Not recurrent	59	-0,46	-1,89	0,000	0,033	0,941	4141	tags=51%, list=21%, signal=64%
GOMF_LONG_CHAIN_FATTY_A CID_COA_LIGASE_ACTIVITY	Not recurrent	15	-0,63	-1,89	0,005	0,033	0,942	570	tags=20%, list=3%, signal=21%

GEN SET NAME	ENRICHMENT IN PHENOTYPE	SIZE	ES	NES	NOM p-val	FDR q-val	FWER p-val	RANK AT MAX	LEADING EDGE
GOBP_HUMORAL_IMMUNE_RESPONSE_MEDIATED_BY_CIRCULATING_ IMMUNOGLOBULIN	Not recurrent	58	-0,46	-1,89	0,000	0,033	0,945	2033	tags=36%, list=10%, signal=40%
GOCC_COLLAGEN_TRIMER	Not recurrent	81	-0,43	-1,87	0,000	0,036	0,976	1614	tags=23%, list=8%, signal=25%
REACTOME_TRAF6_MEDIATED_IRF7_ ACTIVATION	Not recurrent	29	-0,54	-1,87	0,000	0,036	0,978	1460	tags=28%, list=7%, signal=30%
GOBP_POSITIVE_REGULATION_OF_ MONOCYTE_CHEMOTACTIC_PROTEIN_1_PRODUCTION	Not recurrent	15	-0,65	-1,86	0,005	0,036	0,978	1371	tags=27%, list=7%, signal=29%
REACTOME_CHEMOKINE_RECEPTORS_BIND_CHEMOKINES	Not recurrent	58	-0,47	-1,87	0,000	0,037	0,970	2641	tags=33%, list=13%, signal=38%
GOBP_RESPONSE_TO_DSRNA	Not recurrent	54	-0,47	-1,87	0,000	0,037	0,972	892	tags=20%, list=4%, signal=21%
GOBP_NEGATIVE_REGULATION_OF_ CYTOKINE_PRODUCTION_INVOLVED_IN_IMMUNE_RESPONSE	Not recurrent	34	-0,51	-1,87	0,000	0,037	0,975	2808	tags=35%, list=14%, signal=41%
KEGG_GRAFT_VERSUS_HOST_DISEASE	Not recurrent	21	-0,59	-1,87	0,000	0,037	0,976	3924	tags=52%, list=20%, signal=65%
REACTOME_INITIAL_TRIGGERING_OF_COMPLEMENT	Not recurrent	26	-0,54	-1,86	0,000	0,037	0,983	3508	tags=50%, list=17%, signal=61%
REACTOME_CLASS_C_3_ METABOTROPIC_GLUTAMATE_ PHEROMONE_RECEPTORS	Not recurrent	32	-0,51	-1,86	0,005	0,037	0,984	3930	tags=44%, list=20%, signal=54%
GOMF_OPSONIN_BINDING	Not recurrent	20	-0,58	-1,86	0,005	0,037	0,986	3971	tags=55%, list=20%, signal=69%
GOBP_DNA_METHYLATION_INVOLVED_ IN_GAMETE_GENERATION	Not recurrent	20	-0,58	-1,87	0,003	0,038	0,970	3505	tags=40%, list=17%, signal=48%

GEN SET NAME	ENRICHMENT IN PHENOTYPE	SIZE	ES	NES	NOM p-val	FDR q-val	FWER p-val	RANK AT MAX	LEADING EDGE
GOMF_STEROID_HYDROXYLASE_ ACTIVITY	Not recurrent	37	-0,51	-1,87	0,003	0,038	0,975	3715	tags=46%, list=19%, signal=56%
GOBP_TOLERANCE_INDUCTION	Not recurrent	28	-0,54	-1,85	0,000	0,038	0,988	2795	tags=36%, list=14%, signal=41%
REACTOME_CYTOCHROME_P450_ ARRANGED_BY_SUBSTRATE_TYPE	Not recurrent	63	-0,44	-1,85	0,000	0,040	0,990	2648	tags=35%, list=13%, signal=40%
GOBP_POSITIVE_REGULATION_OF_ NATURAL_KILLER_CELL_MEDIATED_ IMMUNITY	Not recurrent	27	-0,54	-1,84	0,000	0,043	0,993	2122	tags=33%, list=11%, signal=37%
GOMF_C_C_CHEMOKINE_BINDING	Not recurrent	24	-0,55	-1,83	0,000	0,044	0,994	2995	tags=42%, list=15%, signal=49%
REACTOME_SYNTHESIS_OF_BILE_ ACIDS_AND_BILE_SALTS_VIA_27_ HYDROXYCHOLESTEROL	Not recurrent	15	-0,62	-1,83	0,000	0,045	0,994	1641	tags=47%, list=8%, signal=51%
GOBP_CELLULAR_DEFENSE_ RESPONSE	Not recurrent	51	-0,45	-1,82	0,000	0,047	0,995	1945	tags=33%, list=10%, signal=37%

Table S11. GSEA analysis between 5-FU, FOLFIRI and FOLFLOX treated recurrent and non-recurrent phenotypes for the 5000 more explicative genes selected by RFE algorithm form merged matrix. Output table of the GSEA analysis between 5-FU, FOLFIRI and FOLFLOX treated recurrent and non-recurrent phenotypes for the 5000 more explicative genes selected by RFE algorithm form merged matrix. To perform the analysis, the perturbation gene sets contained in c2.cgp.v2023.1.Hs.symbols.gmt databases were used. Only enriched gene sets with an FDRq-val< 0.05 are shown.

GEN SET NAME	ENRICHMENT IN PHENOTYPE	SIZE	ES	NES	NOM p-val	FDR q-val	FWER p-val	RANK AT MAX	LEADING EDGE
ANDERSEN_CHOLANGIOCARCI NOMA_ CLASS2	Recurrent	51	0.59	2.32	0.000	0.000	0.001	696	tags=51%, list=15%, signal=59%
ONDER_CDH1_TARGETS_2_DN	Recurrent	121	0.51	2.31	0.000	0.000	0.001	907	tags=46%, list=20%, signal=56%
SABATES_COLORECTAL_ADEN OMA_UP	Recurrent	24	0.69	2.26	0.000	0.000	0.001	414	tags=50%, list=9%, signal=55%
WAMUNYOKOLI_OVARIAN_CAN CER_ LMP_UP	Recurrent	82	0.52	2.25	0.000	0.000	0.001	1757	tags=71%, list=38%, signal=112%
KOINUMA_TARGETS_OF_SMAD 2_OR_ SMAD3	Recurrent	242	0.47	2.36	0.000	0.001	0.001	1670	tags=60%, list=36%, signal=89%
RICKMAN_TUMOR_DIFFERENTI ATED_ WELL_VS_POORLY_DN	Recurrent	98	0.52	2.34	0.000	0.001	0.001	1260	tags=55%, list=27%, signal=74%
CHARAFE_BREAST_CANCER_L UMINAL_VS_MESENCHYMAL_U P	Recurrent	132	0.48	2.21	0.000	0.001	0.003	1038	tags=47%, list=22%, signal=59%
TOOKER_GEMCITABINE_RESIS TANCE_DN	Recurrent	25	0.65	2.16	0.000	0.001	0.011	1218	tags=68%, list=26%, signal=92%
KIM_RESPONSE_TO_TSA_AND _ DECITABINE_UP	Recurrent	48	0.56	2.16	0.000	0.001	0.011	888	tags=48%, list=19%, signal=59%
HOOI_ST7_TARGETS_UP	Recurrent	22	0.68	2.16	0.000	0.001	0.011	445	tags=36%, list=10%, signal=40%
WU_CELL_MIGRATION	Recurrent	53	0.54	2.15	0.000	0.001	0.014	831	tags=45%, list=18%, signal=55%

GEN SET NAME	ENRICHMENT IN PHENOTYPE	SIZE	ES	NES	NOM p-val	FDR q-val	FWER p-val	RANK AT MAX	LEADING EDGE
**JAEGER_METASTASIS_UP**	Recurrent	76	0.51	2.14	0.000	0.001	0.014	907	tags=50%, list=20%, signal=61%
**HUANG_DASATINIB_RESISTANCE_DN**	Recurrent	18	0.71	2.14	0.000	0.001	0.015	945	tags=67%, list=20%, signal=83%
**PHONG_TNF_RESPONSE_VIA_P38_ COMPLETE**	Recurrent	51	0.56	2.19	0.000	0.002	0.010	1140	tags=53%, list=25%, signal=69%
**AMIT_EGF_RESPONSE_480_HELA**	Recurrent	47	0.57	2.18	0.000	0.002	0.010	800	tags=43%, list=17%, signal=51%
**BASAKI_YBX1_TARGETS_DN**	Recurrent	94	0.51	2.18	0.000	0.002	0.011	1119	tags=46%, list=24%, signal=59%
**BILD_HRAS_ONCOGENIC_SIGNATURE**	Recurrent	61	0.52	2.12	0.000	0.002	0.029	1161	tags=54%, list=25%, signal=71%
**HOUNKPE_HOUSEKEEPING_GENES**	Recurrent	281	0.41	2.12	0.000	0.002	0.029	2042	tags=65%, list=44%, signal=110%
**ENK_UV_RESPONSE_EPIDERMIS_UP**	Recurrent	65	0.52	2.12	0.000	0.002	0.029	1016	tags=46%, list=22%, signal=58%
**MASSARWEH_TAMOXIFEN_ RESISTANCE_UP**	Recurrent	147	0.45	2.11	0.000	0.003	0.037	1446	tags=54%, list=31%, signal=76%
**NAGASHIMA_NRG1_SIGNALING_UP**	Recurrent	59	0.52	2.09	0.000	0.003	0.043	1119	tags=51%, list=24%, signal=66%
**PROVENZANI_METASTASIS_DN**	Recurrent	40	0.57	2.07	0.000	0.003	0.052	1005	tags=50%, list=22%, signal=63%
**LEI_MYB_TARGETS**	Recurrent	88	0.47	2.07	0.000	0.003	0.052	1154	tags=45%, list=25%, signal=59%
**ZWANG_CLASS_3_TRANSIENTLY_ INDUCED_BY_EGF**	Recurrent	53	0.52	2.06	0.000	0.004	0.061	1436	tags=60%, list=31%, signal=86%

GEN SET NAME	ENRICHMENT IN PHENOTYPE	SIZE	ES	NES	NOM p-val	FDR q-val	FWER p-val	RANK AT MAX	LEADING EDGE
SCHAEFFER_PROSTATE_ DEVELOPMENT_6HR_DN	Recurrent	113	0.45	2.05	0.000	0.004	0.069	1618	tags=58%, list=35%, signal=87%
RADMACHER_AML_PROGNOSI S	Recurrent	21	0.65	2.05	0.000	0.004	0.075	586	tags=48%, list=13%, signal=54%
NUYTTEN_NIPP1_TARGETS_DN	Recurrent	196	0.42	2.05	0.000	0.004	0.077	1721	tags=56%, list=37%, signal=85%
GENTILE_UV_HIGH_DOSE_DN	Recurrent	77	0.48	2.04	0.000	0.004	0.082	1561	tags=55%, list=34%, signal=81%
MILI_PSEUDOPODIA_HAPTOTA XIS_DN	Recurrent	169	0.42	2.04	0.000	0.004	0.085	1227	tags=42%, list=26%, signal=55%
BOSCO_EPITHELIAL_DIFFEREN TIATION_MODULE	Recurrent	17	0.68	2.03	0.000	0.004	0.089	545	tags=53%, list=12%, signal=60%
GROSS_HYPOXIA_VIA_ELK3_A ND_ HIF1A_UP	Recurrent	41	0.54	2.03	0.000	0.004	0.098	828	tags=46%, list=18%, signal=56%
BLANCO_MELO_BETA_INTERF ERON_ TREATED_BRONCHIAL_EPITHE LIAL_ CELLS_DN	Recurrent	40	0.55	2.03	0.000	0.005	0.098	679	tags=33%, list=15%, signal=38%
RICKMAN_METASTASIS_DN	Recurrent	86	0.47	2.02	0.000	0.005	0.110	1338	tags=49%, list=29%, signal=67%
HUPER_BREAST_BASAL_VS_ LUMINAL_DN	Recurrent	19	0.65	2.01	0.002	0.005	0.118	1403	tags=79%, list=30%, signal=113%
LIM_MAMMARY_STEM_CELL_D N	Recurrent	103	0.46	2.01	0.000	0.005	0.119	1599	tags=60%, list=34%, signal=90%
CREIGHTON_ENDOCRINE_THE RAPY_ RESISTANCE_3	Recurrent	193	0.41	2.01	0.000	0.005	0.121	1218	tags=43%, list=26%, signal=56%
RICKMAN_HEAD_AND_NECK_ CANCER_C	Recurrent	25	0.61	2.01	0.000	0.005	0.121	147	tags=32%, list=3%, signal=33%

GEN SET NAME	ENRICHMENT IN PHENOTYPE	SIZE	ES	NES	NOM p-val	FDR q-val	FWER p-val	RANK AT MAX	LEADING EDGE
**MONNIER_POSTRADIATION_TUMOR_ ESCAPE_UP**	Recurrent	107	0.44	2	0.000	0.005	0.121	1463	tags=48%, list=32%, signal=68%
**DURCHDEWALD_SKIN_ CARCINOGENESIS_DN**	Recurrent	55	0.51	2	0.000	0.005	0.125	835	tags=38%, list=18%, signal=46%
**GRAESSMANN_APOPTOSIS_BY_ DOXORUBICIN_UP**	Recurrent	262	0.39	1.98	0.000	0.006	0.167	1421	tags=46%, list=31%, signal=63%
**DAZARD_RESPONSE_TO_UV_NHEK_DN**	Recurrent	64	0.48	1.97	0.000	0.007	0.194	1620	tags=56%, list=35%, signal=85%
**PHONG_TNF_RESPONSE_VIA_P38_ PARTIAL**	Recurrent	37	0.54	1.97	0.000	0.007	0.198	438	tags=35%, list=9%, signal=38%
**MARTORIATI_MDM4_TARGETS_ FETAL_LIVER_UP**	Recurrent	55	0.49	1.96	0.000	0.008	0.216	1111	tags=40%, list=24%, signal=52%
**WAMUNYOKOLI_OVARIAN_CANCER_ GRADES_1_2_UP**	Recurrent	47	0.52	1.96	0.002	0.008	0.222	1094	tags=47%, list=24%, signal=61%
**ELVIDGE_HYPOXIA_BY_DMOG_UP**	Recurrent	31	0.57	1.96	0.000	0.008	0.229	749	tags=45%, list=16%, signal=53%
**ENK_UV_RESPONSE_KERATINOCYTE_ UP**	Recurrent	135	0.42	1.96	0.000	0.008	0.235	1464	tags=47%, list=32%, signal=67%
**PEDERSEN_METASTASIS_BY_ERBB2_ ISOFORM_7**	Recurrent	101	0.44	1.95	0.000	0.008	0.244	1140	tags=49%, list=25%, signal=63%
**GARY_CD5_TARGETS_UP**	Recurrent	116	0.44	1.95	0.001	0.008	0.259	1618	tags=54%, list=35%, signal=81%
**CREIGHTON_ENDOCRINE_THERAPY_ RESISTANCE_5**	Recurrent	130	0.42	1.93	0.000	0.011	0.330	1272	tags=45%, list=27%, signal=60%
**COLDREN_GEFITINIB_RESISTANCE_DN**	Recurrent	78	0.46	1.93	0.000	0.011	0.340	945	tags=45%, list=20%, signal=55%

GEN SET NAME	ENRICHMENT IN PHENOTYPE	SIZE	ES	NES	NOM p-val	FDR q-val	FWER p-val	RANK AT MAX	LEADING EDGE
FORTSCHEGGER_PHF8_TARGETS_DN	Recurrent	192	0.39	1.93	0.000	0.011	0.340	1630	tags=53%, list=35%, signal=78%
LIN_SILENCED_BY_TUMOR_ MICROENVIRONMENT	Recurrent	32	0.56	1.92	0.000	0.011	0.340	818	tags=50%, list=18%, signal=60%
LIAO_METASTASIS	Recurrent	122	0.42	1.92	0.000	0.011	0.361	1226	tags=46%, list=26%, signal=61%
WINTER_HYPOXIA_UP	Recurrent	25	0.58	1.92	0.000	0.011	0.364	1309	tags=64%, list=28%, signal=89%
PEDERSEN_TARGETS_OF_611CTF_ ISOFORM_OF_ERBB2	Recurrent	23	0.6	1.92	0.000	0.011	0.367	739	tags=52%, list=16%, signal=62%
CHARAFE_BREAST_CANCER_BASAL_ VS_MESENCHYMAL_UP	Recurrent	33	0.53	1.91	0.000	0.011	0.373	1110	tags=61%, list=24%, signal=79%
JINESH_BLEBBISHIELD_TRANSFORMED_STEM_CELL_SPHERES_UP	Recurrent	49	0.5	1.91	0.000	0.011	0.373	991	tags=49%, list=21%, signal=62%
SMITH_TERT_TARGETS_UP	Recurrent	39	0.51	1.91	0.000	0.011	0.398	1379	tags=56%, list=30%, signal=80%
KRIGE_RESPONSE_TO_TOSEDOSTAT_ 24HR_UP	Recurrent	171	0.4	1.91	0.000	0.012	0.398	1497	tags=53%, list=32%, signal=76%
JINESH_BLEBBISHIELD_TO_IMMUNE_ CELL_FUSION_PBSHMS_UP	Recurrent	94	0.43	1.9	0.000	0.012	0.415	1386	tags=45%, list=30%, signal=62%
BENPORATH_NANOG_TARGETS	Recurrent	243	0.38	1.9	0.000	0.012	0.419	1714	tags=55%, list=37%, signal=82%
LINDGREN_BLADDER_CANCER_ CLUSTER_3_DN	Recurrent	65	0.46	1.9	0.000	0.012	0.429	1620	tags=58%, list=35%, signal=89%
BERENJENO_TRANSFORMED_BY_ RHOA_UP	Recurrent	120	0.42	1.9	0.000	0.012	0.434	1571	tags=49%, list=34%, signal=72%

GEN SET NAME	ENRICHMENT IN PHENOTYPE	SIZE	ES	NES	NOM p-val	FDR q-val	FWER p-val	RANK AT MAX	LEADING EDGE
BENPORATH_SOX2_TARGETS	Recurrent	183	0.39	1.89	0.000	0.013	0.459	1798	tags=58%, list=39%, signal=92%
SPIELMAN_LYMPHOBLAST_EUROPEAN_VS_ASIAN_UP	Recurrent	123	0.41	1.89	0.000	0.013	0.459	2050	tags=63%, list=44%, signal=111%
BASAKI_YBX1_TARGETS_UP	Recurrent	78	0.45	1.89	0.000	0.013	0.463	1192	tags=41%, list=26%, signal=54%
ZHANG_RESPONSE_TO_IKK_INHIBITOR_AND_TNF_UP	Recurrent	59	0.48	1.89	0.000	0.013	0.477	1011	tags=42%, list=22%, signal=53%
CHARAFE_BREAST_CANCER_LUMINAL_VS_BASAL_DN	Recurrent	105	0.42	1.89	0.000	0.013	0.477	925	tags=37%, list=20%, signal=45%
RODRIGUES_NTN1_TARGETS_DN	Recurrent	45	0.5	1.88	0.000	0.013	0.482	1771	tags=71%, list=38%, signal=114%
GRAESSMANN_APOPTOSIS_BY_ _ DOXORUBICIN_DN	Recurrent	395	0.35	1.88	0.000	0.013	0.493	1925	tags=55%, list=41%, signal=87%
GRAESSMANN_RESPONSE_TO _MC_ AND_DOXORUBICIN_UP	Recurrent	136	0.41	1.88	0.000	0.013	0.495	1500	tags=46%, list=32%, signal=66%
MENSE_HYPOXIA_UP	Recurrent	22	0.59	1.88	0.000	0.013	0.506	777	tags=45%, list=17%, signal=54%
GINESTIER_BREAST_CANCER_ 20Q13_ AMPLIFICATION_DN	Recurrent	40	0.51	1.87	0.000	0.014	0.529	2090	tags=80%, list=45%, signal=144%
SHEDDEN_LUNG_CANCER_GOOD_ SURVIVAL_A5	Recurrent	21	0.59	1.87	0.000	0.015	0.550	1462	tags=67%, list=31%, signal=97%
RHEIN_ALL_GLUCOCORTICOID _ THERAPY_UP	Recurrent	16	0.64	1.87	0.002	0.015	0.552	1497	tags=88%, list=32%, signal=129%
LANDIS_ERBB2_BREAST_TUMORS_ 324_UP	Recurrent	44	0.49	1.86	0.000	0.015	0.570	1596	tags=57%, list=34%, signal=86%

GEN SET NAME	ENRICHMENT IN PHENOTYPE	SIZE	ES	NES	NOM p-val	FDR q-val	FWER p-val	RANK AT MAX	LEADING EDGE
SCHAEFFER_PROSTATE_DEVELOPMENT_48HR_UP	Recurrent	117	0.41	1.86	0.002	0.015	0.577	1102	tags=41%, list=24%, signal=52%
DODD_NASOPHARYNGEAL_CARCINOMA_UP	Recurrent	450	0.34	1.86	0.000	0.015	0.581	1217	tags=38%, list=26%, signal=47%
GLASS_IGF2BP1_CLIP_TARGETS_KNOCKDOWN_DN	Recurrent	37	0.52	1.86	0.000	0.016	0.593	1285	tags=49%, list=28%, signal=67%
GHANDHI_DIRECT_IRRADIATION_UP	Recurrent	24	0.57	1.85	0.005	0.016	0.624	723	tags=38%, list=16%, signal=44%
SCHUETZ_BREAST_CANCER_DUCTAL_ INVASIVE_DN	Recurrent	28	0.54	1.85	0.002	0.017	0.619	707	tags=36%, list=15%, signal=42%
MITSIADES_RESPONSE_TO_APLIDIN_ UP	Recurrent	114	0.41	1.85	0.000	0.017	0.631	1684	tags=51%, list=36%, signal=78%
ELVIDGE_HIF1A_TARGETS_DN	Recurrent	18	0.6	1.85	0.002	0.017	0.642	907	tags=50%, list=20%, signal=62%
ELVIDGE_HYPOXIA_UP	Recurrent	39	0.5	1.84	0.002	0.018	0.661	907	tags=44%, list=20%, signal=54%
BROWNE_HCMV_INFECTION_6HR_DN	Recurrent	28	0.53	1.84	0.005	0.018	0.662	439	tags=29%, list=9%, signal=31%
RASHI_RESPONSE_TO_IONIZING_ RADIATION_2	Recurrent	28	0.54	1.84	0.000	0.018	0.674	1272	tags=61%, list=27%, signal=83%
HOELZEL_NF1_TARGETS_UP	Recurrent	28	0.55	1.84	0.002	0.018	0.682	302	tags=39%, list=7%, signal=42%
EPPERT_PROGENITOR	Recurrent	30	0.53	1.84	0.002	0.018	0.684	1124	tags=43%, list=24%, signal=57%
KIM_WT1_TARGETS_UP	Recurrent	59	0.46	1.84	0.000	0.018	0.690	1446	tags=56%, list=31%, signal=80%

GEN SET NAME	ENRICHMENT IN PHENOTYPE	SIZE	ES	NES	NOM p-val	FDR q-val	FWER p-val	RANK AT MAX	LEADING EDGE
BLANCO_MELO_COVID19_SARS_COV_2_INFECTION_A594_CELLS_UP	Recurrent	17	0.61	1.84	0.002	0.018	0.690	445	tags=41%, list=10%, signal=45%
HUANG_GATA2_TARGETS_DN	Recurrent	15	0.63	1.83	0.003	0.019	0.710	483	tags=47%, list=10%, signal=52%
LASTOWSKA_NEUROBLASTOMA_COPY_NUMBER_DN	Recurrent	201	0.38	1.83	0.000	0.019	0.720	1981	tags=58%, list=43%, signal=96%
OISHI_CHOLANGIOMA_STEM_CELL_ LIKE_DN	Recurrent	76	0.43	1.83	0.000	0.019	0.725	634	tags=36%, list=14%, signal=40%
FISCHER_G2_M_CELL_CYCLE	Recurrent	51	0.47	1.83	0.000	0.019	0.731	1285	tags=41%, list=28%, signal=56%
PICCALUGA_ANGIOIMMUNOBLASTIC_ LYMPHOMA_DN	Recurrent	28	0.54	1.82	0.007	0.020	0.744	1769	tags=71%, list=38%, signal=115%
DORN_ADENOVIRUS_INFECTION_24HR_DN	Recurrent	17	0.62	1.82	0.003	0.020	0.749	1555	tags=82%, list=33%, signal=123%
LINDGREN_BLADDER_CANCER_ _ CLUSTER_2A_DN	Recurrent	34	0.51	1.82	0.000	0.020	0.755	1329	tags=53%, list=29%, signal=74%
KINSEY_TARGETS_OF_EWSR1_FLII_ FUSION_UP	Recurrent	263	0.36	1.82	0.000	0.020	0.757	1240	tags=37%, list=27%, signal=48%
BUYTAERT_PHOTODYNAMIC_THERAPY_STRESS_DN	Recurrent	161	0.38	1.81	0.000	0.020	0.779	1692	tags=49%, list=36%, signal=75%
NAGASHIMA_EGF_SIGNALING_UP	Recurrent	18	0.6	1.81	0.002	0.020	0.779	1436	tags=72%, list=31%, signal=104%
DELYS_THYROID_CANCER_UP	Recurrent	118	0.4	1.81	0.000	0.021	0.770	1054	tags=42%, list=23%, signal=52%
GAUSSMANN_MLL_AF4_FUSION_ TARGETS_A_DN	Recurrent	21	0.57	1.81	0.003	0.021	0.777	445	tags=38%, list=10%, signal=42%

GEN SET NAME	ENRICHMENT IN PHENOTYPE	SIZE	ES	NES	NOM p-val	FDR q-val	FWER p-val	RANK AT MAX	LEADING EDGE
**KIM_WT1_TARGETS_12HR_DN**	Recurrent	43	0.47	1.81	0.002	0.021	0.777	699	tags=35%, list=15%, signal=41%
**MARSON_BOUND_BY_FOXP3_ UNSTIMULATED**	Recurrent	302	0.35	1.81	0.000	0.021	0.793	1563	tags=45%, list=34%, signal=63%
**ZHONG_RESPONSE_TO_AZACI TIDINE_ AND_TSA_UP**	Recurrent	44	0.48	1.81	0.003	0.021	0.801	1344	tags=55%, list=29%, signal=76%
**KRIGE_RESPONSE_TO_TOSED OSTAT_6HR_UP**	Recurrent	214	0.36	1.81	0.000	0.021	0.804	1497	tags=49%, list=32%, signal=69%
**DAVICIONI_MOLECULAR_ARMS _VS_ ERMS_UP**	Recurrent	73	0.42	1.8	0.000	0.021	0.821	1036	tags=44%, list=22%, signal=56%
**DIAZ_CHRONIC_MYELOGENOU S_ LEUKEMIA_UP**	Recurrent	294	0.35	1.8	0.000	0.021	0.825	1928	tags=58%, list=42%, signal=93%
**UDAYAKUMAR_MED1_TARGET S_DN**	Recurrent	65	0.44	1.8	0.002	0.021	0.825	1709	tags=63%, list=37%, signal=98%
**LINSLEY_MIR16_TARGETS**	Recurrent	53	0.46	1.8	0.003	0.021	0.825	1849	tags=62%, list=40%, signal=102%
**DEBIASI_APOPTOSIS_BY_REO VIRUS_ INFECTION_DN**	Recurrent	63	0.44	1.8	0.002	0.022	0.813	1231	tags=44%, list=27%, signal=60%
**HOLLERN_EMT_BREAST_TUMO R_DN**	Recurrent	36	0.51	1.8	0.002	0.022	0.814	1708	tags=72%, list=37%, signal=113%
**KOBAYASHI_EGFR_SIGNALING _24HR_DN**	Recurrent	49	0.46	1.8	0.002	0.022	0.816	1061	tags=37%, list=23%, signal=47%
**WANG_BARRETTS_ESOPHAGU S_UP**	Recurrent	15	0.62	1.8	0.007	0.022	0.821	1090	tags=60%, list=23%, signal=78%
**FEVR_CTNNB1_TARGETS_UP**	Recurrent	160	0.38	1.79	0.000	0.022	0.837	1426	tags=45%, list=31%, signal=63%

GEN SET NAME	ENRICHMENT IN PHENOTYPE	SIZE	ES	NES	NOM p-val	FDR q-val	FWER p-val	RANK AT MAX	LEADING EDGE
DACOSTA_UV_RESPONSE_VIA _ERCC3_UP	Recurrent	69	0.43	1.79	0.002	0.022	0.841	1235	tags=46%, list=27%, signal=62%
RICKMAN_TUMOR_DIFFERENTI ATED_ WELL_VS_MODERATELY_DN	Recurrent	30	0.52	1.79	0.007	0.022	0.845	948	tags=43%, list=20%, signal=54%
FOURNIER_ACINAR_DEVELOP MENT_ LATE_2	Recurrent	64	0.43	1.79	0.002	0.022	0.852	1609	tags=47%, list=35%, signal=71%
HIRSCH_CELLULAR_TRANSFO RMATION_SIGNATURE_UP	Recurrent	57	0.45	1.79	0.000	0.023	0.858	1492	tags=56%, list=32%, signal=82%
SHETH_LIVER_CANCER_VS_TX NIP_ LOSS_PAM1	Recurrent	62	0.44	1.78	0.002	0.023	0.871	1004	tags=40%, list=22%, signal=51%
OUELLET_CULTURED_OVARIA N_ CANCER_INVASIVE_VS_LMP_U P	Recurrent	18	0.6	1.78	0.005	0.024	0.875	1042	tags=56%, list=22%, signal=71%
SENGUPTA_NASOPHARYNGEA L_ CARCINOMA_DN	Recurrent	98	0.4	1.78	0.000	0.024	0.879	774	tags=36%, list=17%, signal=42%
WEST_ADRENOCORTICAL_TUM OR_UP	Recurrent	67	0.43	1.78	0.002	0.025	0.885	1360	tags=42%, list=29%, signal=58%
ZWANG_EGF_INTERVAL_DN	Recurrent	64	0.44	1.77	0.000	0.026	0.902	1868	tags=63%, list=40%, signal=103%
SENESE_HDAC1_TARGETS_UP	Recurrent	96	0.4	1.77	0.000	0.026	0.905	992	tags=35%, list=21%, signal=44%
GRYDER_PAX3FOXO1_ENHANC ERS_KO_DOWN	Recurrent	97	0.4	1.77	0.002	0.026	0.907	1231	tags=42%, list=27%, signal=56%
NUYTTEN_EZH2_TARGETS_DN	Recurrent	212	0.36	1.77	0.000	0.026	0.912	1685	tags=52%, list=36%, signal=78%
CHIARADONNA_NEOPLASTIC_ TRANSFORMATION_KRAS_DN	Recurrent	35	0.49	1.76	0.002	0.027	0.923	1060	tags=49%, list=23%, signal=62%

GEN SET NAME	ENRICHMENT IN PHENOTYPE	SIZE	ES	NES	NOM p-val	FDR q-val	FWER p-val	RANK AT MAX	LEADING EDGE
BLUM_RESPONSE_TO_SALIRASIB_UP	Recurrent	55	0.45	1.76	0.000	0.028	0.930	1622	tags=55%, list=35%, signal=83%
PHONG_TNF_TARGETS_UP	Recurrent	17	0.59	1.76	0.009	0.028	0.930	1532	tags=76%, list=33%, signal=114%
ZWANG_EGF_PERSISTENTLY_DN	Recurrent	17	0.59	1.76	0.005	0.028	0.934	1567	tags=59%, list=34%, signal=88%
GRYDER_PAX3FOXO1_ENHANCERS_IN_TADS	Recurrent	218	0.35	1.75	0.000	0.030	0.951	1499	tags=44%, list=32%, signal=62%
SAGIV_CD24_TARGETS_DN	Recurrent	15	0.6	1.75	0.007	0.030	0.954	818	tags=60%, list=18%, signal=73%
MARTORIATI_MDM4_TARGETS_FETAL_LIVER_DN	Recurrent	104	0.39	1.75	0.001	0.030	0.955	1289	tags=42%, list=28%, signal=57%
MCBRYAN_PUBERTAL_BREAST_4_ 5WK_UP	Recurrent	64	0.42	1.75	0.003	0.030	0.955	1253	tags=47%, list=27%, signal=63%
CHYLA_CBFA2T3_TARGETS_DN	Recurrent	73	0.41	1.75	0.000	0.030	0.960	1532	tags=52%, list=33%, signal=76%
ROSS_AML_WITH_AML1_ETO_FUSION	Recurrent	19	0.57	1.75	0.003	0.030	0.960	532	tags=37%, list=11%, signal=41%
MARSON_BOUND_BY_E2F4_UNSTIMULATED	Recurrent	142	0.37	1.75	0.000	0.030	0.961	1480	tags=44%, list=32%, signal=62%
BROWNE_HCMV_INFECTION_14HR_DN	Recurrent	76	0.42	1.75	0.000	0.031	0.961	1824	tags=63%, list=39%, signal=102%
CHICAS_RB1_TARGETS_SENESCENT	Recurrent	130	0.37	1.74	0.000	0.032	0.966	1125	tags=36%, list=24%, signal=46%
KYNG_DNA_DAMAGE_DN	Recurrent	45	0.45	1.74	0.002	0.033	0.966	1251	tags=49%, list=27%, signal=66%

GEN SET NAME	ENRICHMENT IN PHENOTYPE	SIZE	ES	NES	NOM p-val	FDR q-val	FWER p-val	RANK AT MAX	LEADING EDGE
HUANG_DASATINIB_SENSITIVITY_UP	Recurrent	21	0.54	1.74	0.007	0.033	0.966	1630	tags=71%, list=35%, signal=110%
STEIN_ESRRA_TARGETS	Recurrent	130	0.37	1.73	0.000	0.035	0.976	1722	tags=57%, list=37%, signal=88%
SWEET_KRAS_TARGETS_DN	Recurrent	15	0.61	1.72	0.004	0.036	0.980	889	tags=60%, list=19%, signal=74%
STEIN_ESRRA_TARGETS_UP	Recurrent	86	0.4	1.72	0.003	0.036	0.980	1722	tags=58%, list=37%, signal=91%
FERREIRA_EWINGS_SARCOMA_ UNSTABLE_VS_STABLE_DN	Recurrent	28	0.51	1.72	0.005	0.036	0.980	1200	tags=54%, list=26%, signal=72%
RODRIGUES_THYROID_CARCINOMA_ POORLY_DIFFERENTIATED_DN	Recurrent	194	0.35	1.72	0.000	0.036	0.981	1565	tags=47%, list=34%, signal=68%
RICKMAN_HEAD_AND_NECK_ CANCER_A	Recurrent	24	0.52	1.72	0.003	0.037	0.985	346	tags=29%, list=7%, signal=31%
TURASHVILI_BREAST_DUCTAL_ CARCINOMA_VS_DUCTAL_NORMAL_DN	Recurrent	46	0.45	1.71	0.002	0.040	0.988	707	tags=43%, list=15%, signal=51%
YAO_TEMPORAL_RESPONSE_TO_ PROGESTERONE_CLUSTER_14	Recurrent	29	0.5	1.71	0.010	0.040	0.990	1895	tags=69%, list=41%, signal=116%
KIM_ALL_DISORDERS_CALB1_CORR_ UP	Recurrent	131	0.37	1.71	0.000	0.040	0.990	1722	tags=57%, list=37%, signal=88%
SESTO_RESPONSE_TO_UV_C2	Recurrent	17	0.57	1.71	0.012	0.040	0.990	1722	tags=71%, list=37%, signal=112%
SESTO_RESPONSE_TO_UV_C8	Recurrent	15	0.58	1.71	0.009	0.041	0.991	1658	tags=80%, list=36%, signal=124%
DACOSTA_UV_RESPONSE_VIA_ERCC3_DN	Recurrent	176	0.35	1.7	0.000	0.041	0.993	1844	tags=53%, list=40%, signal=85%

GEN SET NAME	ENRICHMENT IN PHENOTYPE	SIZE	ES	NES	NOM p-val	FDR q-val	FWER p-val	RANK AT MAX	LEADING EDGE
HELLER_HDAC_TARGETS_SILENCED_ BY_METHYLATION_UP	Recurrent	107	0.39	1.7	0.000	0.041	0.993	865	tags=36%, list=19%, signal=43%
GOBERT_OLIGODENDROCYTE_ DIFFERENTIATION_DN	Recurrent	239	0.34	1.7	0.001	0.043	0.995	1683	tags=48%, list=36%, signal=71%
HUANG_GATA2_TARGETS_UP	Recurrent	41	0.46	1.7	0.005	0.044	0.997	1386	tags=54%, list=30%, signal=76%
MULLIGHAN_NPM1_SIGNATURE_3_UP	Recurrent	62	0.42	1.69	0.003	0.044	0.997	1373	tags=45%, list=30%, signal=63%
ENK_UV_RESPONSE_KERATINOCYTE_ DN	Recurrent	107	0.38	1.69	0.005	0.045	0.997	1847	tags=54%, list=40%, signal=88%
JOHNSTONE_PARVB_TARGETS_3_UP	Recurrent	109	0.38	1.69	0.001	0.045	0.997	1916	tags=63%, list=41%, signal=105%
ZWANG_CLASS_1_TRANSIENTLY_ INDUCED_BY_EGF	Recurrent	123	0.37	1.69	0.000	0.045	0.997	1356	tags=43%, list=29%, signal=59%
HAMAI_APOPTOSIS_VIA_TRAIL_DN	Recurrent	45	0.44	1.69	0.005	0.046	0.997	800	tags=33%, list=17%, signal=40%
DUTERTRE_ESTRADIOL_RESPONSE_24HR_DN	Recurrent	113	0.37	1.68	0.001	0.047	0.997	1660	tags=50%, list=36%, signal=75%
COULOUARN_TEMPORAL_TGFB1_ SIGNATURE_UP	Recurrent	28	0.49	1.68	0.008	0.047	0.997	1253	tags=50%, list=27%, signal=68%
TIEN_INTESTINE_PROBIOTICS_ 2HR_DN	Recurrent	21	0.53	1.68	0.017	0.047	0.997	1807	tags=76%, list=39%, signal=124%
HAN_SATB1_TARGETS_DN	Recurrent	111	0.37	1.68	0.005	0.047	0.997	763	tags=31%, list=16%, signal=36%
KATSANOU_ELAVL1_TARGETS_DN	Recurrent	30	0.49	1.68	0.006	0.047	0.997	596	tags=30%, list=13%, signal=34%

GEN SET NAME	ENRICHMENT IN PHENOTYPE	SIZE	ES	NES	NOM p-val	FDR q-val	FWER p-val	RANK AT MAX	LEADING EDGE
SASSON_RESPONSE_TO_FORSKOLIN_UP	Recurrent	22	0.53	1.68	0.015	0.047	0.997	1009	tags=50%, list=22%, signal=64%
SHAFFER_IRF4_TARGETS_IN_ACTIVATED_DENDRITIC_CELL	Recurrent	15	0.59	1.68	0.008	0.047	0.997	818	tags=40%, list=18%, signal=48%
ONDER_CDH1_TARGETS_3_DN	Recurrent	15	0.6	1.68	0.011	0.048	0.997	971	tags=67%, list=21%, signal=84%
SENESE_HDAC3_TARGETS_UP	Recurrent	113	0.37	1.68	0.000	0.048	0.998	957	tags=33%, list=21%, signal=40%
FARMER_BREAST_CANCER_BASAL_VS_LULMINAL	Recurrent	75	0.4	1.68	0.007	0.048	0.998	1103	tags=40%, list=24%, signal=52%
GOZGIT_ESR1_TARGETS_UP	Recurrent	40	0.45	1.67	0.011	0.049	0.998	763	tags=38%, list=16%, signal=44%
SOGA_COLORECTAL_CANCER_MYC_ DN	Recurrent	23	0.52	1.67	0.008	0.050	0.999	1182	tags=52%, list=25%, signal=70%
AMIT_EGF_RESPONSE_120_HELA	Recurrent	23	0.51	1.67	0.017	0.050	0.999	1419	tags=65%, list=31%, signal=93%
PEREZ_TP53_TARGETS	Recurrent	307	0.33	1.67	0.000	0.050	0.999	1202	tags=34%, list=26%, signal=43%
PASQUALUCCI_LYMPHOMA_BY_GC_ STAGE_UP	Recurrent	73	0.4	1.66	0.002	0.050	0.999	1274	tags=41%, list=27%, signal=56%
LEE_BMP2_TARGETS_UP	Recurrent	211	0.34	1.66	0.000	0.050	0.999	1469	tags=47%, list=32%, signal=66%
ELVIDGE_HIF1A_AND_HIF2A_TARGETS_DN	Recurrent	21	0.54	1.66	0.012	0.050	0.999	907	tags=48%, list=20%, signal=59%
TIEN_INTESTINE_PROBIOTICS_6HR_DN	Recurrent	45	0.45	1.66	0.005	0.050	0.999	1805	tags=62%, list=39%, signal=101%

GEN SET NAME	ENRICHMENT IN PHENOTYPE	SIZE	ES	NES	NOM p-val	FDR q-val	FWER p-val	RANK AT MAX	LEADING EDGE
SMID_BREAST_CANCER_BASAL_UP	Recurrent	144	0.36	1.66	0.000	0.050	0.999	924	tags=35%, list=20%, signal=43%
RODRIGUES_THYROID_CARCINOMA_ ANAPLASTIC_DN	Recurrent	131	0.36	1.67	0.001	0.051	0.999	977	tags=31%, list=21%, signal=39%
GOBERT_OLIGODENDROCYTE_ DIFFERENTIATION_UP	Recurrent	124	0.36	1.67	0.001	0.051	0.999	1563	tags=44%, list=34%, signal=64%
SENESE_HDAC1_AND_HDAC2_ TARGETS_UP	Recurrent	54	0.42	1.67	0.005	0.051	0.999	786	tags=31%, list=17%, signal=37%
LU_EZH2_TARGETS_UP	Recurrent	83	0.39	1.67	0.003	0.051	0.999	1622	tags=57%, list=35%, signal=85%
GROSS_HYPOXIA_VIA_ELK3_DN	Recurrent	40	0.45	1.66	0.012	0.051	0.999	828	tags=38%, list=18%, signal=45%
TAKAO_RESPONSE_TO_UVB_ RADIATION_DN	Recurrent	15	0.59	1.66	0.006	0.051	0.999	989	tags=60%, list=21%, signal=76%
VECCHI_GASTRIC_CANCER_ADVANCED_VS_EARLY_UP	Non recurrent	35	-0.61	-2.39	0.000	0.001	0.002	632	tags=46%, list=14%, signal=53%
CARRILLOREIXACH_MRS3_VS_ LOWER_RISK_HEPATOBLASTOMA_DN	Non recurrent	34	-0.6	-2.3	0.000	0.001	0.006	659	tags=44%, list=14%, signal=51%
LEE_LIVER_CANCER_SURVIVAL_UP	Non recurrent	42	-0.51	-2.04	0.000	0.016	0.190	591	tags=40%, list=13%, signal=46%
CHIANG_LIVER_CANCER_SUBCLASS_ PROLIFERATION_DN	Non recurrent	42	-0.5	-2.04	0.000	0.020	0.189	591	tags=40%, list=13%, signal=46%
LIU_OVARIAN_CANCER_TUMORS_AND_XENOGRAFTS_XDGS_ DN	Non recurrent	340	-0.36	-2.05	0.000	0.023	0.165	766	tags=30%, list=16%, signal=33%
HSIAO_LIVER_SPECIFIC_GENES	Non recurrent	48	-0.46	-1.88	0.000	0.045	0.595	1012	tags=52%, list=22%, signal=66%

GEN SET NAME	ENRICHMENT IN PHENOTYPE	SIZE	ES	NES	NOM p-val	FDR q-val	FWER p-val	RANK AT MAX	LEADING EDGE
**XU_GH1_EXOGENOUS_TARGETS_DN**	Non recurrent	18	-0.6	-1.9	0.002	0.047	0.500	376	tags=39%, list=8%, signal=42%
**NIKOLSKY_BREAST_CANCER_20Q12_Q13_AMPLICON**	Non recurrent	41	-0.46	-1.88	0.003	0.049	0.575	1026	tags=44%, list=22%, signal=56%

Table S12. Selected DEGs to perform CLUE Query (v.1.1) analysis.

**UPREGULATED**	ABI3BP, ADAMDEC1, ADAMTS1, AKAP13, AKR1B10, ANKRD28, ANXA1, APOBEC1, ARHGAP5, ASPN, ATF3, ATRX, BAMBI, BCAS1, C7, CALD1, CAMK2N1, CCL2, CD177, CD44, CD55, CDC42BPA, CHRDL1, CLCA1, CLDN8, CLINT1, CNN1, COL14A1, COX7A1, CTNNB1, CTSO, CXCL14, CYCS, DAAM1, DDX17, DDX6, DEFA6, DES, DUSP1, EFNB2, EGFR, EGR1, ENPP2, FAM129A, FAM13A, FAM208B, FAM3C, FERMT2, FOS, FOSB, GABRE, GALNT1, GEM, GLIPR1, GOLGA4, HLA-DQA1, HLA-DRB1, HOXB6, IGF1, IGLC1, JUP, KRAS, LMOD1, LTBP1, MAB21L2, MBNL2, MECOM, MGA, MUC1, MUC12, MUC17, MUC2, MUC4, MYH11, MYL9, MYLK, MYO6, MYOCD, NEXN, NFAT5, NFATC2IP, NOTCH2NL, NRP1, OGT, OLFM4, PARP8, PCSK5, PDLIM3, PELI1, PIK3C2A, PLA2G10, PLA2G2A, PLAC8, PLN, PNISR, PNN, PNOC, POSTN, PPA2, PPP1R12A, PPP2R5C, PRAC1, RAB18, RASEF, RBM25, RBM5, REG1A, RHOBTB3, RHOQ, RIF1, RPS27, SCAF11, SCAF4, SECISBP2L, SEPT6, SEPT7, SERPINE1, SERPING1, SFN, SLC26A2, SLC26A3, SLC28A2, SMCHD1, SPAG9, SPARCL1, SPEN, SRGAP1, SRRM2, SULT1B1, SYNM, TAGLN, TBL1XR1, TGFBR1, TMC5, TNC, TNIK, TNS1, TOP1, TOP2A, TOPB1, UGT2B17, USP9X, VPS13B, WASF2, WLS, ZBTB20, ZNF117, ZNF37BP, ZNF532, ZNF595
**DOWNREGULATED**	ANKRD30B, CES1, CFHR4, CRACR2A, CXCL11, CXCL8, GAGE12J, GAGE2C, GAGE1, GAGE4, GAGE8, GLUD2, HLA-DQB1, HSD3B2, IGHA1, IGHD, IGHM, IGHV1-69, IGHV4-31, IGK, IGKC, IGLC1, IL1A, IL1B, KCNJ12, LGR5, LRRC37A3, MRLN, MSTO1, POU5F1P4, PSMB2, PTPRD, RGS16, RXRA, SP8, TCFL5, WDCP, YME1L1

Table S13. CLUE Query (v.1.1) analysis for selected up-regulated and downregulated genes. Output table of the CLUE Query (v.1.1) analysis for selected 150 up-regulated genes an all downregulated. The table shows the compounds with a connectivity score less than (-50).

COMPOUND ID	COMPOUND NAME	DESCRIPTION	CONECTIVITY SCORE
BRD-K94832621	Y-134	Estrogen receptor antagonist	-99.87
BRD-K08547377	irinotecan	Topoisomerase inhibitor	-99.85
BRD-A63546914	RO-04-5595	Glutamate receptor antagonist	-99.78
BRD-K12260308	xanthoxyline	Antifungal	-99.65
BRD-K82164249	andarine	Androgen receptor modulator	-99.54
BRD-K26548821	quinpirole	Dopamine receptor agonist	-99.53
BRD-K54256913	MK-1775	WEE1 kinase inhibitor	-99.5
BRD-A67862938	naftidrofuryl	Adrenergic receptor antagonist	-99.43
BRD-K02283807	GR-32191	Thromboxane receptor antagonist	-99.4
BRD-A36630025	SN-38	Topoisomerase inhibitor	-99.39
BRD-A48237631	mitomycin-c	DNA alkylating agent	-99.31
BRD-K16551401	PNU-22394	Serotonin receptor agonist	-99.25
BRD-A59985574	topotecan	Topoisomerase inhibitor	-99.22
BRD-K52721684	PCO-400	Potassium channel activator	-99.21
BRD-K46068882	eugenitol	Bacterial quorum sensing inhibitor	-99.11
BRD-K53737926	amitriptyline	Norepinephrine inhibitor	-98.95

COMPOUND ID	COMPOUND NAME	DESCRIPTION	CONECTIVITY SCORE
BRD-K14880289	GW-501516	PPAR receptor agonist	-98.91
BRD-K17110974	aristolochic-acid	Phospholipase inhibitor	-98.89
BRD-K60762818	desipramine	Tricyclic antidepressant	-98.8
BRD-K07888196	tyrphostin-AG-538	IGF-1 inhibitor	-98.76
BRD-K28137194	loreclezole	GABA receptor agonist	-98.44
BRD-K51751936	alfadolone	GABA receptor agonist	-98.41
BRD-K96740444	itopride	Dopamine receptor antagonist	-98.25
BRD-K82562631	tolmetin	Cyclooxygenase inhibitor	-98.25
BRD-K68402494	ML-9	Myosin light chain kinase inhibitor	-98.06
BRD-K67439147	SIB-1893	Glutamate receptor antagonist	-97.81
BRD-K22227508	targinine	Nitric oxide synthase inhibitor	-97.8
BRD-K50311478	tosyl-phenylalanyl-chloromethyl-ketone	Chymotrypsin inhibitor	-97.8
BRD-K61951118	FG-7142	GABA benzodiazepine site receptor inverse agonist	-97.53
BRD-K63979671	etifenin	Compound used in hepatobiliary scans of the liver	-97.4
BRD-K01815685	indole	aryl hydrocarbon receptor agonist	-97.16
BRD-A32949107	MRS-1845	Calcium channel blocker	-96.74
BRD-A77118605	BML-ST330	Phospholipase inhibitor	-96.57

COMPOUND ID	COMPOUND NAME	DESCRIPTION	CONECTIVITY SCORE
BRD-K96263742	GW-7647	PPAR receptor agonist	-96.27
BRD-K82357231	desloratadine	Histamine receptor antagonist	-96.17
BRD-K14200658	syrosingopine	Vesicular monoamine transporter inhibitor	-96.06
BRD-A68929948	DAPT-GSI-IX	Gamma secretase inhibitor	-95.81
BRD-K05181463	L-741626	Dopamine receptor antagonist	-95.71
BRD-A13133631	fluorometholone	Glucocorticoid receptor agonist	-95.62
BRD-A14966924	alaproclate	Serotonin receptor antagonist	-95.57
BRD-K77641333	naphazoline	Adrenergic receptor agonist	-95.41
BRD-K00317371	RITA	MDM inhibitor	-95.24
BRD-K70487031	flupentixol	Dopamine receptor antagonist	-95.14
BRD-K67100011	pivmecillinam	Bacterial cell wall synthesis inhibitor	-95.03
BRD-K99595596	salsolinol	Monoamine oxidase inhibitor	-94.64
BRD-A04327189	synephrine	Adrenergic receptor agonist	-94.38
BRD-K54330070	SB-202190	p38 MAPK inhibitor	-94.12
BRD-A85860691	chaetocin	Histone lysine methyltransferase inhibitor	-94.12
BRD-K02113016	olaparib	PARP inhibitor	-94.11
BRD-K41564320	purvalanol-b	Tyrosine kinase inhibitor	-94

COMPOUND ID	COMPOUND NAME	DESCRIPTION	CONECTIVITY SCORE
BRD-K31627533	rimexolone	Glucocorticoid receptor agonist	-93.94
BRD-K18787491	U-0126	MEK inhibitor	-93.92
BRD-A93424738	dexamethasone	Glucocorticoid receptor agonist	-93.92
BRD-K39944607	ochratoxin-a	Phenylalanyl tRNA synthetase inhibitor	-93.89
BRD-K28761384	zuclopenthixol	Dopamine receptor antagonist	-93.51
BRD-K39915878	loxapine	Dopamine receptor antagonist	-93.3
BRD-K31542390	mycophenolic-acid	Dehydrogenase inhibitor	-93.22
BRD-K32584078	BML-257	AKT inhibitor	-93.13
BRD-K04414442	SB-222200	Tachykinin antagonist	-93.11
BRD-A04756508	norgestimate	Progesterone receptor agonist	-92.96
BRD-K30097969	pitavastatin	HMGCR inhibitor	-92.95
BRD-K81729199	AQ-RA741	Acetylcholine receptor antagonist	-92.71
BRD-K95992530	Cyclo-[Arg-Gly-Asp-D-Phe-Val]	integrin antagonist	-92.28
BRD-K90333595	phentolamine	Adrenergic receptor antagonist	-92.26
BRD-K73991644	isoquercetin	Aldose reductase inhibitor	-92.21
BRD-A90515964	guaifenesin	Expectorant	-92.18
BRD-A17428743	BW-723C86	Serotonin receptor agonist	-92.14

COMPOUND ID	COMPOUND NAME	DESCRIPTION	CONECTIVITY SCORE
BRD-A43940795	tetrahydropalmatine	Serotonin release inhibitor	-92.09
BRD-K83508485	FK-888	Tachykinin antagonist	-91.98
BRD-K84996949	sinensetin	Cyclooxygenase inhibitor	-91.61
BRD-K12244279	MEK1-2-inhibitor	MEK inhibitor	-91.61
BRD-K88551539	CAY-10585	HIF modulator	-91.47
BRD-A02481876	importazole	Importin-beta transport receptor inhibitor	-91.27
BRD-K51941867	LM-1685	Cyclooxygenase inhibitor	-91.15
BRD-A26384407	chlortalidone	Carbonic anhydrase inhibitor	-90.81
BRD-K50135270	GBR-12935	Dopamine uptake inhibitor	-90.76
BRD-K89687904	PKCbeta-inhibitor	PKC inhibitor	-90.74
BRD-K27141178	SB-203186	Serotonin receptor antagonist	-90.41
BRD-K04146668	GW-441756	Growth factor receptor inhibitor	-90.39
BRD-K20197062	SA-94315	Caspase inhibitor	-90.37
BRD-K88611939	aniracetam	Glutamate receptor agonist	-90.03
BRD-K21667562	AM-404	Cyclooxygenase inhibitor	-89.83
BRD-A48570745	ivermectin	GABA receptor agonist	-89.75
BRD-K97399794	quercetin	Polar auxin transport inhibitor	-89.15
BRD-K79930101	GW-583340	EGFR inhibitor	-89.15

COMPOUND ID	COMPOUND NAME	DESCRIPTION	CONECTIVITY SCORE
BRD-K44899736	RO-16-6941	Monoamine oxidase inhibitor	-89.14
BRD-K50398167	meclofenamic-acid	Cyclooxygenase inhibitor	-88.77
BRD-K68341547	W-9	Calmodulin antagonist	-88.39
BRD-K71035033	masitinib	KIT inhibitor	-88.25
BRD-K67080878	milrinone	Phosphodiesterase inhibitor	-88.11
BRD-A78877355	nefopam	Cyclooxygenase inhibitor	-87.98
BRD-K93480852	KN-93	Calcium-calmodulin dependent protein kinase inhibitor	-87.96
BRD-K60060639	methyllidocaine	antiarrhythmic medication	-87.95
BRD-K14441456	tyrphostin-AG-556	EGFR inhibitor	-87.5
BRD-K40530731	hyoscyamine	Acetylcholine receptor antagonist	-87.27
BRD-A47633927	NPC-15199	ICAM1 antagonist	-87.04
BRD-K20655524	mefexamide	Psychoactive drug	-86.87
BRD-K11717138	benzbromarone	Chloride channel blocker	-86.84
BRD-A43882281	pinacidil	ATP channel activator	-86.79
BRD-K84266862	BRL-50481	Phosphodiesterase inhibitor	-86.37
BRD-A93000692	ciglitazone	PPAR receptor agonist	-85.93
BRD-K19284129	salvinorin-a	Opioid receptor agonist	-85.6

COMPOUND ID	COMPOUND NAME	DESCRIPTION	CONECTIVITY SCORE
BRD-K15563106	phloretin	Sodium/glucose cotransporter inhibitor	-85.45
BRD-K92138166	mammea-a	other antibiotic	-85.34
BRD-K31283835	tofacitinib	JAK inhibitor	-85.32
BRD-A62525898	prednisone	Glucocorticoid receptor agonist	-85.3
BRD-A88254928	salbutamol	Adrenergic receptor agonist	-85.2
BRD-K03842655	penitrem-a	Potassium channel blocker	-85.04
BRD-K71430621	clobenpropit	Histamine receptor antagonist	-84.94
BRD-A56987319	SQ-22536	Adenylyl cyclase inhibitor	-84.86
BRD-K55430733	WAY-629	Serotonin receptor agonist	-84.6
BRD-K39391626	ethylestrenol	Progesterone receptor agonist	-84.45
BRD-K90524085	MY-5445	Phosphodiesterase inhibitor	-84.32
BRD-K95885906	quercetagetin	PIM inhibitor	-84.19
BRD-K43149758	myricetin	Androgen receptor agonist	-84.14
BRD-K39462424	dexchlorpheniramine	Histamine receptor antagonist	-84.13
BRD-K74501079	azithromycin	Bacterial 50S ribosomal subunit inhibitor	-83.75
BRD-K81272440	dantrolene	Calcium channel blocker	-83.68
BRD-K11927976	ER-27319	Mediator release inhibitor	-83.45

COMPOUND ID	COMPOUND NAME	DESCRIPTION	CONECTIVITY SCORE
BRD-K83289131	CAY-10618	NAMPT inhibitor	-83.08
BRD-K77171813	proxyfan	Histamine receptor modulator	-83.03
BRD-A26845397	isamoltan	Adrenergic receptor antagonist	-82.83
BRD-K78485176	olmesartan	Angiotensin receptor antagonist	-82.61
BRD-U37049823	HG-6-64-01	RAF inhibitor	-82.57
BRD-A39646320	HC-toxin	HDAC inhibitor	-82.28
BRD-K04923131	GSK-3-inhibitor-IX	Glycogen synthase kinase inhibitor	-82.23
BRD-K65331431	retinyl	vitamin analog	-82.2
BRD-K66093087	FGIN-1-43	Benzodiazepine receptor agonist	-82.12
BRD-K06753942	nobiletin	MEK inhibitor	-82.02
BRD-K28115081	apafant	Platelet activating factor receptor antagonist	-81.77
BRD-A78322124	dobutamine	Adrenergic receptor agonist	-81.25
BRD-K26979635	NS-3694	Glutamate receptor antagonist	-81.21
BRD-U73238814	QL-XI-92	DDR1 inhibitor	-80.96
BRD-K35629949	SR-27897	CCK receptor antagonist	-80.95
BRD-K24576554	AT-9283	JAK inhibitor	-80.88
BRD-A91555231	norepinephrine	Adrenergic receptor agonist	-80.88
BRD-K47983010	BX-795	IKK inhibitor	-80.8

COMPOUND ID	COMPOUND NAME	DESCRIPTION	CONECTIVITY SCORE
BRD-K61829047	7b-cis	Exportin antagonist	-80.56
BRD-K80396088	gliquidone	Sulfonylurea	-80.41
BRD-K56047318	RHC-80267	Triacylglycerol lipase inhibitor	-80.25
BRD-A68039575	liquiritigenin	Aromatase inhibitor	-80.12
BRD-K59184148	SB-216763	Glycogen synthase kinase inhibitor	-79.93
BRD-K45446451	JZL-184	Monoacylglucerol lipase inhibitor	-79.67
BRD-A92670106	tocainide	Sodium channel blocker	-79.6
BRD-K81528515	nilotinib	ABL inhibitor	-79.19
BRD-K14550461	doxercalciferol	Vitamin D receptor agonist	-79.18
BRD-K79404599	enzastaurin	PKC inhibitor	-79.11
BRD-K94176593	TWS-119	Glycogen synthase kinase inhibitor	-79.08
BRD-A84389633	tropanyl-3,5-dimethylbenzoate	Serotonin receptor antagonist	-78.78
BRD-K01493881	apigenin	Casein kinase inhibitor	-78.46
BRD-A69960130	bromocriptine	Dopamine receptor agonist	-78.42
BRD-K18036262	L-168049	Glucagon receptor antagonist	-77.94
BRD-K82147103	lofepramine	Norepinephrine reuptake inhibitor	-77.54
BRD-K52620403	STO-609	Calmodulin antagonist	-77.44
BRD-K67578145	GDC-0879	RAF inhibitor	-77.25

COMPOUND ID	COMPOUND NAME	DESCRIPTION	CONECTIVITY SCORE
BRD-A35588707	teniposide	Topoisomerase inhibitor	-77.05
BRD-K79684402	RO-10-5824	Dopamine receptor agonist	-76.95
BRD-A97437073	rosiglitazone	Insulin sensitizer	-76.76
BRD-K98548675	parthenolide	NFkB pathway inhibitor	-76.71
BRD-K70281171	U-99194	Dopamine receptor antagonist	-75.86
BRD-K33211335	dextromethorphan	Glutamate receptor antagonist	-75.81
BRD-K98490050	amsacrine	Topoisomerase inhibitor	-75.35
BRD-A13807286	HA-14-1	BCL inhibitor	-75.31
BRD-A83855350	naltrexone	Opioid receptor antagonist	-75.31
BRD-K53414658	tivozanib	VEGFR inhibitor	-75.26
BRD-A92439610	triamcinolone	Glucocorticoid receptor agonist	-75.05
BRD-K87158025	benzamil	Sodium channel blocker	-74.96
BRD-A42167015	carteolol	Adrenergic receptor antagonist	-74.85
BRD-K35430135	SR-59230A	Adrenergic receptor antagonist	-74.83
BRD-A09533288	verapamil	Calcium channel blocker	-74.76
BRD-K66353228	zoxazolamine	Myorelaxant	-74.75
BRD-K77625572	etomoxir	Carnitine palmitoyltransferase inhibitor	-74.74
BRD-K96809896	SKF-86002	p38 MAPK inhibitor	-74.61

COMPOUND ID	COMPOUND NAME	DESCRIPTION	CONECTIVITY SCORE
BRD-K49328571	dasatinib	BCR-ABL kinase inhibitor	-74.57
BRD-K17306061	aprepitant	Tachykinin antagonist	-74.53
BRD-A02713983	dihydrodeoxygedunin	Growth factor receptor activator	-74.5
BRD-K50938786	ropivacaine	Sodium channel blocker	-74.48
BRD-K60174629	z-prolyl-prolinal	Prolyl endopeptidase inhibitor	-74.45
BRD-K83144676	olmesartan	Angiotensin antagonist	-74.28
BRD-K40901640	cinanserin	Serotonin receptor antagonist	-74.12
BRD-K50168500	canertinib	EGFR inhibitor	-74.09
BRD-K27871032	lysergol	Ergoline alkaloid	-73.69
BRD-A45889380	mepacrine	Cytokine production inhibitor	-73.6
BRD-K22010301	JLK-6	Gamma secretase inhibitor	-73.43
BRD-K41160163	fenobam	Glutamate receptor antagonist	-73.34
BRD-K49448285	bisindolylmaleimide	CDK inhibitor	-73.34
BRD-K33226500	indinavir	HIV protease inhibitor	-73.32
BRD-A00100033	nifurtimox	DNA inhibitor	-73.29
BRD-A20589515	dihydroxyphenylglycine	Glutamate receptor agonist	-73.28
BRD-K01253243	SB-590885	RAF inhibitor	-73.23
BRD-A34817987	itraconazole	Cytochrome P450 inhibitor	-73.17

COMPOUND ID	COMPOUND NAME	DESCRIPTION	CONECTIVITY SCORE
BRD-K53220666	trimetozine	Sedative	-72.52
BRD-K93258693	GW-9662	PPAR receptor antagonist	-72.18
BRD-K19605405	ZM-241385	Adenosine receptor antagonist	-72.13
BRD-K23875128	RHO-kinase-inhibitor-III[rockout]	Rho associated kinase inhibitor	-71.98
BRD-K47539947	tetradecylthioacetic-acid	Lipid peroxidase inhibitor	-71.87
BRD-A92177080	betamethasone	Glucocorticoid receptor agonist	-71.79
BRD-K62810658	PD-98059	MEK inhibitor	-71.59
BRD-K03600606	catechin	Beta secretase inhibitor	-71.03
BRD-A49370193	RO-60-0175	Serotonin receptor agonist	-70.86
BRD-K37130656	rivaroxaban	Coagulation inhibitor	-70.84
BRD-K38449220	seneciphylline	Cytochrome P450 inhibitor	-70.36
BRD-K33818169	GW-3965	LXR agonist	-70.28
BRD-K89046952	ciclacillin	Bacterial cell wall synthesis inhibitor	-69.79
BRD-K17743697	KB-R7943	Sodium/calcium exchange inhibitor	-69.56
BRD-A54596827	solifenacin	Acetylcholine receptor antagonist	-69.43
BRD-K34092021	arvanil	TRPV agonist	-68.93
BRD-K11630072	carmofur	Thymidylate synthase inhibitor	-68.93

COMPOUND ID	COMPOUND NAME	DESCRIPTION	CONECTIVITY SCORE
BRD-A00520476	otenzepad	Acetylcholine receptor antagonist	-68.89
BRD-K83010055	VU-0415374-1	Glutamate receptor modulator	-68.8
BRD-A59174698	ritodrine	Adrenergic receptor agonist	-68.76
BRD-K18757346	U-46619	Thromboxane receptor agonist	-68.65
BRD-K89125793	tinidazole	Antiprotozoal	-68.44
BRD-K82091397	SB-239063	p38 MAPK inhibitor	-68.28
BRD-K95785537	PP-2	SRC inhibitor	-68.15
BRD-K76133116	benzydamine	Membrane integrity inhibitor	-67.83
BRD-A57457122	VU-0400071-3	Glutamate receptor modulator	-67.74
BRD-A78295502	hydroquinine	Antiarrhythmic	-67.7
BRD-A67373739	AICA-ribonucleotide	AMPK activator	-67.65
BRD-K93034159	cladribine	Adenosine deaminase inhibitor	-67.63
BRD-A18917088	estradiol	Contraceptive agent	-67.34
BRD-K55930204	phenytoin	Hydantoin antiepileptic	-67.08
BRD-K94080537	diethyltoluamide	DEET activator of fly antenna ionotropic receptor IR40a	-67.07
BRD-A82238138	budesonide	Glucocorticoid receptor agonist	-67.05
BRD-A90311807	cilastatin	Dehydropeptidase inhibitor	-67.02
BRD-K62310379	fluticasone	Glucocorticoid receptor agonist	-67.02

COMPOUND ID	COMPOUND NAME	DESCRIPTION	CONECTIVITY SCORE
BRD-K30020243	aliskiren	Antihypertensive	-66.88
BRD-K67298865	SB-431542	TGF beta receptor inhibitor	-66.77
BRD-K56001384	antimycin-a	ATP synthase inhibitor	-66.52
BRD-K75699339	rizatriptan	Serotonin receptor agonist	-66.49
BRD-K60274257	dephostatin	Tyrosine phosphatase inhibitor	-66.38
BRD-K08890269	CO-102862	Sodium channel blocker	-66.35
BRD-K95763993	trapidil	PDGFR receptor inhibitor	-66.29
BRD-K62353524	DY-131	Estrogen receptor agonist	-66.15
BRD-A39969961	eplerenone	Cytochrome P450 inhibitor	-66.12
BRD-K15108141	gemcitabine	Ribonucleotide reductase inhibitor	-66.08
BRD-K47943470	tyrphostin-51	EGFR inhibitor	-66.05
BRD-K82865713	prostaglandin-b2	cAMP inhibitor	-65.89
BRD-A29731977	17-hydroxyprogesterone-caproate	progesterone receptor agonist	-65.79
BRD-K76587808	fraxetin	Antioxidant	-65.73
BRD-K52930707	rescinnamine	ACE inhibitor	-65.71
BRD-A48720949	testosterone	androgen receptor agonist	-65.65
BRD-K99616396	motesanib	KIT inhibitor	-65.59
BRD-K31491153	1-phenylbiguanide	Serotonin receptor agonist	-64.83

COMPOUND ID	COMPOUND NAME	DESCRIPTION	CONECTIVITY SCORE
BRD-A42553870	L-152804	Neuropeptide receptor antagonist	-64.83
BRD-K32311154	nifekalant	Potassium channel blocker	-64.77
BRD-A22769835	homochlorcyclizine	Antihistamine	-64.77
BRD-K49481516	galantamine	Acetylcholinesterase inhibitor	-64.73
BRD-K31238592	devazepide	CCK receptor antagonist	-64.64
BRD-K93280214	gabazine	GABA receptor antagonist	-64.5
BRD-A93206962	L-755507	Adrenergic receptor agonist	-64.37
BRD-K00532621	midazolam	Benzodiazepine receptor agonist	-64.25
BRD-A33711280	metixene	Acetylcholine receptor antagonist	-64.16
BRD-K16478699	PLX-4720	RAF inhibitor	-64.1
BRD-K32292990	CGP-53353	EGFR inhibitor	-63.99
BRD-K29673530	hypericin	Tyrosine kinase inhibitor	-63.96
BRD-A01295252	trans-7-hydroxy-pipat	Dopamine receptor ligand	-63.85
BRD-K03981224	ethisterone	Progestogen hormone	-63.68
BRD-A09539288	homatropine	Acetylcholine receptor antagonist	-63.68
BRD-K21971034	OM-137	Aurora kinase inhibitor	-63.64
BRD-K64935403	ebelactone-b	Lipase inhibitor	-63.49
BRD-K62056274	quipazine	Serotonin receptor agonist	-63.41

COMPOUND ID	COMPOUND NAME	DESCRIPTION	CONECTIVITY SCORE
BRD-K94841585	emodic-acid	Laxative	-63.21
BRD-K51541829	RO-25-6981	Ionotropic glutamate receptor antagonist	-63.14
BRD-K29668683	BD-1063	Sigma receptor antagonist	-63.06
BRD-A66563878	medetomidine	Adrenergic receptor agonist	-62.8
BRD-K19136521	indirubin	CDK inhibitor	-62.74
BRD-K63784565	BRD-K63784565	Topoisomerase inhibitor	-62.65
BRD-U97083655	teicoplanin	Bacterial cell wall synthesis inhibitor	-62.63
BRD-K35531059	molsidomine	Guanylyl cyclase activator	-62.12
BRD-K13390322	AT-7519	CDK inhibitor	-62.03
BRD-K24675965	LY-288513	CCK receptor antagonist	-62.03
BRD-A39522003	OMDM-2	FAAH inhibitor	-62
BRD-K93080877	Ala-Ala-Phe-CMK	Tripeptidyl peptidase inhibitor	-61.97
BRD-A41941932	vitexin	Antioxidant	-61.77
BRD-K14329163	BAY-K8644	Calcium channel activator	-61.49
BRD-A43974499	reboxetine	Adrenergic receptor antagonist	-61.31
BRD-K67261995	adipiodone	Contrast agent	-61.29
BRD-K49810818	sorafenib	FLT3 inhibitor	-61.21

COMPOUND ID	COMPOUND NAME	DESCRIPTION	CONECTIVITY SCORE
BRD-K45401373	betulinic-acid	Apoptosis stimulant	-61.07
BRD-K53523901	arctigenin	MEK inhibitor	-60.94
BRD-K09537769	NU-7026	DNA dependent protein kinase inhibitor	-60.85
BRD-K68332390	ponalrestat	Aldose reductase inhibitor	-60.84
BRD-K30296925	flavokavain-b	Antineoplastic	-60.69
BRD-A70083328	secnidazole	Acetylcholinesterase inhibitor	-60.66
BRD-K33312228	halometasone	Glucocorticoid receptor agonist	-60.52
BRD-K06198550	isorotenone	Mitochondrial complex I inhibitor	-60.3
BRD-K94441233	mevastatin	HMGCR inhibitor	-60.2
BRD-A73741725	exemestane	Aromatase inhibitor	-59.84
BRD-K87510569	RS-504393	CC chemokine receptor antagonist	-59.64
BRD-K02407574	parbendazole	Tubulin inhibitor	-59.6
BRD-K11540476	EMF-BCA1-64	Caspase inhibitor	-59.58
BRD-K00312224	PPT	Estrogen receptor agonist	-59.55
BRD-K34014345	naproxol	Anti-inflammatory	-59.51
BRD-K90259198	W-7	Calmodulin antagonist	-59.43
BRD-K07220430	cinnarizine	Calcium channel blocker	-59.43
BRD-K60923938	veratridine	Sodium channel activator	-59.03

COMPOUND ID	COMPOUND NAME	DESCRIPTION	CONECTIVITY SCORE
BRD-A55756846	H-7	PKA inhibitor	-59.02
BRD-K27499107	carbacyclin	IP receptor activator	-58.96
BRD-K71499074	diclofenamide	Carbonic anhydrase inhibitor	-58.84
BRD-A70649075	sulconazole	Sterol demethylase inhibitor	-58.44
BRD-K97752965	nicorandil	Nitric oxide donor	-58.04
BRD-A80960055	celastrol	Anti-inflammatory	-57.46
BRD-K31471398	dihydrexidine	Dopamine receptor agonist	-57.39
BRD-K43389675	daunorubicin	RNA synthesis inhibitor	-57.35
BRD-K28907958	CD-437	Retinoid receptor agonist	-57.31
BRD-A97479839	piperidolate	Acetylcholine receptor antagonist	-57.24
BRD-K14993104	bemegride	Chemoreceptor agonist	-57.23
BRD-K18618618	cimetidine	Histamine receptor antagonist	-57.15
BRD-A07000685	hydrocortisone	Glucocorticoid receptor agonist	-57.13
BRD-K94689771	pinocembrin	CYP1B1 inhibitor	-57.1
BRD-K50464341	berbamine	Calmodulin antagonist	-57.1
BRD-A72703248	SKF-96365	Calcium channel blocker	-57.06
BRD-K70241288	L-692585	Growth hormone releasing peptide ligand agonist	-56.85
BRD-A31204924	mitotane	Antineoplastic	-56.61

COMPOUND ID	COMPOUND NAME	DESCRIPTION	CONECTIVITY SCORE
BRD-K57930253	nitrazepam	Benzodiazepine receptor agonist	-56.56
BRD-K04430056	7-nitroindazole	nitric oxide synthase inhibitor	-56.5
BRD-A25569250	KI-16425	Lysophosphatidic acid receptor antagonist	-56.49
BRD-K81916719	triclabendazole	Microtubule inhibitor	-56.49
BRD-U08759356	EI-346-erlotinib-analog	EGFR inhibitor	-56.42
BRD-A89337244	PD-102807	Acetylcholine receptor antagonist	-56.39
BRD-A45140972	meclocycline	Bacterial 30S ribosomal subunit inhibitor	-56.28
BRD-K32830106	guanfacine	Adrenergic receptor agonist	-56.08
BRD-A15415227	GW-1929	PPAR receptor agonist	-56.07
BRD-K29359156	ebselen	H+/K+-ATPase inhibitor	-55.76
BRD-A92585442	RU-28318	Cytochrome P450 inhibitor	-55.62
BRD-A62434282	goserelin	Gonadotropin releasing factor hormone receptor agonist	-55.53
BRD-K30990140	FR-122047	Cyclooxygenase inhibitor	-55.34
BRD-K13810148	givinostat	HDAC inhibitor	-55.33
BRD-A09062839	amylocaine	Local anesthetic	-55.32
BRD-K10176267	L-701252	Glutamate receptor antagonist	-55.27
BRD-A65076780	dihydroergocristine	Adrenergic receptor antagonist	-55.15

COMPOUND ID	COMPOUND NAME	DESCRIPTION	CONECTIVITY SCORE
BRD-K05181084	NGB-2904	Dopamine receptor antagonist	-55.08
BRD-K48932581	cetraxate	Mucus protecting agent	-54.8
BRD-K47780086	penciclovir	DNA directed DNA polymerase inhibitor	-54.77
BRD-A24381660	zeranol	Estrogen receptor agonist	-54.73
BRD-K82941592	rosuvastatin	HMGCR inhibitor	-54.69
BRD-A75552914	isoxicam	Cyclooxygenase inhibitor	-54.62
BRD-A47494775	dipivefrine	Adrenergic receptor agonist	-54.61
BRD-K92726801	hydrastinine	Haemostatic agent	-54.06
BRD-A47706533	L-BSO	Glutathione transferase inhibitor	-53.83
BRD-K28183345	proguanil	Dihydrofolate reductase inhibitor	-53.75
BRD-A82590476	SDZ-NKT-343	Tachykinin antagonist	-53.59
BRD-K63151507	MNITMT	Lymphocyte inhibitor	-53.51
BRD-K34820100	tebuthiuron	Photosynthesis inhibitor	-53.27
BRD-A68631409	evodiamine	ATPase inhibitor	-53.17
BRD-K63504947	semaxanib	VEGFR inhibitor	-53.14
BRD-A39747742	estradiol-valerate	Estrogen receptor agonist	-53.04
BRD-A52650764	ingenol	PKC activator	-53
BRD-K82484965	carmoxirole	Dopamine receptor agonist	-52.96

COMPOUND ID	COMPOUND NAME	DESCRIPTION	CONECTIVITY SCORE
BRD-A62021152	WAY-161503	Serotonin receptor agonist	-52.88
BRD-K30240666	clemastine	Histamine receptor antagonist	-52.88
BRD-K96084870	DMBI	PDGFR receptor inhibitor	-52.67
BRD-K50866992	tropisetron	Serotonin receptor antagonist	-52.65
BRD-A41555725	chlortetracycline	Protein synthesis inhibitor	-52.52
BRD-K36529613	PU-H71	HSP inhibitor	-52.42
BRD-A64977602	mirtazapine	Adrenergic receptor antagonist	-52.3
BRD-K88679075	methandriol	Androgenic steroid	-52.23
BRD-K89014967	AS-703026	MEK inhibitor	-52.19
BRD-A19736161	ondansetron	Serotonin receptor antagonist	-52.18
BRD-K46469693	SCH-442416	Adenosine receptor antagonist	-52.15
BRD-K08619838	tremorine	Acetylcholine receptor agonist	-52.11
BRD-K43164539	cholic-acid	Bile acid	-51.97
BRD-K49865102	PD-0325901	MEK inhibitor	-51.79
BRD-K82381502	acetylcholine	Acetylcholine receptor agonist	-51.66
BRD-K57011718	UK-356618	Metalloproteinase inhibitor	-51.49
BRD-K01095011	finasteride	5-alpha reductase inhibitor	-51.37
BRD-K82255054	propofol	GABA receptor agonist	-51.27

COMPOUND ID	COMPOUND NAME	DESCRIPTION	CONECTIVITY SCORE
BRD-A46179541	doxapram	Potassium channel blocker	-51.19
BRD-A93659613	GR-89696	Opioid receptor agonist	-51.1
BRD-A00758722	noretynodrel	Progestogen hormone	-51.01
BRD-A87125127	3-matida	Glutamate receptor antagonist	-50.72
BRD-A92161634	scopoline	Acetylcholine receptor antagonist	-50.72
BRD-K46056750	AZD-7762	CHK inhibitor	-50.71
BRD-K05673000	dicloxacillin	Bacterial cell wall synthesis inhibitor	-50.65
BRD-K42748308	XE-991	Potassium channel blocker	-50.42
BRD-K25875056	SC-9	Protein tyrosine kinase activator	-50.36
BRD-A00267231	hemado	Adenosine receptor agonist	-50.33
BRD-K72029282	probucol	Atherogenesis inhibitor	-50.08
BRD-K17497770	butein	EGFR inhibitor	-49.93

Table S14. Perturbations CLUE Query (v.1.1) analysis for selected up-regulated and downregulated genes. Output table of the CLUE Query (v.1.1) analysis for selected 150 up-regulated genes an all downregulated. The table shows the perturbations with negative connectivity score (median_tau_score).

Perturbation Type	Median_tau_score
Lipocalins GOF	-76.24
NFKB Activation GOF	-73.97
PDGFR/KIT inhibitor	-72.86
WNT family LOF	-71.81
RAF inhibitor	-71.38
CCK receptor antagonist	-68.94
MEK inhibitor	-65.87
PKC inhibitor	-61.56
NADH ubiquinone oxidoreductase core subunits GOF	-55.82
Topoisomerase inhibitor	-50.09
Tachykinin antagonist	-46.33
Ubiquitin-specific peptidases LOF	-46.06
SRC inhibitor	-45.46
Glycogen synthase kinase inhibitor	-45.36
P38 MAPK inhibitor	-45.15
DNA Replication LOF	-42.82
Integrin subunits alpha LOF	-42.07

Perturbation Type	Median_tau_score
Rho GTPase activating proteins LOF	-39.52
EMSY complex LOF	-36.61
MDM inhibitor	-36.05
Bile acid	-35.08
ATP synthase inhibitor	-30.88
Serotonin receptor agonist	-30.83
DNA synthesis inhibitor	-30.60
PPAR receptor agonist	-30.47
PARP inhibitor	-28.21
Androgen receptor modulator	-26.14
Toll like receptors LOF	-25.29
Ribonucleotide reductase inhibitor	-24.59
PKC activator	-24.05
PKA inhibitor	-23.33
Thromboxane receptor antagonist	-22.94
Structural maintenance of chromosomes proteins LOF	-19.09
Bacterial DNA gyrase inhibitor	-18.56
LIM class homeoboxes GOF	-18.38

Perturbation Type	Median_tau_score
Thymidylate synthase inhibitor	-18.35
Sigma receptor antagonist	-17.23
Mitochondrial complex IV LOF	-17.22
Progesterone receptor antagonist	-15.62
Estrogen receptor agonist	-15.59
IMPDH inhibitor	-15.27
Mitogen activated protein kinases LOF	-13.94
Sirtuins GOF	-13.28
HRH1 antagonist	-13.19
Integrin subunits beta LOF	-13.06
Aromatase inhibitor	-12.98
HMGCR inhibitor	-12.66
V type ATPases LOF	-12.20
FLT3 inhibitor	-11.94
Na-K-Cl transporter inhibitor	-9.28
Progesterone receptor agonist	-9.21
Interleukins GOF	-8.70
Tumor necrosis factor superfamily LOF	-8.62

Perturbation Type	Median_tau_score
DNA polymerase inhibitor	-8.51
X linked mental retardation group 2 LOF	-7.79
Potassium channel blocker	-7.60
KCNJ11 modulator	-6.50
Protein phosphatase catalytic subunits LOF	-6.33
Phospholipases GOF	-6.31
NFkB pathway inhibitor	-6.29
DNA dependent protein kinase inhibitor	-6.12
CDK inhibitor	-5.79
FGFR inhibitor	-4.69
HSP inhibitor	-4.13
Potassium channel activator	-3.47
Angiotensin receptor antagonist	-3.38
Glucocorticoid receptor agonist	-3.02
Lysine acetyltransferases LOF	-2.51
EGFR inhibitor	-1.99
Histone deacetylases LOF	-1.70
Supressors of cytokine signaling GOF	-1.12

Perturbation Type	Median_tau_score
Cyclooxygenase inhibitor	-1.09
AHSP Pathway LOF	-0.94
HOXL subclass homeoboxes LOF	-0.91
Estrogen receptor antagonist	-0.72
Dopamine receptor antagonist	-0.34
Imidazoline ligand	-0.32

Table S15. IC50 of selected drugs used to perform the *in vitro* assays on detailed cell line.

DRUG	CELL_LINE	IC50(µM)	95%CI*	
			error+	*error-*
**IRINOTECAN**	CT26	40	5,81	5,67
	$CT26^{5FUR}$	39	5,82	5,20
	HCT116	46	6,89	10,79
	$HCT116^{5FUR}$	38	8,65	6,42
**IVERMECTIN**	CT26	22	1,43	1,30
	$CT26^{5FUR}$	30	2,03	1,71
	HCT116	27	4,48	3,93
	$HCT116^{5FUR}$	19	6,20	5,10
**AMITRIPTILYNE**	CT26	40	2,34	1,11
	$CT26^{5FUR}$	36	1,05	7,06
	HCT116	33	2,25	7,45
	$HCT116^{5FUR}$	40	7,78	1,87
**1A-116**	CT26	31	1,53	2,03
	$CT26^{5FUR}$	22	4,25	4,94
	HCT116	31	9,26	9,18
	$HCT116^{5FUR}$	29	2,41	8,01

* CONFIDENCE INTERVAL
